# Lessons learned from descriptions and evaluations of knowledge translation platforms supporting evidence-informed policy-making in low- and middle-income countries: a systematic review

**DOI:** 10.1186/s12961-020-00626-5

**Published:** 2020-10-31

**Authors:** Arun C. R. Partridge, Cristián Mansilla, Harkanwal Randhawa, John N. Lavis, Fadi El-Jardali, Nelson K. Sewankambo

**Affiliations:** 1 Department of Medicine, Cumming School of Medicine, University of Calgary, Calgary, Canada; 2 McMaster Health Forum and Health Policy PhD Program, McMaster University, Hamilton, Canada; 3 Michael G. DeGroote School of Medicine, McMaster University, Hamilton, Canada; 4 McMaster Health Forum and Department of Health Research Methods, Evidence and Impact, McMaster University, Hamilton, Canada; 5 Africa Centre for Evidence, University of Johannesburg, Johannesburg, South Africa; 6 Knowledge to Policy Center and Department of Health Management and Policy, American University of Beirut, Beirut, Lebanon; 7 Clinical Epidemiology and Biostatistics Unit, Department of Medicine, College of Health Sciences, Makerere University, Kampala, Uganda

**Keywords:** Knowledge translation, Evidence-informed policy, Health systems, Systematic evaluation

## Abstract

**Background:**

Knowledge translation (KT) platforms are organisations, initiatives and networks that focus on supporting evidence-informed policy-making at least in part about the health-system arrangements that determine whether the right programmes, services and products get to those who need them. Many descriptions and evaluations of KT platforms in low- and middle-income countries have been produced but, to date, they have not been systematically reviewed.

**Methods:**

We identified potentially relevant studies through a search of five electronic databases and a variety of approaches to identify grey literature. We used four criteria to select eligible empirical studies. We extracted data about seven characteristics of included studies and about key findings. We used explicit criteria to assess study quality. In synthesising the findings, we gave greater attention to themes that emerged from multiple studies, higher-quality studies and different contexts.

**Results:**

Country was the most common jurisdictional focus of KT platforms, EVIPNet the most common name and high turnover among staff a common infrastructural feature*.* Evidence briefs and deliberative dialogues were the activities/outputs that were the most extensively studied and viewed as helpful, while rapid evidence services were the next most studied but only in a single jurisdiction. None of the summative evaluations used a pre–post design or a control group and, with the exception of the evaluations of the influence of briefs and dialogues on intentions to act, none of the evaluations achieved a high quality score.

**Conclusions:**

A large and growing volume of research evidence suggests that KT platforms offer promise in supporting evidence-informed policy-making in low- and middle-income countries. KT platforms should consider as next steps expanding their current, relatively limited portfolio of activities and outputs, building bridges to complementary groups, and planning for evaluations that examine ‘what works’ for ‘what types of issues’ in ‘what types of contexts’.

## Background

### Evidence-informed policy-making

Our definition of evidence-informed policy-making has two parts. The first part involves using the best available data and research evidence – systematically and transparently – in the time available in each of the four phases of the policy-making process [1], namely (1) prioritising problems and understanding their causes (i.e. agenda-setting); (2) deciding which option to pursue (i.e. policy or programme development); (3) ensuring that the chosen option makes an optimal impact at acceptable cost (i.e. policy or programme implementation); and (4) monitoring implementation and evaluating impact. The second part of the definition involves recognising that the data and research evidence will be used alongside the institutional constraints, interest-group pressure, citizen values and other sources of ideas that influence the policy-making process (i.e. policy-making takes place within a political context) [1]. A democratically elected politician typically wants to know that there is a compelling problem (with well understood causes) and a viable policy or programme option (with significant benefits, minimal-to-no harms and acceptable costs), and that the political climate is right, before taking action [2, 3]. The politician’s advisors may also want to know about implementation, monitoring and evaluation plans [3].

The organisations, initiatives and networks supporting evidence-informed health policy-making can be differentiated, based on previous works by the authors, according to which phase(s) of the policy-making process they focus on and whether that focus is about: (1) clinical programmes, services and products (e.g. prescription drugs) that target individuals; (2) public health programmes and services that target groups and populations; and/or (3) health system (i.e. governance, financial and delivery) arrangements that determine whether the right programmes, services and products get to those who need them, in ways that improve population health and the patient or citizen experience, while keeping per capita costs manageable [4, 5].

Consider the following six examples, which complement the type of organisation that is the focus of this study, described in greater detail in the next section [6]:

- most data-analytics organisations focus on (1) understanding policy problems and possibly monitoring implementation and (2) clinical or public health topics;
- most clinical practice guideline initiatives focus on (1) informing which programmes, services and products clinicians should provide (i.e. policy or programme development) and (2) clinical practices;
- most health technology assessment (HTA) networks focus on (1) informing which programmes, services and products a health system should provide and (2) clinical and, less commonly, public health ‘technologies’;
- most modelling organisations focus on (1) estimating the expected reach and impact of selected practices/technologies and related financial, human, and other resource needs and (2) practices/technologies and, less commonly, health system arrangements;
- most implementation research/behavioural insights initiatives focus on (1) informing which implementation approach a health system should use to improve the reach and impact of selected practices/technologies and (2) health system arrangements; and
- most evaluation networks focus on (1) understanding the impact of selected policy choices (e.g. practices/technologies) and (2) clinical or public health topics.

### Knowledge translation (KT) platforms

Our focus here is what we call KT platforms, which we define as organisations, initiatives and networks that focus on supporting evidence-informed policy-making at least in part about the governance, financial and delivery arrangements that determine whether the right programmes, services and products get to those who need them (i.e. supporting the use of research evidence in health systems policy-making) [7–9]. While not a part of our formal definition (or, as we describe in the Methods section, not part of our eligibility criteria), these KT platforms also typically (1) focus on three of the four phases of the policy-making process (i.e. clarifying problems, framing options and identifying implementation considerations), as opposed to prioritising a single phase; (2) use existing data analyses and existing systematic reviews of the available research evidence when possible (much like guideline initiatives and HTA networks), as opposed to conducting new data analyses, modelling exercises, implementation studies or impact evaluations; (3) use a broad range of approaches to making available and supporting the use of the best available data and research evidence, often alongside systematically elicited insights from policy-makers and stakeholders (and values from citizens, less commonly) and on timelines of hours and days to weeks and months, as opposed to a single approach, with evidence only and on timelines of years; and (4) consider their success in terms of informing the policy-making process as opposed to securing peer-reviewed grants and publishing peer-reviewed papers [7–9].

Our focus is specifically KT platforms in low- and middle-income countries (LMICs), where policy-making in over-burdened and under-resourced health systems may present particular challenges, including [10, 11] (1) institutional constraints, such as colonialism-related policy legacies (e.g. weak state capacity for using key policy instruments and for policy implementation, weak civil society groups) and informal institutions (e.g. ‘big-man presidentialism’); (2) unique forms of interest-group pressure, such as external donors (and arguably international organisations and other bodies influenced by these donors), multi-national firms (e.g. natural resource companies) and ethnocultural (e.g. tribal) groups as well as public sector corruption in some countries; (3) limitations in the availability and reliability of key sources of ideas, such as local data and research, and media coverage; and (4) influences external to the health sector such as limited global market for low-cost technologies.

While our interest in KT platforms arose from our involvement in the WHO-sponsored Evidence-Informed Policy Networks (EVIPNet) or in similar entities seen as peers to EVIPNet [8, 12, 13], our focus is not limited to any particular type of organisation, initiative or network. Moreover we have not constrained ourselves to particular health or political system contexts (e.g. national health services with centralised policy authority, multi-party political systems); infrastructures (e.g. whether the group is ‘embedded’ within a ministry of health or located in a university or other independent body, minimum team size and diverse composition); approaches (e.g. building demand for data and evidence and the capacity to find and use them, packaging data and evidence and ‘pushing’ it to those who need it); or measures of outcomes and impact (e.g. more policy-relevant research evidence available, specific measures of evidence use).

### Describing and evaluating KT platforms

Describing and evaluating KT platforms becomes increasingly difficult as one moves from (1) description through formative evaluation and on to summative evaluation and (2) activities and outputs (i.e. the approaches used) through outcomes and impacts (i.e. whether the approaches are making a difference) and on to context and infrastructure (i.e. whether the health and political system context and the KT platform’s infrastructure influence what approaches are used and whether these approaches translate into outcomes and impact).

When it comes to summative evaluation, for example, the KT field continues to search for the ‘holy grail’ of outcome and impact measures that are light touch and can be applied across both ‘intervention’ and ‘control’ groups [14]. The gold standard is widely understood to be multiple case studies examining the influence of an approach or suite of approaches on policy decisions using key-informant interviews, documentary analyses and media analyses that, together, can (1) disentangle instrumental (i.e. direct) uses of research evidence and conceptual uses of research evidence (i.e. changing thinking about a problem or option) from political uses of research evidence (i.e. ‘after the fact’ use of research evidence to justify a choice made for other reasons); (2) rule out confounding influences (i.e. competing variables); and (3) address attribution (i.e. the role played by the approach or the KT platform more generally) [10, 11]. When it comes to evaluating whether and how context and infrastructure affect such relationships, one immediately comes up hard against a sample-size challenge — finding enough health and political system contexts (i.e. unitary states or sub-national jurisdictions in federal states) and infrastructures (i.e. organisational design) supporting a common approach that can be evaluated and willing to use a common evaluation method [15].

**Fig. 1:**
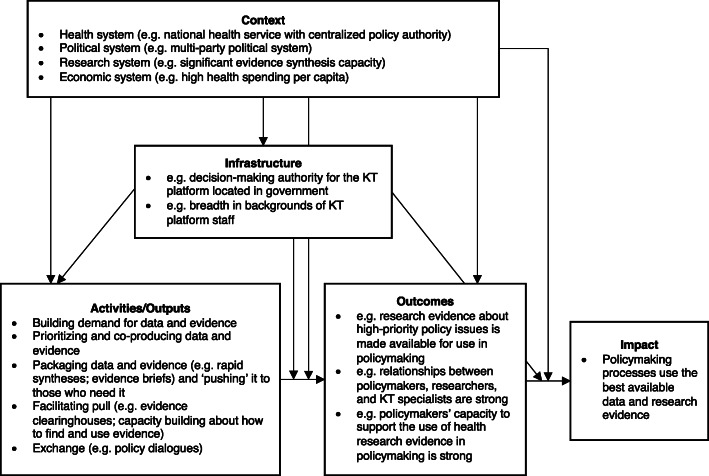
Illustration of the variables and their potential inter-relationships [10, 11, 16–18]

Many descriptions and evaluations of KT platforms in LMICs have been produced but, to date, they have not been systematically reviewed. The objective of this systematic review is to describe the findings of empirical studies that (1) describe the activities and outputs of KT platforms; (2) formatively evaluate these activities and outputs; (3) summatively evaluate whether activities and outputs achieve outcomes and impacts; (4) describe the KT platforms’ context and infrastructure; and (5) examine other types of linkages among variables (which we call ‘linkage evaluations’), such as (a) context and infrastructure influencing whether activities and outputs achieve outcomes and impacts; (b) context influencing decisions about infrastructure design, activities and outputs selected, or the baseline measure of key outcomes; and (c) infrastructure influencing decisions about activities and outputs selected. We provide an illustration of these variables and their potential inter-relationships in Fig. 1, using (1) the Cochrane KT framework to organise findings about activities and outputs [16]; (2) the EVIPNet Monitoring and Evaluation Framework to illustrate (but not limit) the potential outcomes [18]; (3) the Health Systems Evidence framework (governance, financial and delivery arrangements) to organise findings about health-system contexts and about the KT platform’s infrastructure [17]; and (4) the 3I+E framework (institutions, interests, ideas and external factors) to organise findings about political system contexts [10, 11].

## Methods

We used the Preferred Items for the Reporting of Systematic Review and Meta-Analysis (PRISMA) statement to guide the design of the systematic review [19]. We did not require approval from a research ethics board to conduct the review and no external funding was drawn upon to support this review. Several members of the research team are actively involved in operating, supporting and evaluating KT platforms; however, and as noted below, we took steps to ensure that these team members were not involved in the execution of several key steps in the review.

### Identifying potentially relevant studies

We began developing our search parameters by identifying synonyms for three conceptual domains relevant to our review question (and combining synonyms within a domain with ‘OR’ and then across domains with ‘AND’), namely knowledge translation, policy-making and LMICs (which we addressed using a filter developed by the Norwegian satellite of Cochrane’s Effective Practice and Organisation of Care review group in 2012) [20]. We did not include restrictions for language or date but we did apply limits for three keywords (antibiotic, cancer and treatment) that yielded many clinically focused studies that were not relevant. We then worked with a health sciences librarian at McMaster University to iteratively improve our search parameters in light of the performance of our searches, including whether they were identifying a set of articles that we anticipated would meet our eligibility criteria. Finally, we adjusted the search parameters to each electronic database as needed. We provide the search string for MEDLINE in Additional file 1 as an illustration of our search parameters.

We conducted searches in five electronic databases, namely Cumulative Index to Nursing and Allied Health (CINAHL), Embase, Global Health, MEDLINE and Web of Science. Searches were conducted on two separate occasions, first in January 2015 (week 2) and again in September 2016 (week 4) to capture studies published in the intervening time period. We complemented the electronic database searches with a variety of approaches to identify additional literature (including grey literature), namely requests for studies sent to experts in the field; manual review of the reference lists of included studies; ‘related articles’ search in PubMed for all included studies (in July 2017); and manual review of a list of studies published by EVIPNet affiliates, whether or not EVIPNet was the focus of the evaluation, that was maintained by the study authors as part of their evaluation work (also in July 2017).

### Selecting eligible studies

We used four criteria to assess eligibility for inclusion in the review: (1) does the article discuss (a) organisations, initiatives or networks located in or targeted at (b) LMICs whose goal is at least in part to (c) support the use of research evidence in health systems policy-making?; (2) does the article have a methods section?; (3) does the article report empirical data based on the application of these methods?; and (4) do the empirical data pertain to context, infrastructure, activities/outputs, outcomes and/or impacts of these organisations, initiatives and networks?

All criteria needed to be met in order to be included in the systematic review. We did not exclude articles using specific exclusion criteria such as studies in non-peer-reviewed publications (i.e. what is sometimes called grey literature).

We assessed eligibility in three phases: (1) title and abstract review, which was completed by one reviewer (ACRP) on the full sample and by a second reviewer (HR) on a 20% sample; (2) first full-text article review, which was again completed by one reviewer (ACRP) on the full sample and by a second reviewer (HR) on a 20% sample; and (3) a second full-text article review, which was completed by three reviewers (ACRP, HR and JNL) on all articles that were short-listed for inclusion (i.e. that made it through the first full-text article review), to ensure that criteria 1 and 4, which were the most difficult to judge, were appropriately assessed. Any disagreements that arose among the reviewers were resolved by consensus. We calculated the agreement between reviewers on the two 20% samples using Fleiss’ Kappa coefficient. The Fleiss’ Kappa coefficient on the two 20% samples were 0.66 and 0.71, respectively. We retained a list of ‘near miss’ papers that were excluded in case a reader wanted to double-check the application of our inclusion criteria. These are available in Additional file 2.

### Extracting data from studies

We extracted data about the following characteristics of included studies: (1) lead author, year and citation; (2) jurisdictional focus of KT platform(s); (3) name(s) of KT platform(s); (4) categories of variables and/or relationships addressed (i.e. descriptive findings, formative evaluations, summative evaluations and linkages evaluations about KT platforms’ context, infrastructure, activities and outputs, outcomes, and impacts); (5) time period studied; (6) data collection method(s) used; and (7) objective(s). We also extracted the key findings from included studies, with a focus on the variables and relationships described above (e.g. findings about activities and outputs were extracted and organised using the Cochrane KT framework subheadings). When more than one article described the same empirical study, we treated them as a single study for data-extraction purposes only if the same data were re-presented across articles. After extensive pilot testing, data were extracted by one reviewer who was not involved in any of the studied KT platforms (ACRP). A second reviewer (CM) checked all extracted data and resolved any concerns through discussion with the first reviewer.

### Assessing the quality of studies

For all included studies, we used the following explicit criteria to assess quality, with a focus on those including a formative and/or summative evaluation: (1) two or more data collection methods were used (because corroboration across multiple information sources enhances the credibility of studies of policy-making, where there can be incentives to reporting or documenting the rationale for a decision in a particular manner) [21, 22]; (2) a random or purposive sampling strategy was employed (because jurisdictions, cases, key informants and documents must either be representative of the study population from which they are drawn or, in the case of qualitative research, their selection must be well reasoned) [21]; (3) the response rate was greater than 60% (because higher response rates suggest that samples – of key informants, for example – are not biased); (4) two or more types of evidence use were examined (because studies that do not distinguish among instrumental, conceptual and tactical uses of research evidence are likely to miss or conflate politically important differences in how research evidence is used); and (5) two or more competing variables were examined (because studies that fail to examine factors like institutional constraints and interest-group pressure are likely to misrepresent the factors that influence the use of research evidence in policy-making) [10].

We used the first three criteria to assess descriptive studies and formative evaluations (yielding a score out of three) and all five criteria to assess summative evaluations that measured impact (yielding a score out of five). We did not use more traditional risk-of-bias criteria (e.g. random sequence generation, blinding of participants and personnel) to assess the summative evaluations because none of the included studies used formal effectiveness designs such as a randomised controlled trial. We specified cases where scores for individual formative, summative and/or linkage evaluations were different from overall study scores. Articles were deemed to be high quality if they received a score of at least two points if they were assessed using the first three criteria or at least three points if they were assessed using all five criteria. After extensive pilot testing, quality was assessed by one reviewer who is involved with a KT platform but not one that has yet been studied (CM) and was checked by a second reviewer (ACRP). Discrepancies were resolved through discussion between the reviewers and any conflicts were reviewed and resolved with a third reviewer (JNL).

### Synthesising the findings

In synthesising the findings, we gave greater attention to themes that emerged from (1) multiple studies, (2) higher-quality studies and (3) different contexts. The first draft of the synthesis was completed by one reviewer who was not involved in any of the studied KT platforms (ACRP) and the second and final drafts were completed in conjunction with a second reviewer (JNL).

## Results

**Fig. 2:**
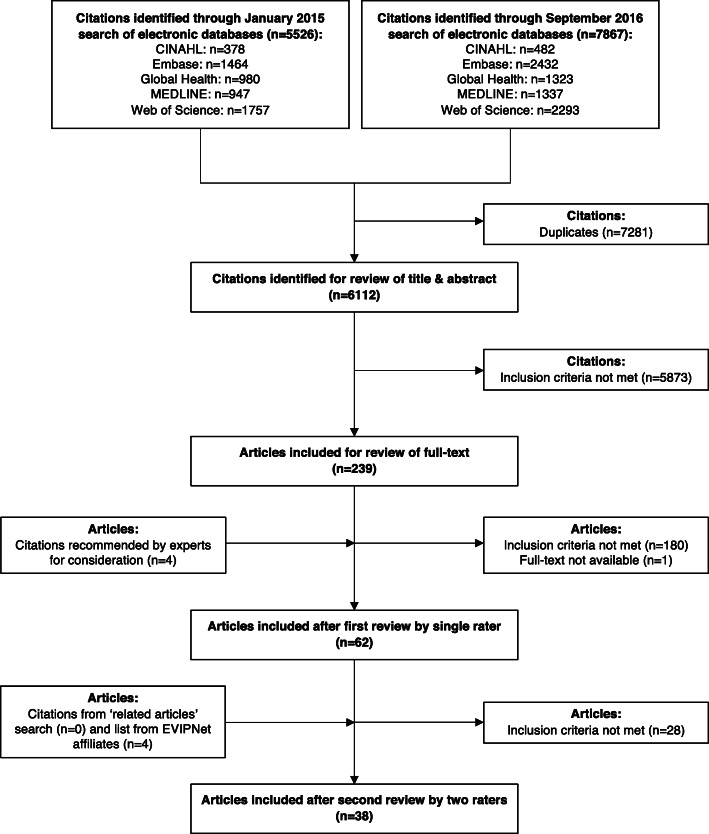
PRISMA flow diagram

As illustrated in Fig. 2 (PRISMA flow diagram), we began with 5526 potentially relevant articles from our first search, 7867 articles from our second search and eight articles from other sources. We included 38 articles after completing all phases of the eligibility assessment [23–60]. We provide, in Additional file 2, the citations for ‘near miss’ papers that were excluded, which appear in the form of two lists: 41 were identified as part of the first full-text article review (and were a subset of the 180 articles excluded at this stage) of the results from both searches (and by one reviewer) and 28 were identified as part of the second full-text article review of the results from both searches (and by three reviewers). Given that we consider the second list to be true ‘near misses’, we only report these ones in the PRISMA flow diagram.

### Characteristics of included studies

Based on our analysis of the characteristics of included studies provided in Additional file 3, we note the following patterns:

- the years of publication ranged from 2008 to 2017, with the median year of publication being 2014;
- the most common jurisdictional focus of the KT platforms was country rather than sub-national, regional (supra-national) or global, while the most common country focus was Uganda (appearing in 13 studies) [23, 24, 33, 41–48, 52, 53], Lebanon (appearing in 8 studies, of which 6 examine Lebanon in the context of the Eastern Mediterranean region) [28–32, 34, 41, 59], and South Africa (appearing in 8 studies) [23, 24, 26, 37, 42, 49, 51, 54];
- the most common name used by the KT platforms was EVIPNet (appearing in 17 studies) [25, 28–33, 37, 41, 44–48, 50, 52, 53], whether because that was the formal name of the group or because it considered itself part of the EVIPNet ‘family’ even if it went by a different name, such as REACH Policy Initiative Uganda and its variously named rapid evidence service (e.g. Makerere University’s service, REACH Policy Initiative service, SURE project service or rapid response service);
- the most common variables and relationships addressed, were as follows:
  - ◦ descriptions (*n* = 33 of 38 studies) [23–38, 40–43, 46–49, 51–56, 58–60] were more common than formative evaluations (*n* = 18) [23, 27, 33, 36, 37, 43–47, 50, 52, 54, 55, 57–60], summative evaluations (*n* = 15) [24, 33, 36, 43, 46, 47, 50–56, 58, 59], or studies of other linkages among variables (*n* = 4) [23, 33, 36, 45];
  - ◦ of the descriptive studies, context (*n* = 24 of 33 studies) [24–26, 28–38, 40–43, 48, 49, 52–54, 56] was the most common focus, followed by activities/outputs (*n* = 17) [26, 27, 33, 36, 38, 40, 43, 46, 47, 51, 52, 54–56, 58–60] and infrastructure (*n* = 14) [23, 24, 26, 27, 33, 37, 38, 40, 46, 52, 54–56, 59];
  - ◦ of the formative evaluations, evidence briefs (*n* = 8 of 18 studies) [33, 43, 44, 47, 52, 55, 57, 59] and deliberative dialogues (*n* = 6) [33, 47, 52, 55, 57, 59] were the most common activities and outputs examined;
  - ◦ of the summative evaluations, deliberative dialogues were the most common activities examined (*n* = 4 of 15 studies) [33, 47, 55, 59];
  - ◦ of the studies of other linkages among variables, the relationships between context and activities and outputs (*n* = 3 of 4 studies) [33, 36, 45] as well as infrastructure and activities and outputs (*n* = 3) [23, 33, 45] were most commonly examined, followed by context and infrastructure (*n* = 1) [23];
- the time period studied ranged from 1996 to 2015, with the median year of time period studied (if ranges were given, the value in the middle of the range) being 2010; and
- the most common data collection methods used were interviews (*n* = 20 of 38 studies) [23, 24, 26, 27, 33–38, 42, 44–46, 49, 51, 54, 58–60], surveys (*n* = 17) [27, 28, 30–32, 34, 40, 42, 43, 46, 47, 50–52, 56, 57, 59] and case studies involving multiple methods (*n* = 4) [54, 56, 59, 60] (there were 12 articles studying only one case [35, 43–46, 48, 54, 56–60] and only 4 of them used more than one data collection method).

Turning now to the quality assessments for studies including a formative and/or summative evaluation, which we present in Additional file 4 alongside quality assessments for all included studies, we offer the following summary:

- for formative evaluations, the mean and median quality scores were 1.0 and 1.0 out of 3, respectively, and the most common criterion responsible for a lower score was a response rate lower than 60%; and
- for summative evaluations, the mean and median quality scores were 1.3 and 1.0, respectively, but this time out of 5, and the most common criterion responsible for a lower score was two or more types of competing variables examined.

### Summary of findings

Finally, we turn to the summary of findings from included studies (which we present in detail in Additional file 5). Beginning this summary with descriptions of the more frequently mentioned activities and outputs (Table 1), we found that:

- evidence briefs and deliberative dialogues were the activities/outputs that have been the most extensively studied and the most widely undertaken (both in numbers, with the exception of rapid syntheses in some countries, and in different contexts); and
- rapid evidence services and capacity-building workshops (specifically those focused on evidence use because these are the ones that seek to directly influence the use of research evidence in policy-making) were arguably the next most extensively studied and widely undertaken (if not counting ‘other evidence outputs’ that appear likely to be produced by KT platform staff but are not as central to their goal of supporting evidence-informed policy-making).

**Table 1:** Findings from descriptions of activities and outputs*

Domain	Themes	Factors influencing weight given to themes		
		Larger number of studies	Higher-quality studies	Studies in different contexts
Building demand	No studies identified	NA	NA	NA
Prioritisation and co-production	Priority-setting exercises for activities and outputs • Four studies described KT platforms conducting priority-setting exercises involving policy-makers, stakeholders and researchers but the numbers were unclear and the formats were not described in detail [26, 33, 52, 59] – *1.5/3, 2/5, 1.5/3, 2.5/5*, respectively	Yes (4)	Yes (1)	NA
Packaging, push, and support to implementation	Evidence briefs • Nine studies described KT platforms producing evidence briefs [24, 27, 33, 47, 52, 54, 55, 58, 59] – *1.5/5, 1.5/3, 2/5, 3/5, 1.5/3, 2.5/5, 1/5, 0/3, 2.5/5*, respectively ◦ Across these nine studies, 24 unique KT platforms operating in 15 unique countries produced 63 evidence briefs (with double-counting possible across studies and some studies not providing numbers) ◦ Six of the nine studies described briefs that met EVIPNet (or SURE) criteria or were produced with support from EVIPNet (or SURE)	Yes (9)	No	Yes (15)
	Other evidence outputs • Systematic reviews ◦ Four studies described KT platforms producing systematic reviews [24, 33, 38, 40], although the data from two studies include high-income countries and could not be disaggregated – *1.5/5, 2/5, 2/3, 2/3*, respectively ◦ Considering only the data from the two studies focused on LMICs, only 3 KT platforms (each in a separate country) produced systematic reviews and then only infrequently [24, 33] – *1.5/5, 2/5*, respectively • Traditional research outputs ◦ Seven studies described KT platforms producing traditional research outputs [24, 26, 27, 33, 38, 40, 54], although again the data from two studies include high-income countries and could not be disaggregated – *1.5/5, 1.5/3, 1.5/3, 2/5, 2/3, 2/3, 2.5/5*, respectively ◦ These outputs include articles in peer-reviewed journals [24, 26, 27, 33, 54] – *1.5/5, 1.5/3, 1.5/3, 2/5, 2.5/5*, respectively, research reports [24, 26, 27, 54] – *1.5/5, 1.5/3, 1.5/3, 2.5/5*, respectively, policy-relevant research in various formats [24] – *1.5/5*, and conference presentations [54] – *2.5/5*	No (2 or 5)	Yes (2 and 2)	No (3 or NA)
Facilitating pull	Online clearinghouses • Four studies described KT platforms developing online clearinghouses [33, 40, 52, 55] – *2/5, 2/3, 1.5/3, 1/5*, respectively, although the data from one study include high-income countries and could not be disaggregated [30] – *2/3* ◦ Two studies described KT platforms developing clearinghouses, with the one in Uganda (REACH Policy Initiative Uganda) focused on health policy and systems research from that country (Uganda; 2012) [52] – *1.5/3* and the one in Cameroon (EVIPNet Cameroon) focused on health policy and systems research as well as evidence briefs and syntheses (Cameroon; 2009) [52, 55] – *1.5/3, 1/5*, respectively ◦ One study described five KT platforms as being in the process of creating online clearinghouses [33] – *2/5*	No (3)	Yes (1)	No (2)
	Rapid evidence services • Four studies described KT platforms implementing rapid evidence services [33, 46, 52, 60] – *2/5, 1/5, 1.5/3, 1/3*, respectively ◦ Across these four studies, four KT platforms (each in a separate country) were operating such services, including REACH Policy Initiative Uganda, EVIPNet Burkina Faso, EVIPNet Cameroon and ZAMFOHR ◦ REACH Policy Initiative Uganda received 65 evidence requests from 30 policy-makers and stakeholders in the first 28 months, returned 82% of responses on time [46] – *1/5* and it produced 73 briefs in the 2010–2012 period [52] – *1.5/3* ◦ EVIPNet Burkina Faso delivered five rapid syntheses to four national-level policy-makers during its experimental phase (March–December 2011) [60] – *1/3* ◦ Using three of the same studies, the three named KT platforms appear to have produced 99 rapid syntheses [46, 52, 60] – *1/5, 1.5/3, 1/3*	Yes (4)	No	No (3 or 4)
	Building capacity to use (and support the use of) research evidence • Three studies described at least five KT platforms based in four different countries that conducted capacity-building workshops for policy-makers and other evidence users in the areas of using research evidence, engaging in evidence-informed policy-making and undertaking KT activities [24, 33, 55] – *1.5/5, 2/5, 1/5*, respectively ◦ Two additional studies [38, 40] – *2/3, 2/3*, respectively*,* described numerous KT platforms that conducted capacity-building workshops, but these studies include high-income countries and could not be disaggregated • Three studies described 12 KT platforms based in 11 different countries that conducted internal capacity-building workshops for KT platform staff about various KT activities and outputs [33, 54, 58] – *2/5, 2.5/5, 0/3*, respectively, with 10 focused on preparing evidence briefs [33], three focused on conducting systematic reviews and undertaking priority-setting exercises [33], and two on KT activities in general [54, 58] • Three studies described four KT platforms based in three different countries that conducted 37 capacity-building workshops for a broad range of groups – policy-makers, stakeholders and researchers – in the area of KT activities [52, 55, 56] – *1.5/3, 1/5, 2.5/3*, respectively	No for use (3)	No	No for use (4)
Exchange	Deliberative dialogues • Eight studies described KT platforms convening deliberative dialogues [24, 27, 33, 47, 52, 55, 58, 59] – *1.5/5, 1.5/3, 2/5, 3/5, 1.5/3, 1/5, 0/3, 2.5/5*, respectively ◦ Across these eight studies, 20 KT platforms in 15 different countries convened 45 deliberative dialogues (with double-counting possible across studies and some studies not providing numbers) ◦ Seven of the eight studies described dialogues that were informed by a pre-circulated evidence brief (while the other did not specify this) ◦ Six of the eight studies described dialogues that met EVIPNet (or SURE) criteria or were convened with support from EVIPNet (or SURE)	Yes (8)	Yes (1)	Yes (15)

*EVIPNet* Evidence-Informed Policy Networks, *KT* knowledge translation, *NA* not available, REACH Regional East African Community Health, SURE Supporting the Use of Research Evidence, *ZAMFOHR* Zambia Forum for Health Research

*Supporting studies for each finding are cited, and quality scores for each supporting study are presented in italicized text

The evidence briefs (sometimes called ‘evidence briefs for policy’) described in the included studies are a jurisdiction-specific summary of what is known from local data and studies and from systematic reviews about (1) a problem and its causes, (2) options for addressing it and (3) key implementation considerations. The deliberative dialogues (sometimes called ‘policy dialogues’ or ‘stakeholder dialogues’) involve a diverse group of policy-makers, stakeholders and researchers – informed by a pre-circulated evidence brief – deliberating about the same three topics as well as next steps for different constituencies, with the key themes captured in a dialogue summary. The combination of the evidence brief and dialogue summary are intended to provide policy-makers with the best available research evidence and systematically elicited stakeholder insights. The rapid evidence services described in the included studies provide a summary of what is known, typically from systematic reviews and sometimes from local data and studies, and from jurisdictional scans. These are typically in time frames of days to weeks and about one of a problem, policy options, or implementation considerations. The capacity-building workshops are intended to help policy-makers and stakeholders to find and use research evidence on their own as part of a systematic approach to examining priority issues, whether they have minutes, hours or days to inform a policy-making process. The systematic approach maps policy questions about problems, options and implementation considerations onto types of research evidence and then those types of research evidence onto appropriate sources of pre-appraised, synthesised research evidence (such as Health Systems Evidence).

Moving on to formative evaluations of activities and outputs (Table 2), we found that:

- evidence briefs and deliberative dialogues were the activities/outputs that have been the most extensively studied and the most widely viewed as helpful both in general and in terms of the specific design features commonly used by EVIPNet; and
- rapid evidence services were the next most extensively studied but only in a single jurisdiction (Uganda).

**Table 2:** Findings from formative evaluations of activities and outputs*

Domain	Themes	Factors influencing weight given to themes		
		Larger number of studies	Higher-quality studies	Studies in different contexts
Building demand	Awareness raising • One study found that increasing awareness among policy-makers, stakeholders and researchers about the importance of initiatives to support evidence-informed policy-making was regarded as an organisational strength across seven KT platforms [33] – *2/5 (formative score 1/3)*	No (1)	No	Yes (7)
Prioritisation and co-production	Priority-setting exercises for activities and outputs • One study found that prioritising operational research was consistently regarded as an organisational strength across three KT platforms, with one each operating in South Africa, Thailand and Uganda [37] – *2.5/3 (formative score 2/3)*	No (1)	Yes (1)	No (3)
Packaging, push, and support to implementation	Evidence briefs • Six studies examined EVIPNet-style evidence briefs [33, 47, 52, 55, 57, 59] – *2/5 (formative score 1/3), 3/5 (formative score 2/3), 1.5/3, 1/5, 2/3, 2.5/5*, respectively ◦ Four studies found that they are highly regarded by policy-makers and stakeholders in Bangladesh, Nigeria and Zambia [33, 47, 55, 57], with the highest-quality study finding that EVIPNet-style evidence briefs were highly rated by policy-makers and stakeholders in Burkina Faso, Cameroon, Ethiopia, Nigeria, Uganda and Zambia – both in terms of whether they achieved their objective and in terms of their key design features – regardless of country, group or issue involved [47] ◦ Two studies found that some readers struggled with them not concluding with recommendations [47, 52] while one study found that respondents’ self-reported professional roles being other than ‘policy-maker’ or ‘stakeholder’ was a significant predictor of giving a lower helpfulness score to evidence briefs not concluding with recommendations [47] ◦ One study found that the graded-entry format of briefs is viewed a very favourable element [59]	Yes (6)	Yes (2)	No (3)
Facilitating pull	Rapid evidence services • Four studies examined the rapid evidence service in Uganda [44–46, 60] – *1/5, 1.5/3, 1/3, 1/3 (formative score 0/3)*, respectively ◦ One study found that key success factors for such services include awareness of user needs (i.e. consultation with policy-makers), the opportunity for feedback from users (i.e. being a personalised service) and working within current norms and behaviours of users [46] ◦ A second study identified regular contact between policy-makers and researchers (i.e. service staff) as a key factor in the uptake of, and response to, the service [45] ◦ Two studies found that the rapid syntheses produced by these services are perceived as a desirable and user-friendly output by policy-makers and stakeholders [44, 46] ◦ One study found identified as favourable aspects of rapid syntheses their policy relevance and right time frame for production [60] ◦ Another study identified as aspects of rapid syntheses not always meeting expectations, the speed by which they were produced/delivered, their quality, the degree of contextualisation [60] and the absence of recommendations [44]	Yes (4)	No	No (1)
	Building capacity to use (and support the use of) research evidence • Two studies examined training programmes for health policy advisory committee members to improve their use of research evidence in policy-making but neither identified explicitly the key findings from a formative evaluation [27, 58] – *1.5/3 (formative score 1/3), 0/3*, respectively ◦ One study described two 5-day training workshops that included sessions focused on the role of knowledge brokers, research methodology and writing, and impact evaluation [27] ◦ A second study described both a 1-day training workshop on evidence briefs, deliberative dialogues and priority-setting, and a 3-month training programme focused on enhancing capacity for research, evidence-informed policy-making and health policy advocacy, leadership development in resource-limited areas, and health policy monitoring, evaluation and performance assessment [58]	No (2)	No	No (1 and 1)
Exchange	Deliberative dialogues • Six studies examined deliberative dialogues informed by evidence briefs and found them to be highly regarded as a tool for enhancing evidence-informed policy-making [33, 47, 52, 55, 57, 59] – *2/5 (formative score 1/3), 3/5 (formative score 2/3), 1.5/3, 1/5, 2/3, 2.5/5*, respectively ◦ The highest-quality study found that EVIPNet-style deliberative dialogues were highly rated by policy-makers and stakeholders in Burkina Faso, Cameroon, Ethiopia, Nigeria, Uganda and Zambia – both in terms of whether they achieved their objective and in terms of their key design features – regardless of country, group or issue involved [47] ◦ The same study found that participants without past research experience were more likely to associate ‘not aiming for consensus’ with a lower rating of the helpfulness of the dialogues [47]	Yes (6)	Yes (2)	No (3)
	Research-to-policy workshops • Two studies examined research-to-policy workshops and found that they were perceived to have helped promote improved/new opportunities for collaboration and networks, increased/new knowledge, policy brief writing skills, and an enhanced understanding of the importance of research and evidence-based decision-making [43, 50] – *1/3, 1/5*, respectively ◦ One study held four successive workshops focused on research evidence and its policy implications, hands-on skills and policy brief writing, and presentation of policy briefs [43] ◦ Another study found that participants in a 3-day international forum on evidence-informed policy-making (1) identified four areas for improvement – smaller programme to accommodate more time for discussions; clearer meeting objectives; further exploration of evidence-informed policy-making initiative sustainability; and inclusion of training on writing policy briefs; and (2) highlighted presentations on country experiences and impact evaluation/analysis sessions as the most enjoyable [50]	No (2)	No	No (2)

*EVIPNet* Evidence-Informed Policy Networks, *KT* knowledge translation

*Supporting studies for each finding are cited, and quality scores for each supporting study are presented in italicized text; instances where scores for individual formative evaluations differ from overall study scores are specified

These formative evaluations took the form of surveys administered to participants in deliberative dialogues, with one survey about the pre-circulated evidence brief being completed before the dialogue began and a second survey about the dialogue itself after the dialogue was completed.

Continuing on to the summative evaluations of outcomes and impacts (Table 3), we found that:

- KT platforms as a whole have been the most extensively studied in terms of both (1) impacts on policy-making processes and (2) influence on outcomes such as stronger relationships between policy-makers and researchers and raising awareness about, and building demand for, using research evidence; and
- evidence briefs and deliberative dialogues were the next most extensively studied, both in terms of their impact on policy-makers’ and stakeholders’ intentions to act on what was learned and on select policy-making processes.

**Table 3:** Findings from summative evaluations of outcomes and impact*

Domain	Themes	Factors influencing weight given to themes		
		Larger number of studies	Higher-quality studies	Studies in different contexts
Impacts on policy-making processes	KT platforms • Ten studies reported a total of 23 KT platforms conducting activities and outputs that collectively led to some direct impacts on select policy-making processes, although the number of policy-making processes influenced and the nature of these impacts were often not described [24, 33, 36, 46, 47, 51, 53–55, 59] – *1.5/5, 2/5, 1.5/5, 1/5, 3/5, 2/5, 1/4, 2.5/5, 1/5, 2.5/5*, respectively	Yes (10)	Yes (1)	Yes (23)
	Evidence briefs and deliberative dialogues • Four studies examined EVIPNet-style evidence briefs and deliberative dialogues [33, 47, 55, 59], – *2/5, 3/5, 1/5, 2.5/5*, respectively*,* and found that they: ◦ led to strong intentions to act among dialogue participants in Burkina Faso, Cameroon, Ethiopia, Nigeria, Uganda and Zambia [47] ◦ had direct impacts on select policy-making processes in Bangladesh and Cameroon (both for evidence briefs alone and the combination of briefs and dialogues) and in nine countries with active KT platforms (for the combination of briefs and dialogues) [33, 47, 55, 59] • One study found that policy briefs different from EVIPNet-style evidence briefs led to direct impacts on select policy-making processes in South Africa [54] – *2.5/5*	Yes (4)	Yes (1)	Yes (12)
	Other activities and outputs • Other studies that examined activities and outputs that led to direct impacts on select policy-making processes focused on rapid evidence services/rapid syntheses [46] – *1/5*, research publications [24, 54] – *1.5/5, 2.5/5*, respectively, participation in government meetings [24] – *1.5/5*, translation and appraisal of research findings [36] – *1.5/5*, and research-to-policy meetings [50, 51] – *1/5, 2/5*, respectively	No for any given activity/output	No	No for any given activity/output
Outcomes – More policy-relevant research evidence available	Project-outcomes evaluation (as an activity) • One study examined the influence of an activity on this outcome and found that 73% of individuals involved in project outcome evaluation in Bangladesh believed that the project increased access to research evidence [55] – *1/5* KT platforms • One study examined the influence of two KT platforms on a range of outcomes related to policy-relevant research evidence being available (e.g. more funding for monitoring and evaluation) [53] – *1/4*	No (1)	No	No (1)
Outcomes – Stronger relationships between policy-makers and researchers	KT platforms • Three studies examined the influence of KT platforms on this outcome: ◦ eight KT platforms reported strengthened relationships among policy-makers, stakeholders, researchers [33] – *2/5* ◦ one KT platform reported new spaces for deliberations on priority health policy issues having been created through a network of local and global factors and agents [52] – *1.5/3* ◦ another KT platform reported that relationships among policy-makers, stakeholders and researchers were strengthened as a result of a deliberative dialogue organised by the KT platform, with future meetings and workshops held independently to discuss implementation as an example of such strengthening [59] – *2.5/5* Project outcome evaluation (as an activity) • The same study from Bangladesh reported above found that 73% of individuals involved in project outcome evaluation believed that the project cemented relationships between policy-makers and researchers [55] – *1/5*	No (3 or 1)	No	Yes (8+ for KT platforms)
Outcomes – Greater policy-maker capacity to use research evidence	Workshops and other forms of training • Four studies examined the influence of workshops and other forms of training on this outcome: ◦ one study found that workshops honed policy brief writing skills, increased scientific knowledge and networks with researchers, and increased awareness of the importance of research and evidence-based decision-making [43] – *1/3* ◦ a second study found that an international forum with a partial focus on capacity-building led less than half of participants to report new skills as a benefit overall (46%) but one-fifth (19%) reported an intent to utilise new skills [50] – *1/5* ◦ a third study found that a workshop improved participants’ knowledge, understanding of policy-making and use of evidence [56] – *2.5/3* ◦ a fourth study found that training future policy-makers was a key contributor to their policy influence success [54] – *2.5/5* ‘Buddy’ programme that pairs policy-makers and researchers • One study found that Policy BUDDIES (Policy BUilding Demand for evidence in Decision-making through Interaction and Enhancing Skills) enhanced the capacity of subnational policy-makers to ask for, demand and use systematic review evidence (and other products of evidence syntheses) to inform policy-making [36] – *1.5/5* Advisory committee • One study found that a Health Policy Advisory Committee improved knowledge about evidence-to-policy links, KT and operationalisation of KT amongst Health Policy Advisory Committee members as well as their capacity to find and use evidence [58] – *0/3*	No for any given form	No	No for any given form
Outcomes – Other (awareness and demand)	KT platforms • Two studies examined the influence of KT platforms on other outcomes: ◦ seven KT platforms reported they have increased awareness of the importance of initiatives supporting evidence-informed policy-making [33] – *2/5* ◦ six KT platforms reported higher policy-maker demand for KT products [33] – *2/5* ◦ one KT platform reported greater awareness of and demand for KT tools amongst policy-makers, stakeholders and researchers as a result of a deliberative dialogue organised by the KT platform [59] – *2.5/5*	No (2)	No	Yes (7)

*KT* knowledge translation

*Supporting studies for each finding are cited, and quality scores for each supporting study are presented in italicized text

However, none of these summative evaluations used a pre–post design or a control group and, with the exception of the evaluations of the influence of briefs and dialogues on intentions to act [47], none of the evaluations achieved a high quality score. The scores presented in Table 3 are the overall study scores and the scores for individual summative findings are always the same or even lower (these more detailed results are available within Additional files 3 and 5).

With respect to context and infrastructure (Table 4), we found:

- many descriptions of the political system context in which the KT platforms are operating, some descriptions of their research system context and no descriptions of their health-system context;
- some descriptions of the KT platforms’ infrastructure; and
- no formative evaluations of the KT platforms’ context or infrastructure per se, just statements made in single studies about helpful aspects of the context or infrastructure (which their designs did not permit them to examine rigorously).

**Table 4:** Findings from descriptions of context and infrastructure*

Domain	Themes	Factors influencing weight given to themes		
		Larger number of studies	Higher-quality studies	Studies in different contexts
Context – Health system	No studies identified key features of the governance, financial and delivery arrangements of the health system where KT platforms are operating	NA	NA	NA
Context – Political system where KT platforms are operating	Institutions • Five studies identified that policy legacies have left policy-makers with limited capacity for finding and using research evidence in policy-making [28, 32, 35, 36, 49] – *2/3, 1/3, 0.5/3, 1.5/5, 1.5/3*, respectively, and while one study identified a willingness among policy-makers to build their capacity [49] – *1.5/3*, another study found that policy-makers rarely participate in such activities [28] – *2/3* • Four studies identified that policy-making processes have many veto points where key interests can block evidence-informed policy proposals or support competing alternatives [28, 31, 32, 49] – *2/3, 1/3, 1/3, 2.5/3,* respectively, of which two studies indicated that this can be further complicated when there is public sector corruption [28, 32] – *2/3, 1/3*, respectively • Three studies identified a lack of administrative structures supporting evidence-informed policy-making processes [31, 32, 49] – *1/3, 1/3, 2.5/3*, respectively, and three studies identified a more general lack of dedicated government budgets for research and for supporting evidence-informed policy-making, particularly at national and regional levels [32, 37, 54] – *1/3, 2.5/5, 2.5/3*, respectively Interests • Two studies identified that select stakeholders – mid-level policy-makers, donors, universities and media – were particularly important in supporting evidence use [42, 43] – *1.5/3, 1/3*, respectively Ideas • Three studies identified that policy-makers do not value research evidence as a source of ideas for policy-making [32, 37, 49] – *1/3, 2.5/3, 1.5/3,* respectively, while three other studies identified that a political climate in which research evidence is valued could support knowledge translation [26] – *1.3/3,* and influence the development and evolution of KT platforms [52, 54] – *1.5/3, 2.5/5*, respectively External factors (i.e. factors external to the health sector) • Two studies identified that the frequent turnover of top-level policy-makers hinders efforts to support evidence-informed policy-making [28, 30] – *2/3, 1/3*, respectively • One study found extremely limited media coverage of health-systems research evidence and/or systematic reviews [25] – *1/2*	Yes (4 or 5 for some) but no for rest	Yes (2 for institutions, 1 for ideas, 1 for external factors)	Yes for those with yes in number column
Context – Research system where KT platforms are operating	Evidence availability • Three studies identified small but growing production of health policy and systems research being produced [29, 34, 48] – *1/2, 1/3, 0.5/2*, respectively, particularly in the areas of delivery arrangements and implementation strategies [29, 48] or financial arrangements [48] • One study identified that research evidence is perceived as unavailable or, more specifically, to be lacking on priority topics or (when it is available) hard for policy-makers to access, poorly timed in relation to policy-making processes or not applicable to local contexts [49] – *1.5/3* Evidence synthesis capacity • Two studies identified little evidence synthesis capacity [41, 53] – *1/3, 1/4,* respectively, particularly in the area of health systems as opposed to clinical care or public health [41] Researcher engagement in KT • Four studies identified low levels of researcher engagement in supporting evidence-informed policy-making [28–30, 56] – *2/3, 1/2, 1/3, 2.5/3*, respectively Research funding agency support for KT • One study found that most funding agencies include KT in their mandate (18 or 23), but only about one-third of funding agencies prioritise KT (8 of 23) and they allocate less than 20% of their budget to KT, and that national funding agencies give greater attention to KT than international agencies [26] – *1.5/3*	Yes (4 for one) but no for rest	Yes (1 for researcher engagement) but no for rest	Yes for those with yes in number column
Infrastructure – KT platform governance arrangements	Decision-making authority • Six studies identified the variability in whether decision-making authority for the KT platform was located in government or elsewhere, with several KT platforms operating as units within ministries of health or as units subject in other ways to ministry oversight (e.g. in a government hospital), while other KT platforms operated in academic institutions, private organisations and other settings, sometimes with governing boards having varying degrees of independence and at other times having no dedicated governance mechanism [23, 24, 33, 40, 52, 60] – *1.5/3, 1.5/5, 2/5, 2/3, 1.5/3, 1/3*, respectively Networks/multi-institutional arrangements • Three studies identified variability in whether KT platform created (or identified the need to create) a formal infrastructure to convene policy-makers, stakeholders and researchers or established informal contacts with these groups [24, 55, 56] – *1.5/5, 1/5, 2.5/3*, respectively, and while one study identified that strong linkages between KT platforms and policy-makers were very important for KT activities [24] – *1.5/5*, another study identified that these linkages could introduce conflicts of interest and be considered an organisational weakness [37] – *2.5/3* • Two studies identified that KT platforms benefited significantly from the support of EVIPNet, both through south–south collaborations (e.g. focused on rapid evidence services) or north–south collaborations (e.g. EVIPNet Cameroon, REACH Policy Initiative Uganda) [52, 60] – *1.5/3, 1/3*, respectively • One study found that most KT platforms did the work themselves ‘in house’, while some commissioned work externally [38] – *2/3*	Yes (6 for one) but no for rest	Yes (1 for each)	Yes for those with yes in number column
Infrastructure – KT platform financial arrangements	Funding • Four studies identified that short-term, unpredictable or scarce ongoing funding alongside high operating costs are major barriers to KT platform activities and sustainability [23, 33, 37, 54] – *1.5/3, 2/5, 2.5/3, 2.5/5*, respectively, one study identified that financial independence facilitated effective policy engagement [24] – *1.5/5*, and one study identified that many KT platforms do not have clear fundraising strategies [23] – *1.5/3* • Three studies identified that most KT platforms received money from funding agencies, donors or government to initiate and scale up their work [23, 52, 60] – *1.5/3, 1.5/3, 1/3*, respectively • One study identified that budgets varied widely in size (e.g. US$26,000 for the Health Policy Analysis Unit in Uganda in 2008 to US$1,300,000 for the Health Strategy and Policy Institute in Vietnam in 2007) [23] – *1.5/3*, while another study found that costs were higher during early phases (awareness, experimentation and expansion phases) that were funded externally and then lower during the consolidation phase that is funded by the government [60] – *1/3*	Yes (4 for one) but no for rest	No	Yes for those with yes in number column
Infrastructure – KT platform delivery arrangements	Human resources • Four studies identified a lack of skilled human resources to draw upon as a key organisational weakness [23, 33, 37, 54] – *1.5/3, 2/5, 2.5/3, 2.5/5*, respectively, and a fifth identified that earlier successes led to increased demand from policy-makers and stakeholders, which was difficult to meet because of the lack of skilled KT platform staff [60] – *1/3* • Four studies identified high turnover among KT platform staff [23, 33, 52, 54] – *1.5/3, 2/5, 1.5/3, 2.5/5*, respectively, with one study noting that once staff develop the necessary skills they frequently move to better paid positions elsewhere [23] – *1.5/3* • Three studies identified the range in number of KT platform staff, with one being the lower end, 50 the higher end, and many with five or fewer full-time equivalent staff [23, 38, 40] – *1.5/3, 2/3, 2/3*, respectively • Two studies identified the breadth in backgrounds of KT platform staff (e.g. medical or social/population studies, research methods training, policy analysis and writing skills, and understanding of health systems and policy-making processes) [23, 46] – *1.5/3, 1/5*, respectively • One study identified the importance of KT platform leaders, particularly in facilitating links with policy-makers and stakeholders [55] – *1/5* Scope • Two studies identified variability in the scope of KT platforms, with some focusing on one or two phases of the policy-making process (e.g. Policy BUDDIES programme in Cameroon and South Africa, respectively), some focusing on specific topic areas (e.g. public health or primary care), and some supporting policy-making about clinical practice (through guidelines) or technologies (through health technology assessments) as well as policy-making about health systems [36, 40] – *1.5/5, 2/3*, respectively Phase of development • One study identified key phases in the process of institutionalising a rapid evidence service, the different needs in different phases, and how changes within (e.g. staffing) and beyond (e.g. changes in the home directorate) the KT platform can affect the institutionalisation process [60] – *1/3*	Yes (4 for two) but no for rest	Yes (3 for human resources, 1 for scope)	Yes for those with yes in number column

*EVIPNet* Evidence-Informed Policy Networks, *KT* knowledge translation, *NA* not available

*Supporting studies for each finding are cited, and quality scores for each supporting study are presented in italicized text

The key features of the political and research system context in which the KT platforms are operating (specifically those identified in four or more studies) include (1) policy-makers have limited capacity for finding and using research evidence in policy-making; (2) policy-making processes have many veto points where key interests can block evidence-informed policy proposals or support competing alternatives; and (3) there are low levels of researcher engagement in supporting evidence-informed policy-making. The key features of the KT platform’s infrastructure (again those identified in four or more studies) include (1) variability in whether decision-making authority for the KT platform was located in government or elsewhere; (2) short-term, unpredictable or scarce ongoing funding; (3) a lack of skilled human resources to draw upon; and (4) high turnover among KT platform staff. Examples of the statements made about helpful aspects of KT platforms’ context and infrastructure include (1) high-level political support is key; (2) ‘home-grown’ models can have a greater likelihood of success; (3) strong, independent advisory or governance structures are helpful; and (4) staff who are well trained and proactive are essential.

Concluding with other types of linkages among variables, we found only four studies [23, 33, 36, 45] that examined such linkages (Table 5) and they suggest that political support (context) and networks/multi-institutional arrangements (infrastructure) can influence the demand for, and supply of, activities and outputs.

**Table 5:** Findings from studies that examine other types of linkages among variables*

Domain	Themes	Factors influencing weight given to themes		
		Larger number of studies	Higher-quality studies	Studies in different contexts
Context affects: activities/outputs ➔ impacts	No studies identified	NA	NA	NA
Context affects: activities/outputs ➔ outcomes	No studies identified	NA	NA	NA
Infrastructure affects: activities/outputs ➔ impacts	No studies identified	NA	NA	NA
Infrastructure affects: activities/outputs ➔ outcomes	No studies identified	NA	NA	NA
Context ➔ infrastructure	Financial sustainability • One study found that political transitions and institutional rivalry emerged as barriers to the financial sustainability of KT platforms [23] – *1.5/3*	No (1)	No	NA
Context ➔ activities/outputs	Political support • Two studies found that political support increased awareness and the perceived legitimacy of a KT platform and thereby the demand for (and then supply of) its activities and outputs (e.g. rapid syntheses) [33, 45] – *2/5, 1/3*, respectively External factors • One study found that external factors limited the ability of one KT platform in Cameroon to link policy-makers and researchers effectively, in this case due to a poliomyelitis outbreak limiting time and resources of policy-makers [36] – *1.5/5*	No (2 or 1)	No	Yes for political support (6)
Infrastructure ➔ activities/outputs	Networks/multi-institutional arrangements • Two studies found that networks/multi-institutional arrangements involving the KT platform and governments, stakeholder organisations, research organisations (both within and outside the KT platform’s country) and/or funding agencies increased awareness of the KT platform, provided reminders to draw on it, and developed its capacity to respond with appropriate activities and outputs [33, 45] – *2/5, 1/3*, respectively • One study examining three KT platforms found that linking KT platforms with policy-makers and forming external networks with research organisations allowed KT platforms to improve capacity for research and outputs [23] – *1.5/3*	No (3)	No	Yes (10)

*KT* knowledge translation, *NA* not available

*Supporting studies for each finding are cited, and quality scores for each supporting study are presented in italicized text

## Discussion

### Principal findings

Although it remains premature to make definitive statements about whether KT platforms in general or their particular approaches are effective, let alone whether and how context and infrastructure influence whether the approaches translate into outcomes and impact, we have a large and growing volume of research evidence (38 studies) suggesting that KT platforms offer promise in supporting evidence-informed policy-making in LMICs. Our principal findings include the following:

- country was the most common jurisdictional focus of the KT platforms and EVIPNet the most common name used by them;
- descriptions (33 of 38 studies) were more common than formative evaluations (18, of which 8 were about evidence briefs and 6 about deliberative dialogues), summative evaluations (17, of which 4 were about deliberative dialogues) or studies of other linkages among variables (4);
- interviews and surveys were the most common data collection methods used (20 and 17 of 38 studies, respectively);
- quality scores were generally low for both formative and summative evaluations;
- evidence briefs and deliberative dialogues were the activities/outputs that have been the most widely undertaken (both in numbers, with the exception of rapid syntheses in some countries and in different contexts), with rapid evidence services and capacity-building workshops the next most common;
- evidence briefs and deliberative dialogues were also the activities/outputs that have been the most extensively subjected to formative evaluation and the most widely viewed as helpful both in general and in terms of the specific design features commonly used by EVIPNet, while rapid evidence services were the next most extensively studied but only in a single jurisdiction (Uganda);
- KT platforms as a whole, followed by evidence briefs and deliberative dialogues, have been the most extensively subjected to summative evaluation; however, none of these summative evaluations used a pre–post design or a control group and, with the exception of the evaluations of the influence of briefs and dialogues on intentions to act, none of the evaluations achieved a high quality score;
- the key features of the political and research system context in which the KT platforms are operating (specifically those identified in four or more studies) include (1) policy-makers have limited capacity for finding and using research evidence in policy-making; (2) policy-making processes have many veto points where key interests can block evidence-informed policy proposals or support competing alternatives; and (3) there are low levels of researcher engagement in supporting evidence-informed policy-making;
- the key features of the KT platform’s infrastructure include (1) variability in whether decision-making authority for the KT platform was located in government or elsewhere; (2) short-term, unpredictable or scarce ongoing funding; (3) a lack of skilled human resources to draw upon; and (4) high turnover among KT platform staff; and
- the four studies examining linkages among other variables found that political support (context) and networks/multi-institutional arrangements (infrastructure) can influence the demand for, and supply of, activities and outputs.

### Strengths and limitations

Our systematic review has five main strengths, as follows: (1) we conducted an exhaustive, broad-based search for both published and grey literature; (2) we used explicit criteria to set a relatively low threshold for inclusion (and one that did not privilege certain types of KT platforms, health or political system contexts, infrastructures, approaches, or measures of outcomes and impact) and provided reassurance about reliability when only one reviewer was involved in their application (e.g. ensuring we had achieved an acceptable Kappa statistic); (3) we used explicit quality criteria that reflected best practices in studies of policy-making processes (e.g. two or more data collection methods used, two or more types of evidence use examined, and two or more competing variables examined); (4) we gave greater attention to themes that emerged from multiple studies, higher-quality studies, and different contexts; and (5) we took steps to ensure that team members who are involved in operating, supporting and evaluating KT platforms were not involved in several key steps in the review (and to provide data in this review that would allow other, more fully independent researchers to re-examine the data).

There are three main weaknesses in our review, two of which have more to do with the difficulties of describing and evaluating KT platforms than they do with the design and execution of the review, namely (1) study searches were undertaken in 2015 and 2016 and the related articles search in 2017; (2) none of the summative evaluations used formal effectiveness designs, such as a randomised controlled trial (and hence we did not use traditional risk-of-bias criteria), likely in no small part because of the lack of outcome and impact measures that can reliably perform as well as of case studies focusing on untangling actual influences on policy-making that often take place over long periods of time, behind ‘closed doors’, and in other ways that make simple metrics difficult to apply; and (3) the near absence of studies of linkages among variables highlights the sample-size challenge of finding enough contexts and infrastructures supporting a common approach that can be evaluated using standardised methods.

### Findings in relation to other studies

To the best of our knowledge, this is the first systematic review of descriptions and evaluations of KT platforms in LMICs. The ever-growing number of systematic reviews undertaken to inform efforts to support evidence-informed policy-making [61–64] differ in important ways from what has been studied in the current evaluation – some examine the factors associated with the use of research evidence in policy-making, with findings from well over 100 observational studies suggesting that several key factors (such as timing/timeliness, interactions between policy-makers and researchers, and an accordance between policy-makers’ beliefs, values, and strategies, and the available research evidence) are associated with greater prospects for evidence use (and most KT platforms’ portfolio of activities and outputs address these factors directly); some examine the effectiveness of specific approaches to supporting evidence-informed policy-making, yet the two highest-quality reviews both found only a single effectiveness study (and it was focused on public health, not health systems); and many involve an ill-defined hybrid between these two, do not distinguish between policy-making about clinical, public health and health-systems topics, and do not give attention to best practices in studies of policy-making processes.

### Implications for policy and practice

We offer three implications of our review for those creating, leading or supporting KT platforms: (1) consider expanding the current, relatively limited portfolio of activities and outputs (e.g. to include citizen panels that can bring systematically elicited citizens’ values to the table alongside research evidence in the form of evidence briefs and stakeholder insights derived from deliberative dialogues, as several high-income country KT platforms are now doing); (2) consider aligning with evaluation initiatives using a common approach to outcomes and impact measurement to facilitate cross-platform learning (as EVIPNet did in its first decade); and (3) consider giving greater attention to context (especially how to adjust infrastructure and activities and outputs to respond to unique political and health system contexts) and to infrastructure (especially how to institutionalise the KT platform in ways that minimise disruptions as governments change, external funding priorities shift and charismatic leaders are drawn into new domains), particularly given that this review suggests that the prospects for robust empirical answers to these questions are limited.

While not implications arising from this review per se, this work did prompt us to reflect on where KT platforms may need to move in future, as follows (1) consider building bridges to organisations, initiatives and networks working in complementary areas such as guideline and HTA units (that also rely on existing data and evidence) but also data-analytics, modelling, implementation research and evaluation units (that are building data and evidence de novo to support a particular phase of the policy-making process); (2) consider partnering with allies both within government (such as ministerial advisory bodies and parliamentary research offices) and outside government (such as the WHO collaborating centres and WHO country offices that also support policy-making processes, and the WHO guidance panels that produce global health-systems guidance that needs country-level activities and outputs like evidence briefs and deliberative dialogues to be contextualised in order to achieve impacts); (3) consider what can be done to institutionalise the use of research evidence in policy-making processes (e.g. requiring brief descriptions of how research evidence was used to clarify a problem, frame options and identify implementation considerations, and simple checklists of which sources of pre-appraised synthesised research evidence were searched and what types of evidence were found); and (4) consider collaborating with organisations, networks and initiatives that are operating in the broader Sustainable Development Goals space but that have not had the long-term focus on supporting evidence-informed policy-making that has been a hallmark of the health sector (particularly now that the beta version of Social Systems Evidence is available as a source of pre-appraised synthesised research evidence) across all Sustainable Development Goals except the three about the environment (climate, land and water).

### Implications for future research

While we are in urgent need of a monitoring and evaluation plan 2.0 for KT platforms, our experience with the KT platform monitoring and evaluation plan 1.0 used for EVIPNet and now with this review reinforce for us how very difficult it will be to design one that is sensitive both to best practices in studies of policy-making processes and to best practices in summative evaluations (i.e. effectiveness studies). The paradox is that KT platforms attempt to hold others to account to use research evidence in their decision-making, yet they themselves work in a space that is so difficult to evaluate in robust ways.

## Conclusions

A large and growing volume of research evidence suggests that KT platforms offer promise in supporting evidence-informed policy-making in LMICs. While our review had many strengths, many of which reflect best practices in studies of policy-making processes, unfortunately, none of the summative evaluations used formal effectiveness designs, which limits our ability to make statements about ‘what works’. KT platforms should consider as next steps expanding their current, relatively limited portfolio of activities and outputs (e.g. to include citizen panels), building bridges to complementary groups (e.g. data-analytics organisations supporting the problem-clarification phase of the policy-making process), and planning for evaluations that examine ‘what works’ for ‘what types of issues’ in ‘what types of contexts’.

## Supplementary information

**Additional file 1.** MEDLINE search string.

**Additional file 2.** Citations for ‘near miss’ papers that were excluded.

**Additional file 3.** Characteristics of included studies with reference list.

**Additional file 4.** Quality assessments for included studies.

**Additional file 5.** Summary of findings from included empirical studies.

## Data Availability

All data generated or analysed during this study are included in this published article and its additional files.

## Abbreviations

KT: Knowledge translation
HTA: Health-technology assessment
LMICs: Low- and middle-income countries
EVIPNet: Evidence-Informed Policy Networks
PRISMA: Preferred Items for the Reporting of Systematic Review and Meta-Analysis

## References

[1] Shearer JC, Abelson J, Kouyaté B, Lavis JN, Walt G (2016). Why do policies change? Institutions, interests, ideas and networks in three cases of policy reform. Health Policy Plan.

[2] Kingdon JW. Agendas, alternatives, and public policies. Updated 2nd ed. New York: Pearson; 2010.

[3] Oxman AD, Lavis JN, Lewin S, Fretheim A (2009). SUPPORT Tools for evidence-informed health Policymaking (STP) 1: what is evidence-informed policymaking?. Health Res Policy Syst.

[4] Bosch-Capblanch X, Lavis JN, Lewin S (2012). Guidance for evidence-informed policies about health systems: rationale for and challenges of guidance development. PLoS Med.

[5] Lavis JN, Røttingen J-A, Bosch-Capblanch X (2012). Guidance for evidence-informed policies about health systems: linking guidance development to policy development. PLoS Med.

[6] El-Jardali F, Fadlallah R, Lavis J. A 2-day meeting to advance the field of and innovation in knowledge translation to achieve impact. Evidenceinformed Policy Network (EVIPNet). 2018.

[7] EVIPNet Europe (2017). Introduction to EVIPNet Europe: conceptual background and case studies.

[8] Hamid M, Bustamante-Manaog T, Dung TV (2005). EVIPNet: translating the spirit of Mexico. Lancet.

[9] Corkum S, Cuervo LG, Porrás A, EVIPNet Americas Secretariat (2008). EVIPNet Americas: informing policies with evidence. Lancet.

[10] Lavis JN, Ross SE, Hurley JE (2002). Examining the role of health services research in public policymaking. Milbank Q.

[11] Moat KA, Lavis JN, Abelson J (2013). How contexts and issues influence the use of policy-relevant research syntheses: a critical interpretive synthesis. Milbank Q.

[12] Lavis JN, Panisset U (2010). EVIPNet Africa's first series of policy briefs to support evidence-informed policymaking. Int J Technol Assess Health Care.

[13] Panisset U, Koehlmoos TP, Alkhatib AH (2012). Implementation research evidence uptake and use for policy-making. Health Res Policy Syst.

[14] Redman S, Turner T, Davies H (2015). The SPIRIT Action Framework: a structured approach to selecting and testing strategies to increase the use of research in policy. Soc Sci Med.

[15] Williamson A, Barker D, Green S (2019). Increasing the capacity of policy agencies to use research findings: a stepped-wedge trial. Health Res Policy Syst.

[16] Cochrane KT Strategy Working Group. Cochrane Knowledge Translation Framework April 2017: The Cochrane Collaboration; 2017. https://community.cochrane.org/sites/default/files/uploads/Cochrane%20Knowledge%20Translation%20Framework%281%29.pdf. Accessed 1 May 2020.

[17] Lavis JN. Health Systems Evidence: taxonomy of governance, financial and delivery arrangements and implementation strategies within health systems. McMaster Health Forum. 2017; https://www.mcmasterforum.org/docs/default-source/resources/16_hse_taxonomy.pdf?sfvrsn=281c55d5_6.

[18] Johnson NA, Lavis J (2010). “Overview” in procedures manual for the “evaluating knowledge-translation platforms in low- and middle-income countries” study.

[19] Liberati A, Altman DG, Tetzlaff J (2009). The PRISMA statement for reporting systematic reviews and meta-analyses of studies that evaluate healthcare interventions: explanation and elaboration. Br Med J.

[20] Norwegian Satellite of Cochrane Effective Practice and Organisation of Care (EPOC). LMIC Filters. 2012. http://epoc.cochrane.org/lmic-filters. Accessed 13 Sept 2016.

[21] Giacomini MK, Cook DJ (2000). Users’ guides to the medical literature: XXIII. Qualitative research in health care. Are the results of the study valid?. J Am Med Assoc.

[22] Lavis JN, Hammill A, Gildiner A, McDonagh RJ, Wilson MG, Ross SE, Ouimet M, Stoddart GL. A systematic review of the factors that influence the use of research evidence by public policymakers. Final report submitted to the Canadian Population Health Initiative. Hamilton: McMaster University Program in Policy Decision-Making; 2005.

[23] Bennett S, Corluka A, Doherty J, Tangcharoensathien V (2012). Approaches to developing the capacity of health policy analysis institutes: a comparative case study. Health Res Policy Syst.

[24] Bennett S, Corluka A, Doherty J (2012). Influencing policy change: the experience of health think tanks in low- and middle-income countries. Health Policy Plan.

[25] Cheung A, Lavis JN, Hamandi A, El-Jardali F, Sachs J, Sewankambo N (2011). Climate for evidence-informed health systems: a print media analysis in 44 low- and middle-income countries that host knowledge-translation platforms. Health Res Policy Syst.

[26] Cordero C, Delino R, Jeyaseelan L (2008). Funding agencies in low- and middle-income countries: Support for knowledge translation. Bull World Health Organ.

[27] Dagenais C, Some TD, Boileau-Falardeau M, McSween-Cadieux E, Ridde V (2015). Collaborative development and implementation of a knowledge brokering program to promote research use in Burkina Faso, West Africa. Glob Health Action.

[28] El-Jardali F, Ataya N, Jamal D, Jaafar M (2012). A multi-faceted approach to promote knowledge translation platforms in eastern Mediterranean countries: climate for evidence-informed policy. Health Res Policy Syst.

[29] El-Jardali F, Jamal D, Ataya N (2011). Health policy and systems research in twelve Eastern Mediterranean countries: a stocktaking of production and gaps (2000-2008). Health Res Policy Syst.

[30] El-Jardali F, Lavis JN, Ataya N, Jamal D (2012). Use of health systems and policy research evidence in the health policymaking in Eastern Mediterranean countries: views and practices of researchers. Implement Sci.

[31] El-Jardali F, Lavis JN, Ataya N, Jamal D, Ammar W, Raouf S (2012). Use of health systems evidence by policymakers in eastern Mediterranean countries: views, practices, and contextual influences. BMC Health Serv Res.

[32] El-Jardali F, Lavis JN, Jamal D, Ataya N, Dimassi H (2014). Evidence-informed health policies in Eastern Mediterranean countries: comparing views of policy makers and researchers. Evid Policy.

[33] El-Jardali F, Lavis JN, Moat K, Pantoja T, Ataya N (2014). Capturing lessons learned from evidence-to-policy initiatives through structured reflection. Health Res Policy Syst.

[34] El-Jardali F, Saleh S, Khodor R (2015). An institutional approach to support the conduct and use of health policy and systems research: The Nodal Institute in the Eastern Mediterranean Region. Health Res Policy Syst.

[35] Imani-Nasab MH, Seyedin H, Yazdizadeh B, Majdzadeh R (2017). A qualitative assessment of the evidence utilization for health policy-making on the basis of SUPPORT tools in a developing country. Int J Health Policy Manag.

[36] Langlois EV, Montekio VB, Young T, Song K, Alcalde-Rabanal J, Tran N (2016). Enhancing evidence informed policymaking in complex health systems: lessons from multi-site collaborative approaches. Health Res Policy Syst.

[37] Lavis JN, Moynihan R, Oxman AD, Paulsen EJ (2008). Evidence-informed health policy 4 - case descriptions of organizations that support the use of research evidence. Implement Sci.

[38] Lavis JN, Oxman AD, Moynihan R, Paulsen EJ (2008). Evidence-informed health policy 3 - interviews with the directors of organizations that support the use of research evidence. Implement Sci.

[39] Lavis JN, Oxman AD, Moynihan R, Paulsen EJ (2008). Evidence-informed health policy 1 - synthesis of findings from a multi-method study of organizations that support the use of research evidence. Implement Sci.

[40] Lavis JN, Paulsen EJ, Oxman AD, Moynihan R (2008). Evidence-informed health policy 2 - survey of organizations that support the use of research evidence. Implement Sci.

[41] Law T, Lavis JN, Hamandi A, Cheung A, El-Jardali F (2012). Climate for evidence-informed health systems: a profile of systematic review production in 41 low- and middle-income countries, 1996-2008. J Health Serv Res Policy.

[42] Makan A, Fekadu A, Murhar V (2015). Stakeholder analysis of the Programme for Improving Mental health carE (PRIME): baseline findings. Int J Ment Health Syst.

[43] Mbonye AK, Magnussen P (2013). Translating health research evidence into policy and practice in Uganda. Malar J.

[44] Mijumbi-Deve R, Rosenbaum SE, Oxman AD, Lavis JN, Sewankambo NK (2017). Policymaker experiences with rapid response briefs to address health-system and technology questions in Uganda. Health Res Policy Syst.

[45] Mijumbi-Deve R, Sewankambo NK (2017). A process evaluation to assess contextual factors associated with the uptake of a rapid response service to support health systems’ decision-making in Uganda. Int J Health Policy Manag.

[46] Mijumbi-Deve RM, Oxman AD, Panisset U, Sewankambo NK (2014). Feasibility of a rapid response mechanism to meet policymakers’ urgent needs for research evidence about health systems in a low income country: a case study. Implement Sci.

[47] Moat KA, Lavis JN, Clancy SJ, El-Jardali F, Pantoja T, Knowledge Translation Platform Evaluation Study Team (2014). Evidence briefs and deliberative dialogues: perceptions and intentions to act on what was learnt. Bull World Health Organ.

[48] Mutatina B, Basaza R, Obuku E, Lavis JN, Sewankambo N (2017). Identifying and characterising health policy and system-relevant documents in Uganda: a scoping review to develop a framework for the development of a one-stop shop. Health Res Policy Syst.

[49] Naude CE, Zani B, Ongolo-Zogo P (2015). Research evidence and policy: qualitative study in selected provinces in South Africa and Cameroon. Implement Sci.

[50] Neves J, Lavis JN, Panisset U, Klint MH (2014). Evaluation of the international forum on evidence informed health policymaking: Addis Ababa. Health Res Policy Syst.

[51] Norton TC, Howell C, Reynolds C (2016). Exploratory study of the role of knowledge brokers in translating knowledge to action following global maternal and newborn health technical meetings. Public Health.

[52] Ongolo-Zogo P, Lavis JN, Tomson G, Sewankambo NK (2014). Initiatives supporting evidence informed health system policymaking in Cameroon and Uganda: a comparative historical case study. BMC Health Serv Res.

[53] Ongolo-Zogo P, Lavis JN, Tomson G, Sewankambo NK (2015). Climate for evidence informed health system policymaking in Cameroon and Uganda before and after the introduction of knowledge translation platforms: a structured review of governmental policy documents. Health Res Policy Syst.

[54] Rispel LC, Doherty J (2011). Research in support of health systems transformation in South Africa: the experience of the Centre for Health Policy. J Public Health Policy.

[55] Shroff Z, Aulakh B, Gilson L, Agyepong IA, El-Jardali F, Ghaffar A (2015). Incorporating research evidence into decision-making processes: researcher and decision-maker perceptions from five low- and middle-income countries. Health Res Policy Syst.

[56] Uneke CJ, Ezeoha AE, Ndukwe CD, Oyibo PG, Onwe F (2012). Promotion of evidence-informed health policymaking in Nigeria: bridging the gap between researchers and policymakers. Glob Public Health.

[57] Uneke CJ, Ezeoha AE, Uro-Chukwu H (2015). Promoting evidence to policy link on the control of infectious diseases of poverty in Nigeria: outcome of a multi-stakeholders policy dialogue. Health Prom Perspect.

[58] Uneke CJ, Ndukwe CD, Ezeoha AA, Uro-Chukwu HC, Ezeonu CT (2015). Implementation of a health policy advisory committee as a knowledge translation platform: the Nigeria experience. Int J Health Policy Manag.

[59] Yehia F, El-Jardali F (2015). Applying knowledge translation tools to inform policy: the case of mental health in Lebanon. Health Res Policy Syst.

[60] Zida A, Lavis JN, Sewankambo NK, Kouyate B, Ouedraogo S (2017). Evaluating the process and extent of institutionalization: a case study of a rapid response unit for health policy in Burkina Faso. Int J Health Policy Manag.

[61] Liverani M, Hawkins B, Parkhurst JO (2013). Political and institutional influences on the use of evidence in public health policy. A systematic review. PLoS One.

[62] Mitton C, Adair CE, McKenzie E, Patten SB, Waye Perry B (2007). Knowledge transfer and exchange: review and synthesis of the literature. Milbank Q.

[63] Murthy L, Shepperd S, Clarke MJ (2012). Interventions to improve the use of systematic reviews in decision-making by health system managers, policy makers and clinicians. Cochrane Database Syst Rev.

[64] Perrier L, Mrklas K, Lavis JN, Straus SE (2011). Interventions encouraging the use of systematic reviews by health policymakers and managers: a systematic review. Implement Sci.

