# Supplementary material for: Lessons learned from descriptions and evaluations of knowledge translation platforms supporting evidence-informed policy-making in low- and middle-income countries: a systematic review
Source: Health Res Policy Syst. 2020 Oct 31;18:127. doi: 10.1186/s12961-020-00626-5 (PMC7603785; doi:10.1186/s12961-020-00626-5)
Supplement: Supplementary file 2 — Additional file 2. Citations for ‘near miss’ papers that were excluded. [file 12961_2020_626_MOESM2_ESM.docx]

**Additional file 2: Citations for ‘near miss’ papers that were excluded**

**Stage 1: Articles excluded by one reviewer (n=41)**

- Ashford LS, Smith RR, De Souza RM, Fikree FF, Yinger NV. Creating windows of opportunity for policy change: incorporating evidence into decentralised planning in Kenya. *Bull World Health Organ.* 2006;84(8):669-672.
- Barreto JO, Souza NM. [Making progress in the use of health policies and practices informed by evidence: the Piripiri-Piaui experience]. *Ciencia & Saude Coletiva.* 2013;18(1):25-34.
- Corluka A, Cohen M, Lanktree E, Larocque R. Uptake and impact of research for evidence-based practice: lessons from the Africa Health Systems Initiative Support to African Research Partnerships. *BMC Health Serv Res.* 2014;14:I1.
- Dobbins M, Robeson P, Ciliska D, et al. A description of a knowledge broker role implemented as part of a randomized controlled trial evaluating three knowledge translation strategies. *Implement Sci.* 2009;4:9.
- Ekirapa-Kiracho E, Walugembe DR, Tetui M, et al. Evaluation of a health systems knowledge translation network for Africa (KTNET): a study protocol. *Implement Sci.* 2014;9:170.
- El-Jardali F, Fadlallah R. A call for a backward design to knowledge translation. *Int J Health Policy Manag.* 2015;4(1):1-5.
- El-Jardali F, Makhoul J, Jamal D, Ranson MK, Kronfol NM, Tchaghchagian V. Eliciting policymakers' and stakeholders' opinions to help shape health system research priorities in the Middle East and North Africa region. *Health Policy Plan.* 2010;25(1):15-27.
- Ellen ME, Leon G, Bouchard G, Ouimet M, Grimshaw JM, Lavis JN. Barriers, facilitators and views about next steps to implementing supports for evidence-informed decision-making in health systems: a qualitative study. *Implement Sci.* 2014;9:179.
- Gholami J, Ahghari S, Motevalian A, et al. Knowledge translation in Iranian universities: need for serious interventions. *Health Res Policy Syst.* 2013;11:43.
- Godfrey L, Funke N, Mbizvo C. Bridging the science-policy interface: A new era for South African research and the role of knowledge brokering. *South African Journal of Science.* 2010;106(5-6):34-41.
- Goyet S, Touch S, Ir P, et al. Gaps between research and public health priorities in low income countries: evidence from a systematic literature review focused on Cambodia. *Implement Sci.* 2015;10.
- Guindon GE, Lavis JN, Becerra-Posada F, et al. Bridging the gaps between research, policy and practice in low- and middle-income countries: a survey of health care providers. *Can Med Assoc J.* 2010;182(9):E362-E372.
- Hawkes S, B KA, Jadeja N, et al. Strengthening capacity to apply health research evidence in policy making: experience from four countries. *Health Policy Plan.* 2016;31(2):161-170.
- Kammen Jv, Savigny Dd, Sewankambo N. Using knowledge brokering to promote evidence-based policy-making: the need for support structures. *Bull World Health Organ.* 2006;84(8):608-612.
- Kasonde JM, Campbell S. Creating a Knowledge Translation Platform: nine lessons from the Zambia Forum for Health Research. *Health Res Policy Syst.* 2012;10:31.
- Lavis JN, Guindon GE, Cameron D, et al. Bridging the gaps between research, policy and practice in low- and middle-income countries: a survey of researchers. *Can Med Assoc J.* 2010;182(9):E350-361.
- Le G, Mirzoev T, Orgill M, et al. A new methodology for assessing health policy and systems research and analysis capacity in African universities. *Health Res Policy Syst.* 2014;12:59.
- Majdzadeh R, Nedjat S, Fotouhi A, Malekafzali H. Iran's Approach to Knowledge Translation. *Iran J Public Health.* 2009;38:58-62.
- Mavoa H, Waqa G, Moodie M, et al. Knowledge exchange in the Pacific: The TROPIC (Translational Research into Obesity Prevention Policies for Communities) project. *BMC Public Health.* 2012;12:552.
- Mekwa NJ, Niekerk Av, Madela-Mntla EN, Jeenah M, Loots G, Mayosi BM. The development of a National Health Research Observatory in South Africa: considerations and challenges. *South African Health Review.* 2016;2016(1):235-241.
- Moat KA, Lavis JN, Abelson J. How Contexts and Issues Influence the Use of Policy-Relevant Research Syntheses: A Critical Interpretive Synthesis. *Milbank Q.* 2013;91(3):604-648.
- Nabyonga-Orem J, Dovlo D, Kwamie A, Nadege A, Guangya W, Kirigia JM. Policy dialogue to improve health outcomes in low income countries: what are the issues and way forward? *BMC Health Serv Res.* 2016;16:217.
- Oliver S, Bangpan M, Stansfield C, Stewart R. Capacity for conducting systematic reviews in low- and middle-income countries: A rapid appraisal. *Health Res Policy Syst.* 2015;13(23).
- Onwujekwe O, Uguru N, Russo G, et al. Role and use of evidence in policymaking: an analysis of case studies from the health sector in Nigeria. *Health Res Policy Syst.* 2015;13(46).
- Pariyo G, Serwadda D, Sewankambo NK, Groves S, Bollinger RC, Peters DH. A grander challenge: The case of how Makerere University College of Health Sciences (MakCHS) contributes to health outcomes in Africa. *BMC Int Health Hum Rights.* 2011;11.
- Pin NT, Muthuswamy B, Michael F, Richard H, Sofia AM, Cheong LS. Research and policy to achieve healthy aging in Asia: Recommendations from an expert workshop. *Open Longevity Science.* 2013;7:1-10.
- Shooshtari S. Factors that Facilitate and Impede Effective Knowledge Translation in Population Health Promotion: Results from a Consultation Workshop in Iran. *Health Promotion Perspectives.* 2012;2(2):126-135.
- Shooshtari S, Samadi SA, Zarei K, Naghipur S, Martin T, Lee M. Facilitating and Impeding Factors for Knowledge Translation in Intellectual and Developmental Disabilities: Results from a Consultation Workshop in Iran. *Journal of Policy and Practice in Intellectual Disabilities.* 2014;11(3):210-216.
- Struyk RJ, Damon M, Haddaway SR. Evaluating Capacity Building for Policy Research Organizations. *American Journal of Evaluation.* 2010;32(1):50-69.
- Struyk RJ, Haddaway SR. Mentoring Policy Research Organizations: Project Evaluation Results. *Voluntas.* 2012;23(3):636-660.
- Uneke CJ, Aulakh BK, Ezeoha AE, Ndukwe CD, Onwe F. Bridging the divide between research and policy in Nigeria: The role of a health policy advisory committee. *J Public Health Policy.* 2012;33(4):423-429.
- Uneke CJ, Ezeoha AE, Ndukwe CD, Oyibo PG, Onwe F. Development of health policy and systems research in Nigeria: lessons for developing countries' evidence-based health policy making process and practice. *Healthcare Policy.* 2010;6(1):e109-126.
- Uneke CJ, Ezeoha AE, Ndukwe CD, et al. Individual and organisational capacity for evidence use in policy making in Nigeria: an exploratory study of the perceptions of Nigeria health policy makers. *Evid Policy.* 2011;7(3):251-276.
- Uneke CJ, Ezeoha AE, Uro-Chukwu H, et al. Improving Nigerian health policymakers' capacity to access and utilize policy relevant evidence: Outcome of information and communication technology training workshop. *Pan Afr Med J.* 2015;21.
- Utrobicic A, Chaudhry N, Ghaffar A, Marusic A. Bridging knowledge translation gap in health in developing countries: visibility, impact and publishing standards in journals from the Eastern Mediterranean. *BMC Med Res Methodol.* 2012;12:66.
- Vargas E, Becerril-Montekio V, Gonzalez-Block MA, et al. Mapping the use of research to support strategies tackling maternal and child health inequities: evidence from six countries in Africa and Latin America. *Health Res Policy Syst.* 2016;14.
- Wang J, Jin X. Translating evidence into policy in China: opportunities and challenges. *Fronteras en Medicina.* 2011;5(3):315-320.
- Waqa G, Mavoa H, Snowdon W, et al. Participants' perceptions of a knowledge-brokering strategy to facilitate evidence-informed policy-making in Fiji. *BMC Public Health.* 2013;13:725.
- Waqa G, Mavoa H, Snowdon W, et al. Knowledge brokering between researchers and policymakers in Fiji to develop policies to reduce obesity: a process evaluation. *Implement Sci.* 2013;8:74.
- Yazdizadeh B, Majdzadeh R, Janani L, et al. An assessment of health research impact in Iran. *Health Res Policy Syst.* 2016;14.
- Young T, Garner P, Clarke M, Volmink J. Evidence-based health care and policy in Africa: Past, present, and future. *J Clin Epidemiol.* 2016;83.

**Stage 2: Articles excluded by two reviewers (n=28)**

- Akintola O, Lavis JN, Hoskins R. Print media coverage of primary healthcare and related research evidence in South Africa. *Health Res Policy Syst.* 2015;13.
- Ayah R, Jessani N, Mafuta EM. Institutional capacity for health systems research in East and Central African schools of public health: knowledge translation and effective communication. *Health Res Policy Syst.* 2014;12:20.
- Corluka A, Hyder AA, Segura E, Winch P, McLean RKD. Survey of Argentine health researchers on the use of evidence in policymaking. *PLoS One.* 2015;10(4).
- Corluka A, Hyder AA, Winch PJ, Segura E. Exploring health researchers' perceptions of policymaking in Argentina: a qualitative study. *Health Policy Plan.* 2014;29:ii40-49.
- Dias RI, Barreto JO, Souza NM. [Current status of the Evidence-Informed Policy Network (EVIPNet) in Brazil: case report]. *Pan American Journal of Public Health.* 2014;36(1):50-56.
- El-Jardali F, Bou Karroum L, Bawab L, et al. Health Reporting in Print Media in Lebanon: Evidence, Quality and Role in Informing Policymaking. *PLoS One.* 2015;10(8):e0136435.
- Ellen ME, Lavis JN, Sharon A, Shemer J. Health systems and policy research evidence in health policy making in Israel: what are researchers' practices in transferring knowledge to policy makers? *Health Res Policy Syst.* 2014;12:10.
- Guerra G, Borde E, Snyder VNSd. Measuring health inequities in low and middle income countries for the development of observatories on inequities and social determinants of health. *International Journal for Equity in Health.* 2016;15(9).
- Hyder AA, Corluka A, Winch PJ, et al. National policy-makers speak out: are researchers giving them what they need? *Health Policy Plan.* 2011;26(1):73-82.
- Jessani N, Kennedy C, Bennett S. The human capital of knowledge brokers: an analysis of attributes, capacities and skills of academic teaching and research faculty at Kenyan schools of public health. *Health Res Policy Syst.* 2016;14.
- Jessani NS, Boulay MG, Bennett SC. Do academic knowledge brokers exist? Using social network analysis to explore academic research-to-policy networks from six schools of public health in Kenya. *Health Policy Plan.* 2016;31(5):600-611.
- Koon AD, Rao KD, Tran NT, Ghaffar A. Embedding health policy and systems research into decision-making processes in low- and middle-income countries. *Health Res Policy Syst.* 2013;11(1).
- Majdzadeh R, Nedjat S, Denis JL, Yazdizadeh B, Gholami J. 'Linking research to action' in Iran: two decades after integration of the Health Ministry and the medical universities. *Public Health.* 2010;124(7):404-411.
- Majdzadeh R, Sadighi J, Nejat S, Mahani AS, Gholami J. Knowledge translation for research utilization: design of a knowledge translation model at Tehran University of Medical Sciences. *J Contin Educ Health Prof.* 2008;28(4):270-277.
- Mbachu CO, Onwujekwe O, Chikezie I, Ezumah N, Mahua D, Uzochukwu BSC. Analysing key influences over actors' use of evidence in developing policies and strategies in Nigeria: a retrospective study of the Integrated Maternal Newborn and Child Health strategy. *Health Res Policy Syst.* 2016;14.
- Nabyonga Orem J, Mafigiri DK, Marchal B, Ssengooba F, Macq J, Criel B. Research, evidence and policymaking: the perspectives of policy actors on improving uptake of evidence in health policy development and implementation in Uganda. *BMC Public Health.* 2012;12:109.
- Nabyonga Orem J, Mafigiri DK, Nabudere H, Criel B. Improving knowledge translation in Uganda: more needs to be done. *Pan Afr Med J.* 2014;17:14.
- Nabyonga Orem J, Marchal B, Mafigiri D, et al. Perspectives on the role of stakeholders in knowledge translation in health policy development in Uganda. *BMC Health Serv Res.* 2013;13.
- Nabyonga-Orem J, Mijumbi R. Evidence for informing health policy development in Low-income Countries (LICs): perspectives of policy actors in Uganda. *Int J Health Policy Manag.* 2015;4(5):285-293.
- Nabyonga-Orem J, Ssengooba F, Mijumbi R, Tashobya CK, Marchal B, Criel B. Uptake of evidence in policy development: the case of user fees for health care in public health facilities in Uganda. *BMC Health Serv Res.* 2014;14:639.
- Nutley T, Gnassou L, Traore M, Bosso AE, Mullen S. Moving data off the shelf and into action: an intervention to improve data-informed decision making in Cote d'Ivoire. *Global Health Action.* 2014;7:25035.
- Pooransingh S, Misir A, Ramdath D, et al. Barriers and facilitators to establishing a national public health observatory. *Pan American Journal of Public Health.* 2015;38(5):403-409.
- Rizk A, Kronfol NM, Moffatt S, Zaman S, Fares S, Sibai AM. A survey of knowledge-to-action pathways of aging policies and programs in the Arab region: the role of institutional arrangements. *Implement Sci.* 2015;10:170.
- Shearer JC, Abelson J, Kouyate B, Lavis JN, Walt G. Why do policies change? Institutions, interests, ideas and networks in three cases of policy reform. *Health Policy Plan.* 2016;31(9):1200-1211.
- Shearer JC, Dion M, Lavis JN. Exchanging and using research evidence in health policy networks: a statistical network analysis. *Implement Sci.* 2014;9:126.
- Uzochukwu B, Mbachu C, Onwujekwe O, et al. Health policy and systems research and analysis in Nigeria: Examining health policymakers' and researchers' capacity assets, needs and perspectives in south-east Nigeria. *Health Res Policy Syst.* 2016;14.
- Walugembe DR, Kiwanuka SN, Matovu JKB, Rutebemberwa E, Reichenbach L. Utilization of research findings for health policy making and practice: Evidence from three case studies in Bangladesh. *Health Res Policy Syst.* 2015;13.
- Yazdizadeh B, Majdzadeh R, Alami A, Amrolalaei S. How can we establish more successful knowledge networks in developing countries? Lessons learnt from knowledge networks in Iran. *Health Res Policy Syst.* 2014;12:63.
