# Supplementary material for: Lessons learned from descriptions and evaluations of knowledge translation platforms supporting evidence-informed policy-making in low- and middle-income countries: a systematic review
Source: Health Res Policy Syst. 2020 Oct 31;18:127. doi: 10.1186/s12961-020-00626-5 (PMC7603785; doi:10.1186/s12961-020-00626-5)
Supplement: Supplementary file 3 — Additional file 3. Characteristics of included studies with reference list. [file 12961_2020_626_MOESM3_ESM.docx]

**Additional file 3: Characteristics of included studies with reference list*^[[1]](#footnote-1),^^[[2]](#footnote-2)^***

| **Lead**  **author, year, citation** | **Jurisdictional focus of KT platform(s)** | **KT platform(s) addressed in study** | **Time period studied** | **Last year data were collected** | **Objective(s)** | **Data-collection methods used** | **Types of evaluations included in study** |
| --- | --- | --- | --- | --- | --- | --- | --- |
| Bennett, Corluka, 2012 [1] | Multi-national | - Health Strategy and Policy Institute (HSPI), Vietnam; - Health Economics Unit (HEU), South Africa; - Institute for Health Systems (IHS), India; - Health Economics Institute (HEI), Bangladesh; - Health Policy Analysis Unit (HPAU), Uganda; - Centre for Health and Social Services (CHeSS), Ghana | 2009 | 2009 | To assess the factors that facilitate health policy analysis institutes (HPAIs) in LMICs, and understand the nature of support for capacity development for these institutes through analysis of comparative case studies of six HPAIs | Interviews  Document review | 1b, 2b, 4a, 4c |
| Bennett, Corluka, 2012 [2] | Multi-national | - HSPI, Vietnam; - HEU, South Africa; - IHS, India; - HEI, Bangladesh; - HPAU, Uganda; - CHeSS, Ghana | 2009-2010 | 2010 | To study the contributions of HPAIs to health policy agenda setting, formulation, implementation, and monitoring and evaluation processes in LMICs, and to assess facilitating factors (organizational form/structure) for HPAIs contributing positively to health policy in LMICs | Interviews  Document review  Financial information | 1a, 1b, 1c, 3b |
| Cheung, Lavis, 2011 [3] | Global | - EVIPNet and other KT platforms | 2007 | NR; search conducted for the year 2007 | To examine whether and how policymakers, stakeholders, and researchers discuss health policy priorities, research evidence, and health policy dialogues in the print media in order to assess climate for evidence-informed health systems and provide a baseline for KT platform evaluation | Media analysis | 1a |
| Cordero, Delino, 2008 [4] | Global | - 23 health research funding agencies operating in part as KT platforms | 2003-2004 | 2004 | To describe how health research funding agencies support knowledge translation through their funded research into policy in LMICs | Interviews  Document review | 1a, 1b, 1c |
| Dagenais, Some, 2015 [5] | National | - KB program in district of Kaya, Burkina Faso | 2011-2013 | 2013 | To present the collaborative development of a knowledge brokering (KB) strategy implemented in Burkina Faso, and evaluate its implementation at year 1 | Questionnaires  Interviews | 1b, 1c, 2c |
| El-Jardali, Ataya, 2012 [6] | Regional | - EVIPNet EMR | 2010 | 2010 | To assess the climate for evidence-informed policy in the Eastern Mediterranean Region (EMR), explore current processes and weaknesses of health policymaking, identify priorities (e.g., policy brief short-term requirements) and country-specific requirements for establishing KTPs | Questionnaires | 1a |
| El-Jardali, Jamal, 2011 [7] | Regional | - EVIPNet EMR | 2000-2008 | 2008 | To profile research production and output on health policy and systems research (HPSR), by identifying publications between 2000 and 2008 in 12 countries in the Eastern Mediterranean Region, identify gaps in production, and assess whether existing HPSR addresses regional priorities around financing, human resources, and the non-state sector in health | Documentary analysis | 1a |
| El-Jardali, Lavis, 2014 [8] | Global | - Evidence to Policy (E2P) Argentina; - E2P Bangladesh; - E2P Nigeria; - EVIPNet Burkina Faso; - EVIPNet Cameroon; - EVIPNet Central African Republic; - EVIPNet Ethiopia; - REACH-PI, Uganda; - KTP Sudan; - ZAMFOHR | 2012 | 2012 | To gain a better understanding of knowledge translation platforms (KTPs) in low and middle-income countries (LMICs) by examining i) activities conducted by KTPs, ii) the way in which these activities and their outputs are perceived by KTP leaders, policymakers, and stakeholders, iii) factors supporting and challenges impeding KTP work (and lessons learned), and, iv) factors ensuring KTP sustainability | Interviews  Document review  Observation of deliberations | 1a, 1b, 1c, 2c, 3a, 3b, 4b, 4c |
| El-Jardali, Lavis, 2012 [9] | Regional | - EVIPNet EMR | 2000-2008 | NR; study published in 2012 | To explore the views and experiences of researchers regarding the role of health policy and systems evidence in policymaking in 12 Eastern Mediterranean countries (i.e., facilitators, barriers, influencing factors of evidence in policymaking) | Surveys | 1a |
| El-Jardali, Lavis, 2012 [10] | Regional | - EVIPNet EMR; - Middle East and North Africa (MENA) Health Policy Forum | 2010 | 2010 | To explore policymakers views and experiences regarding the use of health systems evidence in policymaking in 10 Eastern Mediterranean countries (i.e., facilitators, barriers, influencing factors of evidence in policymaking) | Surveys | 1a |
| El-Jardali, Lavis, 2014 [11] | Regional | - EVIPNet EMR; - Middle East and North Africa (MENA) Health Policy Forum | NR | NR; study published in 2014 | To conduct comparative analysis (via data from two separate surveys) about policymakers and researchers views and practices, and the use of health systems evidence in policymaking within EMR countries | Surveys | 1a |
| El-Jardali, Saleh, 2015 [12] | Regional | - Nodal Institute (Lebanon); - 3 Sub-Nodes (Bahrain, Jordan, Tunisia) | 2013-2014 | 2014 | To offer a structured reflection on establishment and functioning of the EMR HPSR Nodal Institute and three sub-nodes, focusing on approaches used to support HPSR, activities conducted, methods used to apply these activities, and outcomes of these activities | Questionnaires  Interviews | 1a |
| Imani-Nasab, Seyedin, 2017 [13] | National | - National Parliament research center; establishment of the Council of Health Policy in the Iranian MoHME; - National Institute for Health Research and Health Technology Assessment Bureau in MOHME | NR | NR; study published in 2017 | To qualitatively describe the process of evidence utilization for developing policy documents in the Iranian Ministry of Health and Medical Education (MoHME) and compare these findings with the process recommended by SUPPORT tools, in order to understand evidence-informed policymaking and the feasibility of these tools in a developing country | Interviews | 1a |
| Langlois, Montekio, 2016 [14] | Multi-national | - *Intervention:*   Policy BUDDIES program operating in two countries, embedded within a broader initiative sponsored by the Alliance for Health Policy & Systems Research programme of work entitled “Leadership Development for Enhanced Decision Making” | 2013-2015 | 2015 | To reflect upon the implementation and impact of two multi-site evidence-to-policy interventions (based on iterative exchanges between researchers and policymakers) in LMICs using a mixed-method approach; these interventions were implemented by the Alliance for Health Policy & System Research programme of work entitled “Leadership Development for Enhanced Decision Making” | Interviews  Document Review  Focus groups | 1a, 1c, 2c, 3a, 3b, 4b |
| Lavis, Oxman, 2008 [15] | Global | *See [16-18] for details about the three sub-studies.* | NR | NR; study published in 2008 | To identify organizations around the world (specifically in LMICs) that successfully or innovatively support the use of research evidence in developing clinical practice guidelines (CPGs), health technology assessments (HTAs), and health policies; to describe the experiences of these organizations | NA (no data collection method used; synthesis of findings from [16-18]) | *See [16-18] for details about the three sub-studies.* |
| Lavis, Paulsen, 2008 [16] | Global | - 24 GSUs (from a total of 44 total organizations in LMICs studied) | NR | NR; study published in 2008 | To identify organizations around the world (specifically in LMICs) that successfully or innovatively support the use of research evidence in developing clinical practice guidelines (CPGs), health technology assessments (HTAs), and health policies; to describe the experiences of these organizations | Surveys | 1a, 1b, 1c |
| Lavis, Oxman, 2008 [17] | Global | - 12 GSUs (from a total of 25 organizations included) | NR | NR; study published in 2008 | To identify organizations around the world (specifically in LMICs) that successfully or innovatively support the use of research evidence in developing clinical practice guidelines (CPGs), health technology assessments (HTAs), and health policies; to describe the experiences of these organizations | Interviews | 1a, 1b, 1c |
| Lavis, Moynihan, 2008 [18] | Global | - REACH-PI; - Thailand research units; - Free State, South Africa researcher-policymaker partnership; - Mexico Seguro Popular initiative | NR | NR; study published in 2008 | To identify organizations around the world (specifically in LMICs) that successfully or innovatively support the use of research evidence in developing clinical practice guidelines (CPGs), health technology assessments (HTAs), and health policies; to describe the experiences of these organizations | Interviews  Document review | 1a, 1b, 2b, 2c |
| Law, Lavis, 2012 [19] | Global | - EVIPNet and other KT platforms | 1996-2008 | NR; search conducted for the period 1996-2008 | To profile published systematic review production between 1996 and 2008 that had authors based in 41 countries (or targeted these countries) that host KT facilities in order to assess climate for evidence-informed health systems | Documentary analysis | 1a |
| Makan, Fekadu, 2015 [20] | Multi-national | - PRIME | NR | NR; study published in 2015 | To conduct stakeholder analyses in 5 countries participating in the PRogramme for Improving Mental health carE (PRIME), evaluate a template for cross-country comparison of the analyses, and assess the utility of stakeholder analysis in identifying and characterizing support for various actions in mental health policy and systems research | Interviews  Focus group discussion assessments  Survey | 1a |
| Mbonye and Magnussen, 2013 [21] | National | - Uganda National Health Research Organization (UNHRO) | 2006-2009 | 2009 | To present the results and lessons learned of four annual research-to-policy workshops involving researchers, policymakers, civil society, and media | Semi-structured questionnaires | 1a, 1c, 2c, 3a |
| Mijumbi, Oxman, 2014 [22] | National | - REACH-PI (rapid response service) | March 2010-July 2012 | 2012 | To assess the feasibility of a rapid response mechanism in Uganda, with the goal of elucidating its performance in meeting policymakers’ needs for health systems research evidence in a timely and relevant manner | Questionnaires  Interviews (not reported in the results) | 1b, 1c, 2c, 3b |
| Mijumbi-Deve, Rosenbaum, 2017 [23] | National | - REACH-PI (partnered with WHO’s EVIPNet and SURE project) | 2010-2013 | 2013 | To explore the experiences of Ugandan policymakers with a rapid response service brief template, use this feedback to improve the template format, and evaluate how well revised brief templates met policymaker needs | Interviews (for user testing) | 2c |
| Mijumbi-Deve and Sewankambo, 2017 [24] | National | - REACH-PI (partnered with WHO’s EVIPNet and SURE project) - RRS at the College of Health Sciences in Makerere University (formerly called REACH-PI service, SURE project service, Makerere University’s service, and the RRS) | March 2010-May 2014 | 2014 | To explore contextual factors associated with how and why a rapid response service (RRS) may be taken up by users in Uganda and understand how these factors impact implementation and scale-up of RRSs in similar settings (following piloting an RRS beginning in 2010), through a case study using process evaluation methods | In-depth interviews | 2c, 4b, 4c |
| Moat, Lavis, 2014 [25] | Regional | - EVIPNet and other KT platforms | 2009 | 2010 | To evaluate the usefulness of and assess views on evidence briefs and deliberative dialogues in six African nations | Surveys | 1c, 2c, 3b |
| Mutatina, Basaza, 2017 [26] | National | - REACH-PI (specifically it’s clearinghouse service entitled the Uganda Clearinghouse for Health Policy and Systems) | January 2000-December 2014 | 2014 | To conduct a scoping review of relevant Uganda-specific health policy and systems documents produced over a 15 year period, and identify a refined categorization of policy documents used to build the content of a one-stop shop for health policy and systems documents in Uganda to facilitate easy search by users | Document review | 1a |
| Naude, Zani, 2015 [27] | Multi-national | - Policy BUDDIES | NR | NR; study published in 2015 | To perform a situational analysis on the Policy BUDDIES program aimed at enhancing capacity in evidence-informed decision-making (EIDM) of researchers and policymakers; to describe contexts in which health policies are formulated, identify facilitators and barriers to use of research evidence; to determine roles, skills, and resources of regional health policymakers for supporting EIDM; to assess priority areas for research and policymakers in provincial health departments | Interviews  Focus groups | 1a |
| Neves, Lavis, 2014 [28] | Regional | - EVIPNet and   REACH-PI (including ENHRI as a host to EVIPNet Ethiopia and SURE as a funder of EVIPNet Africa) | August 27-31, 2012 | 2012 | To evaluate the International Forum on Evidence Informed Health Policymaking (EIHP) by examining the strengths and weaknesses of the meeting (process measures), potential benefits reported, and intent to utilize the benefits (outcome measures) based on formal feedback from the attendees | Surveys | 2c, 3a, 3b |
| Norton, Howell, 2016 [29] | Global | - USAID’s MCHIP (led by Jhpiego), Save the Children’s Saving Newborn Lives (SNL) Program, programs supported by UNICEF and other major donors | 2012-2013 | 2012 (Bangladesh), 2013 (South Africa) | To assess the effectiveness of two maternal and newborn health technical meetings as knowledge translation interventions, by evaluating whether knowledge gained was used by participants to address global health policy/practice and was shared with other global health practitioners; to identify facilitators and barriers to participant knowledge sharing and use | Surveys  Interviews | 1c, 3b |
| Ongolo-Zogo, Lavis, 2014 [30] | Multi-national | - EVIPNet Cameroon; - REACH-PI Uganda (UNHRO established; collaboration with SURE) | 2001-2012 | 2012 | To describe the evidence-informed health system policymaking activities of two knowledge translation platforms in Uganda and Cameroon through documentary analysis of two historical case studies and an evaluative survey of stakeholders with knowledge of the KTP outputs | Document review  Surveys | 1a, 1b, 1c, 2c, 3a |
| Ongolo-Zogo, Lavis, 2015 [31] | Multi-national | - EVIPNet Cameroon; - REACH-PI Uganda | 2001-2012 | 2001-2006 and 2007-2012 | To assess changes in the climate for evidence-informed health systems policymaking (EIHSP) before and after the implementation of two knowledge translation platforms in Cameroon and Uganda through governmental policy documents | Document review | 1a, 3a, 3b |
| Rispel and Doherty, 2011 [32] | National | - South Africa’s CHP | 2010-2010 | 2010 | To summarize the experience of South Africa’s Centre for Health Policy (CHP) in producing knowledge and supporting health systems development. | Document review  Semi-structured interviews | 1a, 1b, 1c, 2c, 3a, 3b |
| Shroff, Aulakh, 2015 [33] | Multi-national | - SNP programme projects (supported by the AHPSR); - CIPPEC; ICDDR,B; CDBPH; - Innovative Health Research Group; - ZAMFOHR. | 2008-2011 | 2008-2011 | To reflect upon the experience of projects undertaken by the multi-country SNP (sponsoring national processes for evidence-informed policymaking in the health sector of developing countries) program; to understand why projects in certain settings were perceived by key stakeholders to have made progress towards goals, whereas others were perceived not done so well; to illustrate learning points from experiences across 5 countries to inform future evidence-to-policy efforts in LMICs | Document review (reports) | 1b, 1c, 2c, 3a, 3b |
| Uneke, Ezeoha, 2015 [34] | Sub-national | - Ebonyi State Health Policy Advisory Committee (ESHPAC) | 2014-2015 | 2015 | To study the value of policy briefs and dialogues as policymaking mechanisms that allow policymakers to adapt effective evidence-informed policies for infectious diseases of poverty (IDP) control | Questionnaires | 2c |
| Uneke, Ezeoha, 2012 [35] | Sub-national | - ESHPAC | 2009 | 2009 | To study efforts to promote EIHP and improve policymaker and stakeholder capacity to use evidence effectively; to study efforts to encourage linkage and exchange between policymaking process players and bridge the policymaker-research gap, through a one-day workshop to improve capacity for using evidence for policymaking | Questionnaires  Focus Group | 1a, 1b, 1c, 3a |
| Uneke, Ndukwe, 2015 [36] | Sub-national | - ESHPAC | 2011-2013 | 2013 | To study the capacity of the Ebonyi State Health Policy Advisory Committee (ESHPAC) to function as a KTP (following a series of HPAC capacity building initiatives) and equip its members with the competencies required for effective promotion of evidence-informed policymaking | Semi-structured interviews | 1c, 2b, 2c, 3a |
| Yehia and El Jardali, 2015 [37] | National | - Knowledge to Policy Center (K2P) in the American University of Beirut | 2013-2014 | 2014 | To examine the process of influencing the mental health policy agenda in Lebanon through use of KT tools and a KTP (Knowledge to Policy Center [K2P]) as an intermediary between policymakers and researchers | Surveys  Semi-structured interviews | 1c, 2c, 3a, 3b |
| Zida, Lavis, 2017 [38] | National | - SURE health policy rapid response unit, Burkina Faso | March 2011-August 2015 | 2015 | To describe the process of institutionalization of the rapid response service (RRS) in Burkina Faso, and assess the extent of its institutionalization | Interviews  Document review | 1b, 1c, 2b, 2c |

1. Bennett S, Corluka A, Doherty J, Tangcharoensathien V. Approaches to developing the capacity of health policy analysis institutes: a comparative case study. Health Res Policy Syst. 2012;10:7.
2. Bennett S, Corluka A, Doherty J, et al. Influencing policy change: the experience of health think tanks in low- and middle-income countries. Health Policy Plan. 2012;27(3):194-203.
3. Cheung A, Lavis JN, Hamandi A, El-Jardali F, Sachs J, Sewankambo N. Climate for evidence-informed health systems: A print media analysis in 44 low- and middle-income countries that host knowledge-translation platforms. Health Res Policy Syst. 2011;9:7.
4. Cordero C, Delino R, Jeyaseelan L, et al. Funding agencies in low- and middle-income countries: Support for knowledge translation. Bull World Health Organ. 2008;86(7):524-534.
5. Dagenais C, Some TD, Boileau-Falardeau M, McSween-Cadieux E, Ridde V. Collaborative development and implementation of a knowledge brokering program to promote research use in Burkina Faso, West Africa. Global Health Action. 2015;8:1-11.
6. El-Jardali F, Ataya N, Jamal D, Jaafar M. A multi-faceted approach to promote knowledge translation platforms in eastern Mediterranean countries: climate for evidence-informed policy. Health Res Policy Syst. 2012;10:15.
7. El-Jardali F, Jamal D, Ataya N, et al. Health policy and systems research in twelve Eastern Mediterranean countries: a stocktaking of production and gaps (2000-2008). Health Res Policy Syst. 2011;9:39.
8. El-Jardali F, Lavis JN, Moat K, Pantoja T, Ataya N. Capturing lessons learned from evidence-to-policy initiatives through structured reflection. Health Res Policy Syst. 2014;12:2.
9. El-Jardali F, Lavis JN, Ataya N, Jamal D. Use of health systems and policy research evidence in the health policymaking in Eastern Mediterranean countries: views and practices of researchers. Implement Sci. 2012;7:2.
10. El-Jardali F, Lavis JN, Ataya N, Jamal D, Ammar W, Raouf S. Use of health systems evidence by policymakers in eastern Mediterranean countries: views, practices, and contextual influences. BMC Health Serv Res. 2012;12:200.
11. El-Jardali F, Lavis JN, Jamal D, Ataya N, Dimassi H. Evidence-informed health policies in Eastern Mediterranean countries: comparing views of policy makers and researchers. Evid Policy. 2014;10(3):397-420.
12. El-Jardali F, Saleh S, Khodor R, et al. An institutional approach to support the conduct and use of health policy and systems research: The Nodal Institute in the Eastern Mediterranean Region. Health Res Policy Syst. 2015;13:40.
13. Imani-Nasab MH, Seyedin H, Yazdizadeh B, Majdzadeh R. A qualitative assessment of the evidence utilization for health policy-making on the basis of SUPPORT tools in a developing country. Int J Health Policy Manag. 2017;6(8):457-465.
14. Langlois EV, Montekio VB, Young T, Song K, Alcalde-Rabanal J, Tran N. Enhancing evidence informed policymaking in complex health systems: lessons from multi-site collaborative approaches. Health Res Policy Syst. 2016;14:20.
15. Lavis JN, Oxman AD, Moynihan R, Paulsen EJ. Evidence-informed health policy 1 - synthesis of findings from a multi-method study of organizations that support the use of research evidence. Implement Sci. 2008;3:53.
16. Lavis JN, Paulsen EJ, Oxman AD, Moynihan R. Evidence-informed health policy 2 - survey of organizations that support the use of research evidence. Implement Sci. 2008;3:54.
17. Lavis JN, Oxman AD, Moynihan R, Paulsen EJ. Evidence-informed health policy 3 - interviews with the directors of organizations that support the use of research evidence. Implement Sci. 2008;3:55.
18. Lavis JN, Moynihan R, Oxman AD, Paulsen EJ. Evidence-informed health policy 4 - case descriptions of organizations that support the use of research evidence. Implement Sci. 2008;3:56.
19. Law T, Lavis JN, Hamandi A, Cheung A, El-Jardali F. Climate for evidence-informed health systems: a profile of systematic review production in 41 low- and middle-income countries, 1996-2008. J Health Serv Res Policy. 2012;17(1):4-10.
20. Makan A, Fekadu A, Murhar V, et al. Stakeholder analysis of the Programme for Improving Mental health carE (PRIME): Baseline findings. Int J Ment Health Syst. 2015;9(27).
21. Mbonye AK, Magnussen P. Translating health research evidence into policy and practice in Uganda. Malar J. 2013;12:274.
22. Mijumbi-Deve RM, Oxman AD, Panisset U, Sewankambo NK. Feasibility of a rapid response mechanism to meet policymakers' urgent needs for research evidence about health systems in a low income country: a case study. Implement Sci. 2014;9:114.
23. Mijumbi-Deve R, Rosenbaum SE, Oxman AD, Lavis JN, Sewankambo NK. Policymaker experiences with rapid response briefs to address health-system and technology questions in Uganda. Health Res Policy Syst. 2017;15(1):37.
24. Mijumbi-Deve R, Sewankambo NK. A process evaluation to assess contextual factors associated with the uptake of a rapid response service to support health systems’ decision-making in Uganda. Int J Health Policy Manag. 2017;6(10):561-571.
25. Moat KA, Lavis JN, Clancy SJ, El-Jardali F, Pantoja T, Knowledge Translation Platform Evaluation study t. Evidence briefs and deliberative dialogues: perceptions and intentions to act on what was learnt. Bull World Health Organ. 2014;92:20-28.
26. Mutatina B, Basaza R, Obuku E, Lavis JN, Sewankambo N. Identifying and characterising health policy and system-relevant documents in Uganda: a scoping review to develop a framework for the development of a one-stop shop. Health Res Policy Syst. 2017;15:7.
27. Naude CE, Zani B, Ongolo-Zogo P, et al. Research evidence and policy: qualitative study in selected provinces in South Africa and Cameroon. Implement Sci. 2015;10:126.
28. Neves J, Lavis JN, Panisset U, Klint MH. Evaluation of the international forum on evidence informed health policymaking: Addis Ababa, Ethiopia - 27 to 31 August 2012. Health Res Policy Syst. 2014;12:14.
29. Norton TC, Howell C, Reynolds C. Exploratory study of the role of knowledge brokers in translating knowledge to action following global maternal and newborn health technical meetings. Public Health. 2016;140:235-243.
30. Ongolo-Zogo P, Lavis JN, Tomson G, Sewankambo NK. Initiatives supporting evidence informed health system policymaking in Cameroon and Uganda: a comparative historical case study. BMC Health Serv Res. 2014;14:612.
31. Ongolo-Zogo P, Lavis JN, Tomson G, Sewankambo NK. Climate for evidence informed health system policymaking in Cameroon and Uganda before and after the introduction of knowledge translation platforms: a structured review of governmental policy documents. Health Res Policy Syst. 2015;13:2.
32. Rispel LC, Doherty J. Research in support of health systems transformation in South Africa: the experience of the Centre for Health Policy. J Public Health Policy. 2011;32 Suppl 1:S10-29.
33. Shroff Z, Aulakh B, Gilson L, Agyepong IA, El-Jardali F, Ghaffar A. Incorporating research evidence into decision-making processes: researcher and decision-maker perceptions from five low- and middle-income countries. Health Res Policy Syst. 2015;13(70).
34. Uneke CJ, Ezeoha AE, Uro-Chukwu H, et al. Promoting evidence to policy link on the control of infectious diseases of poverty in Nigeria: outcome of a multi-stakeholders policy dialogue. Health Promotion Perspectives. 2015;5(2):104-115.
35. Uneke CJ, Ezeoha AE, Ndukwe CD, Oyibo PG, Onwe F. Promotion of evidence-informed health policymaking in Nigeria: bridging the gap between researchers and policymakers. Global Public Health. 2012;7(7):750-765.
36. Uneke CJ, Ndukwe CD, Ezeoha AA, Uro-Chukwu HC, Ezeonu CT. Implementation of a health policy advisory committee as a knowledge translation platform: the Nigeria experience. Int J Health Policy Manag. 2015;4(3):161-168.
37. Yehia F, El-Jardali F. Applying knowledge translation tools to inform policy: The case of mental health in Lebanon. Health Res Policy Syst. 2015;13:29.
38. Zida A, Lavis JN, Sewankambo NK, Kouyate B, Ouedraogo S. Evaluating the process and extent of institutionalization: a case study of a rapid response unit for health policy in Burkina Faso. Int J Health Policy Manag. 2017;7(1):15-26.

1. NR = not reported; NA = not applicable [↑](#footnote-ref-1)
2. Domains of analytical synthesis framework addressed:

   - 1=descriptive; 1a=context, 1b=infrastructure, 1c=activities/outputs
   - 2=formative; 2a=context, 2b=infrastructure, 2c=activities/outputs
   - 3=summative; 3a=activities/outputs🡪 outcomes, 3b = activities/outputs 🡪 impact
   - 4=linkages; 4a = context🡪infrastructure, 4b = context🡪activities/outputs, 4c= infrastructure🡪activities/outputs; 4d(o/i)=context affects activities/outputs🡪 outcomes/impact, 4e(o/i)=infrastructure affects activities/outputs🡪 outcomes/impact

   [↑](#footnote-ref-2)
