# Supplementary material for: Lessons learned from descriptions and evaluations of knowledge translation platforms supporting evidence-informed policy-making in low- and middle-income countries: a systematic review
Source: Health Res Policy Syst. 2020 Oct 31;18:127. doi: 10.1186/s12961-020-00626-5 (PMC7603785; doi:10.1186/s12961-020-00626-5)
Supplement: Supplementary file 4 — Additional file 4. Quality assessments for included studies. [file 12961_2020_626_MOESM4_ESM.docx]

**Additional file 4:** **Quality assessments for included studies^[[1]](#footnote-1),^^[[2]](#footnote-2),^^[[3]](#footnote-3)^**

| **Lead author, year, citation** | **Two or more data-collection methods employed** | **Random or purposive sampling strategy employed** | **Response rate >60%** | **Two or more types of evidence use examined** | **Two or more types of competing variables examined** | **Score (with scores of formative and/or summative evaluations provided below overall score where applicable)** |
| --- | --- | --- | --- | --- | --- | --- |
| Bennett, Corluka, 2012 [1] | Yes  Interviews  Document review  Formative findings (infrastructure):  No (only interviews) | Partial  Cases: Yes  Interviews: Yes  Documents: NS | No  Interviews: NS  Documents: NA | NA  (linkages findings do not assess impact) | NA  (linkages findings do not assess impact) | 1.5/3  Formative findings: 1/3 |
| Bennett, Corluka, 2012 [2] | Yes  Interviews  Document review  Financial information  Summative findings (activities/outputs🡪 impact):  Point 1: No (only interviews)  Point 2: No (NS) | Partial  Cases: Yes  Interviews: Yes  Documents: Yes  Financial: NS | No  Interviews: NS  Documents: NA  Financial: NA | No  Summative findings (activities/outputs🡪 impact):  Point 1: NS  Point 2: NS | No  Summative findings (activities/outputs🡪 impact):  Point 1: NS  Point 2: NS | 1.5/5  Summative findings (activities/outputs🡪 impact):  Point 1: 1/5  Point 2: 0.5/5 |
| Cheung, Lavis, 2011 [3] | No  Only media analysis | Yes  Cases: Yes  Media analysis: Yes (search algorithms used) | NA | NA  (findings do not assess impact) | NA  (findings do not assess impact) | 1/2 |
| Cordero, Delino, 2008 [4] | Yes  Interviews  Document review | Partial  Cases: Yes  Interviews: Yes  Documents: NS | No  Interviews: NS  Documents: NA | NA  (findings do not assess impact) | NA  (findings do not assess impact) | 1.5/3 |
| Dagenais, Some, 2015 [5] | Yes  Questionnaire  Interviews  Formative findings (activities/outputs):  Point 1: No (only questionnaires)  Point 2: No (only interviews) | Partial  Cases: NS  Questionnaire: Yes  Interviews: Yes | No  Questionnaire: NS  Interviews: NS | NA  (findings do not assess impact) | NA  (findings do not assess impact) | 1.5/3  Formative findings (activities/outputs): 1/3 |
| El-Jardali, Ataya, 2012 [6] | No  Only questionnaires | Cases: Yes  Questionnaires: Yes | Yes | NA  (findings do not assess impact) | NA  (findings do not assess impact) | 2/3 |
| El-Jardali, Jamal, 2011 [7] | No  Documentary analysis | Yes  Cases: Yes  Documents: Yes | NA | NA  (findings do not assess impact) | NA  (findings do not assess impact) | 1/2 |
| El-Jardali, Lavis, 2014 [8] | Yes  Interviews  Document review  Observation of deliberations  Formative findings (activities/outputs):  No (only interviews)  Summative findings:  Points 1-3 (activities/outputs 🡪 outcomes): Yes  Points 4-5 (activities/outputs 🡪impact): No (NS) | Yes  Cases: Yes  Interviews: Yes  Documents: Yes  Deliberations: Yes | No  Interviews: NS  Documents: NA  Deliberations: NA | No | No  NS | 2/5  Formative findings (activities/outputs): 1/3  Summative findings:  Points 1-3: (activities/outputs 🡪 outcomes): 2/5  Points 4-5 (activities/outputs 🡪impact): 1/5 |
| El-Jardali, Lavis, 2012 [9] | No  Only surveys | Yes  Cases: Yes  Respondents: Yes | No  Response rate < 60% | NA  (findings do not assess impact) | NA  (findings do not assess impact) | 1/3 |
| El-Jardali, Lavis, 2012 [10] | No  Only surveys | Yes  Cases: Yes  Respondents: Yes | No  Response rate < 60% | NA  (findings do not assess impact) | NA  (findings do not assess impact) | 1/3 |
| El-Jardali, Lavis, 2014 [11] | No  Only surveys | Yes  Cases: Yes  Respondents: Yes | No  Response rate < 60% in both groups | NA  (findings do not assess impact) | NA  (findings do not assess impact) | 1/3 |
| El-Jardali, Saleh, 2015 [12] | Yes  Questionnaires  Interviews | Yes  Cases: Yes  Respondents: Yes  Interviews: NS | No  Questionnaires: NS  Interviews: NA | NA  (findings do not assess impact) | NA  (findings do not assess impact) | 1/3 |
| Imani-Nasab, Seyedin, 2017 [13] | No  Only interviews | Partial  Case: No  Interviews: Yes | No  NS | NA  (findings do not assess impact) | NA  (findings do not assess impact) | 0.5/3 |
| Langlois, Montekio, 2016 [14] | Yes  Interviews  Document review  Focus group  Formative finding (activities/outputs):  No (NS)  Summative findings:  Point 1 (activities/outputs 🡪 impact): No (NS)  Points 2-3 (activities/outputs 🡪 outcomes): No (only interviews) | Partial  Cases: Yes  Interviews: NS  Document review: NS | No  NS | No  NS | No  NS | 1.5/5  Formative finding (activities/outputs): 0/3  Point 1 (activities/outputs 🡪 impact): 0/5  Points 2-3 (activities/outputs 🡪 outcomes): 0/5 |
| Lavis, Oxman, 2008 [15] | NA  No data-collection method used | NA | NA | NA | NA | NA |
| Lavis, Paulsen, 2008 [16] | No  Only surveys | Yes  Cases: Yes  Participants: Yes | Yes | NA  (findings do not assess impact) | NA  (findings do not assess impact) | 2/3 |
| Lavis, Oxman, 2008 [17] | No  Only interviews | Yes  Cases: Yes  Participants: Yes | Yes | NA  (findings do not assess impact) | NA  (findings do not assess impact) | 2/3 |
| Lavis, Moynihan, 2008 [18] | Yes  Interviews  Document review  Formative findings (infrastructure and activities/outputs): No  (only interviews) | Partial  Cases: Yes  Interviews: Yes  Document review: No | Yes | NA  (findings do not assess impact) | NA  (findings do not assess impact) | 2.5/3  Formative findings (infrastructure and activities/outputs): 2/3 |
| Law, Lavis, 2012 [19] | No  Documentary analysis | Yes  Cases: Yes  Literature review: Yes | NA | NA  (findings do not assess impact) | NA  (findings do not assess impact) | 1/2 |
| Makan, Fekadu, 2015 [20] | Yes  Interviews  Focus group discussion assessments  Survey | Partial  Cases: Yes  Interviews: NS  Survey: Yes | NS | NA  (findings do not assess impact) | NA  (findings do not assess impact) | 1.5/3 |
| Mbonye and Magnussen, 2013 [21] | No  Only semi-structured questionnaires | Yes  Case: Yes  Questionnaire: Yes | No  NS | NA  (findings do not assess impact) | NA  (findings do not assess impact) | 1/3 |
| Mijumbi, Oxman, 2014 [22] | No  Only questionnaires  Interviews not reported in the results | Yes  Case: Yes  Questionnaire: Yes | No  NS (only reported for the pilot phase) | No  NS | No  NS | 1/5 |
| Mijumbi-Deve, Rosenbaum, 2017 [23] | No  Only interviews (for user testing) | Partial  Case: NS  Interviewees: Yes | Yes | NA  (findings do not assess impact) | NA  (findings do not assess impact) | 1.5/3 |
| Mijumbi-Deve and Sewankambo, 2017 [24] | No  Only in-depth interviews | Yes  Case: Yes  Interviews: Yes | No  NS | NA  (findings do not assess impact) | NA  (findings do not assess impact) | 1/3 |
| Moat, Lavis, 2014 [25] | Yes  Independent questionnaires | Yes  Cases: Yes  Respondents: Yes | No  Response rate < 60% | Yes  Conceptual  Symbolic | No  Only individuals | 3/5 |
| Mutatina, Basaza, 2017 [26] | No  Only document review | Partial  Case: No  Review: Yes | NA | NA  (findings do not assess impact) | NA  (findings do not assess impact) | 0.5/2 |
| Naude, Zani, 2015 [27] | Yes  Interviews  Focus groups | Partial  Cases: No  Interviews: Yes  Focus group: NS | No  NS | NA  (findings do not assess impact) | NA  (findings do not assess impact) | 1.5/3 |
| Neves, Lavis, 2014 [28] | No  Only surveys | Yes  Cases: Yes  Survey: Yes | No  Response rate < 60% | No  NS | No  NS | 1/5 |
| Norton, Howell, 2016 [29] | Yes  Surveys  Interviews  Summative findings (activities/outputs 🡪 impact):  No  Only surveys | Yes  Cases: Yes  Survey: Yes  Interviews: Yes | No  Surveys: No  Interviews: No | No  NS | No | 2/5  Summative findings (activities/outputs 🡪 impact): 1/5 |
| Ongolo-Zogo, Lavis, 2014 [30] | Yes  Document review  Surveys | Partial  Cases: No  Documents: Yes  Survey: Yes | No  Documents: NA  Survey: NS | NA  (findings do not assess impact) | NA  (findings do not assess impact) | 1.5/3 |
| Ongolo-Zogo, Lavis, 2015 [31] | No  Only document review | Yes  Cases: Yes  Documents: Yes | NA | No  Only instrumental use | No | 1/4 |
| Rispel and Doherty, 2011 [32] | Yes  Document reviews  Semi-structured interviews  Formative finding (activities/outputs):  No  Only document review  Summative findings (activities/outputs 🡪 outcomes and activities/outputs 🡪 impact):  Yes | Partial  Case: NS  Documents: NS  Interviews: Yes | No  Documents: NA  Interviews: NS | Yes  Instrumental  Conceptual | No | 2.5/5  Formative finding (activities/outputs): 0/3  Summative findings (activities/outputs 🡪 outcomes and activities/outputs 🡪 impact):  2.5/5 |
| Shroff, Aulakh, 2015 [33] | No  Document review (reports) | Yes  Cases: Yes  Reports: Yes | NA | No  NS | No | 1/5 |
| Uneke, Ezeoha, 2015 [34] | No  Only questionnaires | Yes  Case: Yes  Questionnaire: Yes | Yes | NA  (findings do not assess impact) | NA  (findings do not assess impact) | 2/3 |
| Uneke, Ezeoha, 2012 [35] | Yes  Questionnaires  Focus Group | Partial  Case: NS  Questionnaires: Yes  Focus group: NS | Yes | NA  (findings do not assess impact) | NA  (findings do not assess impact) | 2.5/3 |
| Uneke, Ndukwe, 2015 [36] | No  Only semi-structured interviews | No  Case: No  Interviews: NS | No  NS | NA  (findings do not assess impact) | NA  (findings do not assess impact) | 0/3 |
| Yehia and El Jardali, 2015 [37] | Yes  Surveys  Semi-structured interviews | Yes  Case: Yes  Surveys: Yes  Interviews: Yes | Partial  Policy brief and policy dialogue survey: No (response rate < 60%)  Post-dialogue survey: Yes  Interviews: Yes | No  NS | No  NS | 2.5/5 |
| Zida, Lavis, 2017 [38] | Yes  Interviews  Document review  Formative findings  (activities/outputs and infrastructure):  No  Only interviews | No  Case: No  Interviews: NS  Documents: NS | No  NS | NA  (findings do not assess impact) | NA  (findings do not assess impact) | 1/3  Formative findings  (activities/outputs and infrastructure):  0/3 |

1. Citations for references presented in column 1 correspond to numbered reference list presented in Additional file 3 [↑](#footnote-ref-1)
2. NS = not stated; NA = not applicable [↑](#footnote-ref-2)
3. Articles were scored out of 5 points if they present at least one summative or linkages findings assessing impact (not outcomes); articles were scored out of 3 points if they present formative findings, or summative or linkages finding not assessing impact (e.g., context🡪activities/outputs, context affect activities/outputs🡪outcomes) [↑](#footnote-ref-3)
