# Supplementary material for: Lessons learned from descriptions and evaluations of knowledge translation platforms supporting evidence-informed policy-making in low- and middle-income countries: a systematic review
Source: Health Res Policy Syst. 2020 Oct 31;18:127. doi: 10.1186/s12961-020-00626-5 (PMC7603785; doi:10.1186/s12961-020-00626-5)
Supplement: Supplementary file 5 — Additional file 5. Summary of findings from included empirical studies. [file 12961_2020_626_MOESM5_ESM.docx]

**Additional file 5: Summary of findings from included empirical studies^[[1]](#footnote-1),^^[[2]](#footnote-2)^**

| **Lead authors, year, citation** | **Focus of article** | **Key findings** | **Time period studied** | **Last year data were collected** | **Jurisdictional focus of the studied organization** | **Types of evaluations included in study** | **KT platform addressed in study** |
| --- | --- | --- | --- | --- | --- | --- | --- |
| Bennett, Corluka, 2012 [1] | To assess the factors that facilitate health policy analysis institutes (HPAIs) in LMICs, and understand the nature of support for capacity development for these institutes through analysis of comparative case studies of six HPAIs (via document review, semi-structured interviews with stakeholders, and discussion with institute staff regarding preliminary findings) | - **Study overview**   - Six case study institutes were identified (two NGOs, two university-owned institutes, and two government-owned institutes in both Asia and Africa) through purposively sampling from a complete list of HPAIs in LMICs - **Descriptive (infrastructure)**   - **Financial resources:** three institutes received external grants for start-up, and two of these three institutes soon collapsed; five of the six institutes experienced high administrative costs and unpredictability due to their reliance on short-term donor funding, and could not sustain itself from other funding; none of the institutions had a clear financial/fundraising strategy; the approximate annual budget for the KTPs were from US$26.000 (Uganda) to US$1.300.000 (Vietnam)   - **Human resources:** all organizations except CHeSS relied on in-house research staff primarily; retention of skilled human resources was perceived by HPAIs to be a central facilitator of institute success, but was in fact problematic at all of institutes but one; once having acquired skills and experiences, staff moved to better paid positions elsewhere, leaving heavy workloads to remaining senior staff; the total number of staff for KTPs were from 1 (Uganda) to 42 (Vietnam)   - **Governance and management:** the structure and roles of boards varied based on institute ownership; boards promoted continuity, independence, and raised funds, ultimately contributing significantly to organizational capacity   - **Governance and management:** HPAIs based in Ministries of Health faced critical governance and management issues due to lack of separate governing bodies outside of ministry of health channels - **Formative (infrastructure)**   - “In contexts where strong boards were in place they were perceived to play a very positive role”   - Governance and management: HPAIs perceived routine management systems as not problematic - **Linkages (context 🡪 infrastructure)**   - Political transitions and institutional rivalry emerged as barriers to financial sustainability - **Linkages (infrastructure 🡪 activities/outputs)**   - **Networks**: linking institutes to policymakers allowed for facilitation of policy influences and support for research capacity**;** external networks with research organizations helped promote capacity, especially in situations where long term institutional collaborations existed; three institutes benefitted from longer term collaborations with organizations outside of their country | 2009 | 2009 | **Focus:** multi-national  Vietnam, India, Bangladesh;  South Africa, Uganda, Ghana | 1b, 2b, 4a, 4c | - Health Strategy and Policy Institute (HSPI), Vietnam; - Health Economics Unit (HEU), South Africa; - Institute for Health Systems (IHS), India; - Health Economics Institute (HEI), Bangladesh; - Health Policy Analysis Unit (HPAU), Uganda; - Centre for Health and Social Services (CHeSS), Ghana |
| Bennett, Corluka, 2012 [2] | To study the contributions of HPAIs to health policy agenda setting, formulation, implementation, and monitoring and evaluation processes in LMICs, and to assess facilitating factors (organizational form/structure) for HPAIs contributing positively to health policy in LMICs; study conducted via case study approach, through document review, financial information, semi-structured interviews of key informants, and discussion of report with HPAI staff members | - **Study Overview**   - Six case study institutes were identified (one NGO, one university-owned, and one government-owned institute in both Asia and Africa) through purposively sampling from a complete list of HPAIs in LMICs - **Descriptive (context)**   - Facilitators identified as critical in supporting effective policy engagement emerged from the analysis, including supportive policy environment - **Descriptive (infrastructure)**   - 3 institutes were established by respected individuals in the field, and 3 were established through organizational agreements   - Facilitators identified as critical in supporting effective policy engagement emerged from the analysis, including independence in governance and financing,   - Facilitators identified as critical in supporting effective policy engagement emerged from the analysis, including strong linkages to policymakers which foster trust and influence   - HPAIs located within government faced substantial difficulties and barriers to supporting effective policy engagement, although the formal relationship of institute to government was not found to be critical - **Descriptive (activities/outputs)**   - All 6 HPAIs demonstrated active engagement in providing policy advice   - 6 organizations (although HPAU did these only occasionally) conducted policy-relevant research, responded to ad hoc requests from government for analyses or policy briefs, produced research publications, participated in government meetings, and established informal contacts with policymakers/stakeholders   - Activities/outputs by HPAIs were undertaken in response to donor and government requests   - **Activities:** few organizations were involved in conducting policy dialogues at national levels actively (CHeSS was the only one, although HEU and HEI did occasionally); HIS and HEU conducted policy dialogues at international levels but only did this occasionally   - **Outputs:** few organizations were involved in conducting systematic reviews (HEU was the only one, albeit infrequently); none of the organizations were involved with commissioning research or reviews   - **Outputs:** primary outputs from most HPAIs were research reports, often in conjunction with verbal briefings (all organizations did this frequently, except HPAU which did this occasionally); only HEU and IHS publish books/chapters and articles in peer-reviewed journals   - **Activities:** HEI and HEU conducted training and capacity development for policy makers in an actively engaged manner; CHeSS and IHS intend to do this but do not currently   - **Activities:** all organizations actively and frequently provided policy advice and technical assistance in policy formulation and evaluation - **Summative (activities/outputs 🡪 impact)**   - 3 organizations (HSPI, HEU, IHS) have had a major impact via contributions to specific policy processes (excerpt: all 6 HPAIs demonstrated active engagement in policy advice)   - 6 organizations (all but HPAU) were involved in influencing policy indirectly through research publications and influencing policy formally through participation in government meetings | 2009-2010 | 2010 | **Focus:** multi-national  Vietnam, India, Bangladesh;  South Africa, Uganda, Ghana | 1a, 1b, 1c, 3b | - Health Strategy and Policy Institute (HSPI), Vietnam; - Health Economics Unit (HEU), South Africa; - Institute for Health Systems (IHS), India; - Health Economics Institute (HEI), Bangladesh; - Health Policy Analysis Unit (HPAU), Uganda; - Centre for Health and Social Services (CHeSS), Ghana |
| Cheung, Lavis, 2011 [3] | To examine whether and how policymakers, stakeholders, and researchers discuss health policy priorities, research evidence, and health policy dialogues in the print media in order to assess climate for evidence-informed health systems and provide a baseline for KT platform evaluation. | - **Descriptive (context)**   - There were 5.5 and 5 more articles identifying health research evidence in comparison to policy priorities and dialogues, respectively   - Only 31 (5%) articles on health research evidence discussed systematic reviews (logical unit to be the focus of supporting research use) and only 27(2%) discussed research on health systems   - The majority of the 264 articles mentioning policy priorities attributed to government officials as opposed to stakeholders and researchers   - Only 27 (2%) of the 1468 articles on health research evidence mentioned health systems research   - 940 (76%) of the 1241 articles mentioning location of researchers indicated that local researchers were involved; 380 (31%) mentioned participation of outside researchers   - 287 (99%) and 283 (98%) of the 290 articles describing policy dialogues addressing health issues involved government officials and stakeholders, respectively; only 27 (9%) involved researcher involvement   - 30 of the 32 jurisdictions had little print media coverage on the three topics (with 3 or fewer articles on policy priorities and dialogues) in comparison to articles identified in China and Uganda | 2007 | NR; search conducted for the year 2007 | **Focus:** global  44 countries (47 jurisdictions) in Africa, the Americas, Asia, and the eastern Mediterranean | 1a | - EVIPNet and other KT platforms |
| Cordero, Delino, 2008 [4] | To describe how health research funding agencies support knowledge translation through their funded research into policy in LMICs through analysis of semi-structured interviews with key informants of 23 national and international funding agencies, and with document review from funding agencies web sites. | - **Descriptive (context)**   - 13 of 23 agencies mentioned that there is a favorable political climate to knowledge translation, due to realization that research needs to infiltrate policy and actions to benefit health.   - Some barriers identified by respondents were the lack of tools and funding for knowledge translation, and the little involvement of key stakeholders in the research process. - **Descriptive (infrastructure):**   - 18 of 23 agencies present a commitment to knowledge translation in their mandates, but with a lack of common terminology   - 9 of 23 agencies presented a mandate of enhancing health equity or poverty reduction through KT   - 8 of 23 agencies put knowledge translation as a high priority issue, and they have less than 20% of their budget allocated to knowledge translation.   - National funding agencies put forward greater efforts in KT in comparison with international agencies - **Descriptive (activities/outputs):**   - Most activities included traditional efforts to disseminate information to a broad audience, including web sites and publications   - 13 of 23 agencies encouraged linkage/exchange activities (e.g., stakeholder setting research agenda, decision-maker networks/programs, etc.) between potential users and researchers   - 6 of 23 agencies described “facilitating pull” initiatives (e.g., decision maker workshops, tools development, etc.) to stimulate interest in research from decision-makers   - 13 of 23 agencies described evaluation of KT activities | 2003-2004 | 2004 | **Focus:** global  Brazil, Colombia, India, Philippines, South Africa, Thailand | 1a, 1b, 1c | - 23 health research funding agencies operating in part as KT platforms |
| Dagenais, Some, 2015 [5] | To present the collaborative development of a knowledge brokering (KB) strategy implemented in Burkina Faso, and evaluate its implementation at year 1 | - **Study Overview**   - The KB strategy synthesis began with a scoping study was initially undertaken to ensure most recent studies were considered   - Two one-day workshops were conducted to understand the central issue of low research use in health policymaking, and to adapt the KB strategy to the local context - **Descriptive (infrastructure):**   - The KB program was developed based on the scoping studies and workshop, brokers were recruited and trained, and the program was evaluated after one year of implementation - **Descriptive (activities/outputs)**   - **Activities:** program implementation and outputs **–** the knowledge broker carried out his role, following the logic model outlined at the onset of the project   - **Outputs:** 11 reports were produced (2 literature reviews and reports on activities such as deliberative workshops and action plans), 30 documents were prepared to disseminate   - **Activities:** two deliberative workshops (based on literature synthesis and policy briefs) were organized to answer high-priority stakeholder questions; 50 individuals participated in at least one KB activity - **Formative (activities/outputs)**   - **Reactions to training activities:** training and preparatory activities were highly regarded by all participants (range 3.6-4 on a 4-point scale), and content was considered useful to their work   - **Analysis of implementation processes:** the KB role, activities, and knowledge transferred were viewed as important and useful by all users/participants that were targeted by the activities/outputs; strengths included broker/speaker competencies, activities/outputs meeting stakeholder needs, and high/sustained participation level; these users/participants made suggestions for program improvements, highlighting in particular the need to involve decision-makers at the central level and more clearly defining the broker’s role/tasks | 2011-2013 | 2013 | **Focus:** national  Burkina Faso | 1b, 1c, 2c | - KB program in district of Kaya, Burkina Faso |
| El-Jardali, Ataya, 2012 [6] | To assess the climate for evidence-informed policy in the Eastern Mediterranean Region (EMR), explore current processes and weaknesses of health policymaking, identify priorities (e.g., policy brief short-term requirements) and country-specific requirements for establishing KTPs, through questionnaires delivered to several policymakers and senior health systems researchers in an international meeting. | - **Study overview**   - 65 participants were invited to participate (based on interest and participation in EVIPNet EMR), of which 42 participated in the study (response rate = 64.6%); 25 were policymakers and stakeholders, and 17 were researchers - **Descriptive (context)**   - In EMR countries, policymakers and researchers rarely engaged in KT activities and interaction between the two was limited (“half of respondents indicated that policymakers very rarely/rarely interact with researchers as part of priority-setting processes or for conducting primary research or systematic reviews”)   - Baseline level of activities/outputs was low; research evidence on high priority policy issues was not often made available – “more than half of respondents indicated that evidence on high priority policy issues was very rarely/rarely disseminated to policymakers”   - Policymakers very rarely/rarely identified areas for utilizing evidence on high priority policy issues, or participated in training to find and use research evidence and assess quality and local applicability   - The complexity of policymaking was highlighted due to donors, political regimes, economic goals, and outdated laws   - Primary weaknesses were identified as policymaker strategic thinking, need to make quick decisions, scarce financial resources, and lack of competent and trained human resources   - When asked about windows of opportunity for action over the next 6-12 months, development of new national strategic plans, changes in government, and new WHO EMRO strategic directions for scaling up health research were recurring themes regarding promoting KT and use of evidence in health policies   - Priority topics frequently mentioned to be developed in short-term policy briefs and evidence needs were national health insurances and universal health coverage.   - Networking/partnerships for the platform, assembling a core team, and policymaker support advocacy were identified as key country-specific requirements for KT platforms; key barriers to KT activities included lack of communication, lack of financial resources, policy-relevant research (particularly systematic reviews), lack of experience documentation, lack of mechanisms to utilize tacit knowledge, and the subjective nature of decision-making processes; also, weak MOH infrastructure, lack of governance, political sensitivity of the findings, low motivation for KT and utilizing evidence, lack of capacity for research use, and lack of alignment between policy and research priorities   - The most commonly planned KT activities were developing policy briefs and policy dialogues | 2010 | 2010 | **Focus:** regional  *Eastern Mediterranean Region (EMR):*  Algeria, Bahrain, Egypt, Iran, Jordan, Lebanon, Oman, Sudan, Syria, Tunisia, and Yemen | 1a | - EVIPNet EMR |
| El-Jardali, Jamal, 2011 [7] | To profile research production and output on health policy and systems research (HPSR), by identifying publications between 2000 and 2008 in 12 countries in the Eastern Mediterranean Region, identify gaps in production, and assess whether existing HPSR addresses regional priorities around financing, human resources, and the non-state sector in health | - **Descriptive (context)**   - 1,487 (11.94%) articles met the criteria outlined on the coding sheet; Syria (36.94%), Bahrain (33.04%), and Palestine (24.32%) had the largest number of articles fitting coding criteria; Egypt (7.02%), Tunisia (7.40%), and Libya (9.94%) had the fewest number of articles fitting criteria - An increase in HPSR production from 2000 to 2008 occurred, peaking after 2005; steady increase particularly in Jordan, Egypt, Lebanon, Sudan, and Tunisia - 68.1% of articles focused on delivery arrangements, and 24.4% addressed implementation strategies; 10% of articles discussed governance arrangements, 2.1% discussed financial arrangements - Among articles that addressed delivery arrangements, 36.5% addressed the category “to whom care is provided and with what efforts to reach them” - In analyzing which of the previously identified priorities are already addressed by existing research, 39% of HPSR addressed human resources for health priorities (with most articles providing information on gaps in education/training programs), 12% addressed health financing priorities, 6.1% of the role of the non-state sector | 2000-2008 | 2008 | **Focus:** regional  *Eastern Mediterranean Region (EMR):*  12 countries – Bahrain, Egypt, Jordan, Lebanon, Libya, Morocco, Oman, Palestine, Sudan, Syria, Tunisia, Yemen | 1a | - EVIPNet EMR |
| El-Jardali, Lavis, 2014 [8] | To gain a better understanding of knowledge translation platforms (KTPs) in low and middle-income countries (LMICs) by examining i) activities conducted by KTPs, ii) the way in which these activities and their outputs are perceived by KTP leaders, policymakers, and stakeholders, iii) factors supporting and challenges impeding KTP work (and lessons learned), and, iv) factors ensuring KTP sustainability | - **Study overview**   - 23 KTP leaders and 17 policymakers and stakeholders from 10 KTPs were part of the interviews, along with 7 KTPE members; KTPs were 2 or 3 years old in each country. - **Descriptive (context)**   - Accessing and finding local evidence is difficult, as is the unwillingness of policymakers and other research institutions to share the data they possess   - Top level policymakers experience high turnover in government, a resistance to change, strong political influences, and difficulty bringing policymakers, researchers, and stakeholders together exist as challenges to enhancing EIHP - **Descriptive (infrastructure)**   - A lack of skilled and dedicated human resources to pursue push efforts and poor quality of local information aligning with high priority policy issues exist   - Ensuring sustainability of EIHP initiatives after removal of funding was a major challenge for KTPs   - KTP institutionalization within government has helped retain human resources and secure funding; university hosting of KTPs provides the advantage of autonomy from political interests   - A key need identified (to facilitate sustainability of KTPs) was around building and retaining capacity - **Descriptive (activities/outputs)**   - **Activities***:* Limited efforts by KTPs under research production; 3 KTPs built capacity for conducting systematic reviews and undertaking priority-setting exercise, 6 KTPs conducted priority-setting exercises with policymakers prior to EIHP activities   - **Outputs:** 2 KTPs produced systematic reviews   - Identified a lack of local research production and urgent need to build production of high quality, policy-relevant systematic reviews and local single studies   - All KTPs built their capacity to develop evidence briefs tailored to policy; all KTPs developed evidence briefs   - **Activities:** 4 KTPs are in the process of implementing rapid response services (RRS), 5 in the process of creating online clearinghouses   - **Activities:** 1 KTP began to assess and enhance capacity of research users to acquire, assess, adapt, and apply research   - Identified need for improving monitoring and evaluation efforts, as they were seldom undertaken by KTPs in LMICs due to lack of capacity (e.g., approaches, tools, data analysis)   - **Activities:** 4 KTPs evaluated evidence briefs, deliberative dialogues, and capacity building sessions through pre/post intervention questionnaires - **Formative (activities/outputs)**   - Evidence briefs were commonly perceived to be very effective tools for influencing policymaking processes   - Deliberative dialogues informed by evidence briefs were identified by interviewees as the most commendable tools for enhancing EIHP   - Interviewees mentioned as a key outcome of EIHP activities an increased awareness on the importance of EIHP initiatives   - There is a prevailing perception among KTP leaders that M&E activities are challenging initiatives - **Summative (activities/outputs 🡪 outcomes)**   - 7 KTPs reported they have increased awareness of importance of initiatives supporting EIHP and   - 8 KTPs reported strengthened relationships between policymakers, stakeholders, researchers   - 6 KTPs reported higher policymaker demand for KT products - **Summative (activities/outputs 🡪 impact)**   - All KTPs organized deliberative dialogues informed by evidence briefs, some of which had influenced policymaking processes   - 6 KTPs reported evidence briefs produced having a direct impact in informing policymaking at government level - **Linkages (context 🡪 activities/outputs)**   - …political will can facilitate these activities/outputs - **Linkages (infrastructure 🡪 activities/outputs)**   - Strong leadership…can facilitate these activities/outputs   - Facilitators to supporting and enhancing EIHP activities include skilled human resources to moderate the deliberative processes, as well as policymaker/stakeholder support   - KTP activities were facilitated by support from policymakers, stakeholders and international funders; KTPs were impeded by lack of skilled human resources to conduct EIHP activities and gaps in infrastructure (e.g., internet connection) | 2012 | 2012 | **Focus:** global  Argentina, Bangladesh, Nigeria, Burkina Faso, Cameroon, Central African Republic, Ethiopia, Uganda, Sudan, Zambia | 1a, 1b, 1c, 2c, 3a, 3b, 4b, 4c | - Evidence to Policy (E2P) Argentina; - E2P Bangladesh; - E2P Nigeria; - EVIPNet Burkina Faso; - EVIPNet Cameroon; - EVIPNet Central African Republic; - EVIPNet Ethiopia; - REACH-PI, Uganda; - KTP Sudan; - ZAMFOHR |
| El-Jardali, Lavis, 2012 [9] | To explore the views and experiences of researchers regarding the role of health policy and systems evidence in policymaking in 12 Eastern Mediterranean countries (i.e., facilitators, barriers, influencing factors of evidence in policymaking) | - **Study Overview:**   - 56% response rate (133 of 238 researchers asked to complete survey) - **Descriptive (context)**   - 67.2% of researchers indicated transferring results to other researchers, 40.5% to other policymakers in government   - 14.5% indicated that they produced policy briefs, 24.4% indicated that they disseminated messages specifying possible actions, 20% indicated that they provided research results through the web, 23% indicated that they had long-term collaborations with policymakers/stakeholders providing technical assistance, 16% indicated they interacted with policymakers and stakeholders in priority setting, 19.8% indicated that policymakers and stakeholders were involved in their research   - 66% of researchers believed they have enough training to produce systematic reviews, but only 48% have ever conducted/produced a systematic review; 43% indicated that policymakers and stakeholders show little regard for value of evidence   - Many items were indicated as hindering the use of evidence in policymaking – 67.9% indicated insufficient policy dialogue and researcher/policymaker/stakeholder collaboration opportunities, 66% indicated practical constraints to implementation, 61.3% indicated non-receptive policy environments, 57.7% indicated politically sensitive findings   - Involving policymakers and stakeholders at various stages of the policymaking process (i.e., through priority setting exercises and technical assistance provision) were factors that increase researchers’ engagement in KTE activities   - 58% indicated they have undertaken KTE activities, and that the best strategy for KT is through face-to-face contacts   - Researchers increase the transfer of evidence to policymakers when they develop relationships with media journalist to promote use of evidence.   - Transfer of research to provider organization is decreased when long-term formal collaborations with policymakers and stakeholders for capacity development is increased.   - Researchers increase the transfer of evidence to policymakers by each unit increase in the availability of international funds for HPSR. | 2000-2008 | NR; study published in 2012 | **Focus:** regional  *Eastern Mediterranean Region (EMR):*  12 countries selected based on interest and participation in EVIPNet EMR launch meeting in January 2009 – Bahrain, Egypt, Jordan, Lebanon, Libya, Morocco, Oman, Palestine, Sudan, Syria, Tunisia, and Yemen | 1a | - EVIPNet EMR |
| El-Jardali, Lavis, 2012 [10] | To explore policymakers views and experiences regarding the use of health systems evidence in policymaking in 10 Eastern Mediterranean countries (i.e., facilitators, barriers, influencing factors of evidence in policymaking) | - **Study Overview:**   - 56.3% response rate (237 policymakers completed the survey) of the 421 policymakers requested - **Descriptive (context)**   - Strong influences on the policymaking process were indicated to be lack of coordination across ministries and between government and health providers (74.9%), governing parties (53.9%), limited funding in the health sectors, and donor organizations (79.2%).   - 88.5% of policymakers reported requesting evidence from researchers, and 43.1% reported collaboration with researchers; 69.7% indicated participated in priority-setting processes with researchers   - Policymakers have no negative attitudes towards the use of research evidence, and its benefits for the policy-making process.   - Jordan and Yemen reported facing challenges in the health policymaking process (Yemen also with using evidence in the process) due to government relations across ministries and between health providers   - 40.1% of policymakers reported the lack of timeliness of research evidence delivery (i.e., not delivered at the right time)   - Policymakers believed that various factors limited the use of research evidence including 55.3% reporting a lack of a budget for evidence-informed health policymaking (EIHP), 52.6% reporting a lack of an administrative structure supporting EIHP, and 35.9% reporting limited value given to research   - Barriers to evidence use – lack of research targeting health policy, lack of funding/investments, and forces of a political nature   - Facilitators of evidence use – policy relevant health research availability and research institutions, qualified researchers, funding, easy information access   - 52.1% of respondents reported to use evidence whenever it is available, whereas 48.5% request evidence for the policy-making process. | 2010 | 2010 | **Focus:** regional  *Eastern Mediterranean Region (EMR):*  10 countries selected based on interest and participation in EVIPNet EMR launch meeting in January 2009 – Algeria, Bahrain, Jordan, Lebanon, Oman, Pakistan, Palestine, Sudan, Tunisia, and Yemen | 1a | - EVIPNet EMR; - Middle East and North Africa (MENA) Health Policy Forum |
| El-Jardali, Lavis, 2014 [11] | To conduct comparative analysis (via data from two separate surveys) about policymakers and researchers views and practices, and the use of health systems evidence in policymaking within EMR countries | - **Descriptive (context)**   - There is a wide gap between policymakers and researchers in comparing perceptions on factors influencing policymaking processes and use of evidence   - Factors that had the strongest influence on policymaking as reported by policymakers and researchers include: limited health funding and donor organizations; the strongest factors hindering health policymaking were lack of coordination across ministries and in government/provider relations   - For researchers, values of political parties, health funding/resources, and donor organizations, as well as influence of private insurers were perceived to strongly influence policymaking   - For policymakers, strong influencing factors on policymaking process included: research about health problems, limited health funding, and donor organizations   - 43% of researchers indicated policymakers show little regard to the value of evidence, but 89% of policymakers indicated they look/ask for scientific evidence to support policymaking activities; however, 36% policymakers indicated political actors do not value evidence in policymaking   - Policymakers and researchers confirmed that policymakers have access to health research through internet, and 53% of policymakers reported receiving training for use in policymaking   - 43% of policy makers mentioned to have contact and collaborate with researchers, whereas only 29% of researchers reported to frequently/always try to involve policymakers in their work.   - Researchers and policymakers identified hindering factors for evidence use in policymaking: lack of funding for health research, overriding political forces, lack of political will and corruption, and lack of trained policymakers for finding and using evidence; lack of policy-relevant research was also cited by policymakers, and lack of communication/dialogue was cited by researchers   - Facilitators to evidence use in policymaking identified by researchers and policymakers included: availability of policy-relevant research, availability for funding for health research, policymaker belief in importance of evidence-informed policymaking, and wide dissemination of research   - Specific areas were identified for undertaking KT activities and implementing interventions to bridge the gap between policymakers and stakeholders; these included: increasing health research funding, improving dissemination, conducting sensitization/awareness workshops, improving contact/exchange, conducting policy relevant research, and improving incentives/legislations for policymakers to use evidence   - Establishing evidence-to-policy support units was mentioned by many policymakers (7% of responses) as improving EIHP   - 25% of policymakers mentioned to have an explicit budget allocated to research and evidence-informed policy making; researchers indicated that there is national (34%), regional (38%) and international (50%) funding available to conduct HPSR. | NR | NR; study published in 2014 | **Focus:** regional  *Eastern Mediterranean Region (EMR):*  Policymakers from 10 countries surveyed – Algeria, Bahrain, Jordan, Lebanon, Oman, Pakistan, Palestine, Sudan, Tunisia, Yemen  Researchers from 12 countries surveyed – Bahrain, Egypt, Iran, Jordan, Lebanon, Libya, Morocco, Oman, Palestine, Sudan, Syria, Yemen  Countries were selected based on interest and participation in EVIPNet EMR launch meeting in January 2009 | 1a | - EVIPNet EMR; - Middle East and North Africa (MENA) Health Policy Forum |
| El-Jardali, Saleh, 2015 [12] | To offer a structured reflection on establishment and functioning of the EMR HPSR Nodal Institute and three sub-nodes, focusing on approaches used to support HPSR, activities conducted, methods used to apply these activities, and outcomes of these activities | - **Descriptive (context)**   - Mapping of EMR academic/research institutions identified 50 institutions, 32 of which were engaged in HPSR; 84% of the 32 institutions were involved in information/evidence, 78% in service delivery, 53% involved in leadership and governance, 34% concerned with human resources for health, and 41% in health systems financing   - Commonly identified regional areas of HPSR focus for these institutions included quality of healthcare services, patient safety, human resources for health, and management of non-communicable disease   - Commonly identified regional HPSR challenges amongst these institutions included: lack of incentives for partnerships between stakeholders, presence of poor research environments and knowledge translation/dissemination, weak data systems for health information, low interest in evidence-informed policy, lack of priority setting, lack of funding, lack of research workforce, and lack of collaborations   - Country-specific HPSR priorities were identified (via validation/ranking questionnaires in stakeholders in three countries); cross-cutting HPSR priorities between countries included issues related to primary healthcare, non-communicable diseases, and human resources for health; priorities varied depending on type of stakeholder | 2013-2014 | 2014 | **Focus:** regional  *Eastern Mediterranean Region (EMR):*  Lebanon;  Bahrain, Jordan, Tunisia | 1a | - Nodal Institute (Lebanon); - 3 Sub-Nodes (Bahrain, Jordan, Tunisia) |
| Imani-Nasab, Seyedin, 2017 [13] – *N.B. based on authors’ recommendation, this article was substituted for another similar article with a full-text that could be not be accessed.* | To qualitatively describe the process of evidence utilization for developing policy documents in the Iranian Ministry of Health and Medical Education (MoHME) and compare these findings with the process recommended by SUPPORT tools, in order to understand evidence-informed policymaking and the feasibility of these tools in a developing country | - **Descriptive (context)**   - *Acquisition of evidence:* Interviewees acquired research evidence for effectiveness of policy options (using scientific databases on the internet) and feasibility of policy options (using routine data and ideas/experiences of stakeholders); participants searched for evidence in thematic (topic-based) form, were more likely to assess economic and technical feasibility rather than social/political feasibility, and were less likely to search based on the evidence pyramid. A small number of interviewees actually searched for qualitative studies to assess policy options.   - *Assessment of evidence:* none of the participants used standard critical appraisal tools such as CASP and GRADE to assess study methodological quality; participants preferred to commission research and directly control the production of studies; most participants doubted the quality of routine data, and used simple methods of evidence assessment   - *Synthesizing evidence and adapting policy options to local conditions:* participants did not use ideas and experiences of all stakeholders (but did use some); participants did use published domestic evidence and results of pilot implementations to adapt policy options   - *Application of evidence:* participants did prepare evidence-based policy documents, but in these documents did not take into consideration the window of opportunity, used highly technical terms, did not incorporate user-friendly summaries, and did not present alternative policy options   - Participants did pay attention to financial burden of policy issues, performed sensitivity analysis of the preferred policy option on feasibility, advocacy from scholars; participants also used multi-criteria decision-making models to prioritize policy options, implemented policy based on readiness of policy-implementing units, and classified policy documents based on the urgency of the policy issues | NR | NR; study published in 2017 | **Focus:** national  Iran | 1a | - National Parliament research center; establishment of the Council of Health Policy in the Iranian MoHME; - National Institute for Health Research and Health Technology Assessment Bureau in MOHME |
| Langlois, Montekio, 2016 [14] | To reflect upon the implementation and impact of two multi-site evidence-to-policy interventions (based on iterative exchanges between researchers and policymakers) in LMICs using a mixed-method approach (document review, interviews, focus group discussions); these interventions were implemented by the Alliance for Health Policy & System Research programme of work entitled “Leadership Development for Enhanced Decision Making” | - **Descriptive (context)**   - Policy BUDDIES (Policy BUilding Demand for evidence in Decision-making through Interaction and Enhancing Skills) – there is a need to strengthen/support capacities of policymakers in using evidence syntheses; institutional support and incentives for using evidence are important barriers/facilitators to knowledge generation and use - **Descriptive (activities/outputs)**   - *Cameroon:* implementation of policies was the focus (whereas in South Africa development and implementation were both focuses) - **Formative (activities/outputs)**   - Policy BUDDIES – it was perceived that collaborations between researchers and policymakers need to be meaningful, equal, trusting - **Summative (activities/outputs 🡪 outcomes)**   - Policy BUDDIES enhanced the capacity of subnational policymakers to ask for, demand, and use systematic review evidence (and other products of evidence syntheses) to inform policymaking   - *South Africa:* Policymakers reported that Policy BUDDIES helped them understand the value of research evidence to daily work, fostering greater demand for policy-relevant knowledge; policymakers expressed “new appetite for science” and a need for more evidence on implementation and operational strategies - **Summative (activities/outputs 🡪 impact)**   - *South Africa:* Policy BUDDIES engagements contributed to specific policy debate on decentralization of antiretroviral initiation/maintenance (where researcher buddies translated systematic review findings to action by policymakers) and contributed to mother-to-child HIV transmission prevention (where buddies appraised guidelines for policymakers) - **Linkages (context 🡪activities/outputs)**   - *Cameroon:* uptake of the “buddying” aspect of the approach was implemented much more slowly; at the time of the study, Cameroon was responding to a poliomyelitis outbreak which limited time and resources available to policymakers to engage in buddying process - **DATA EXCLUDED FROM SYNTHESIS – NOT A KT PLATFORM:**   - **‘INSP approach’** supported implementation research using communities of practice in Mexico and Nicaragua; targeted frontline policy/programme managers and implementers; systematized tacit knowledge and implementation research findings through workshops and online communities of peers; engagement of researchers was critical; peer learning facilitated problem solving   - *Mexico:* periodic health organizations utilized evaluation forms to periodically monitor the quality of maternal health program care following the strong ownership of implementation research findings by CoPs; CoPs also addressed problems with rotation of personnel at state and local levels; INSP improved capacity to identify and use evidence to solve problems with implementation   - *Nicaragua:* although two sanitary emergencies were an obstacle for timely achievement of goals, CoP structure assisted in outbreak and management control of chikungunya and dengue, demonstrating responsiveness of the health system | 2013-2015 | 2015 | **Focus:** multi-national  South Africa, Cameroon | 1a, 1c, 2c, 3a, 3b, 4b | - *Intervention:*   Policy BUDDIES program operating in two countries, embedded within a broader initiative sponsored by the Alliance for Health Policy & Systems Research programme of work entitled “Leadership Development for Enhanced Decision Making” |
| Lavis, Oxman, 2008 [15] | To identify organizations around the world (specifically in LMICs) that successfully or innovatively support the use of research evidence in developing clinical practice guidelines (CPGs), health technology assessments (HTAs), and health policies; to describe the experiences of these organizations (**via synthesis of findings from 3 phases: a survey, interviews, case descriptions from site visits; article 1**) | *N.B. Four articles from same overarching study were included, but only three had extractable empirical data. The first in the four-part series was a synthesis and overview of the subsequent three articles [16-18].* | NR | NR; study published in 2008 | **Focus:** global  *Countries addressed are discussed in the subsequent three articles.* | *See [16-18] for details about the three sub-studies.* | *See below for details about the three sub-studies.* |
| Lavis, Paulsen, 2008 [16] | To identify organizations around the world (specifically in LMICs) that successfully or innovatively support the use of research evidence in developing clinical practice guidelines (CPGs), health technology assessments (HTAs), and health policies; to describe the experiences of these organizations (**via an e-mail survey of organizations’ senior staff members; article 2**) | - **Study overview:**   - [Of 176 organizations who were sent questionnaires, 152 (86%) completed questionnaires; 95 organizations produce CPGs, HTAs, or both]   - 57 units support government policymaking (government support units; GSUs), of a total of 152 organizations that responded   - [85 organizations (56%) were from high-income countries, 19 (13%) from upper middle-income countries] - **Descriptive (context)**   - 36 organizations (24%) were from lower-middle income countries; 8 produced CPGs alone, 1 produced HTAs alone, 9 produced both CPGs and HTAs, and 18 were GSUs   - 8 organizations (5%) were from low income countries; 0 produced CPGs alone, 0 produced HTAs alone, 2 produced CPGs and HTAs, and 6 were GSUs   - 19 organizations (12.5%) were from upper-middle income countries; 11 of these were GSUs   - Few organizations identified in Eastern Europe (n=1), India (n=2), the Middle East (n=3) or China (n=4) - **Descriptive (infrastructure)**   - A high proportion of organizations producing CPGs, HTAs, or both also support policymaking (17 CPG organizations, 16 HTA organizations, and 45 CPG+HTA organizations), whereas GSUs were much less likely to produce CPGs or HTAs (17 produced CPGs, 11 produced HTAs)   - Most organizations (producing CPGs, HTAs or both) had five or fewer full-time equivalent (FTE) staff   - GSUs were often based in academic institutions (37%) and government agencies (39%); GSUs were most focused on public health (88%), primary healthcare (72%), and healthy public policy (67%), providing services on many facets of policy issues - **Descriptive (activities/outputs)**   - A higher percentage of GSUs involved target users (public policymakers in health departments, those in central agencies, stakeholders, and other public policymakers) in guiding the selection of their topics/services, in comparison to CPG- or HTA-producing organizations   - “GSUs are most likely to convene expert meetings to discuss available research (82%), undertake short-term research projects (79%)”   - More than 80% of organizations (CPG-, HTA-producing organizations, and GSUs) provide panels with or using systematic reviews   - GSUs use various explicit evidence-valuation processes, but none as frequently as CPG-, HTA-, or CPG- and HTA-producing organizations, which prioritize evidence by its quality   - **Activities:** 32(56%) GSUs do not collect data about uptake systematically, and 29(51%) GSUs do not systematically evaluate their impact in other ways   - More than half of organizations develop strategies to increase the capacity of target users to acquire, assess, and use their product or services.   - 49 (86%) of 57 GSU provided or employed systematic reviews   - 14% of GSUs have products submitted to any form of clearinghouse | NR | NR; study published in 2008 | **Focus:** global  *35 GSUs from low-, lower-middle income, and upper-middle income countries (from a total of 57 GSUs)*  *N.B. full list of organizations not provided in original source* | 1a, 1b, 1c | - 24 GSUs   (from a total of 44 total organizations in LMICs studied) |
| Lavis, Oxman, 2008 [17] | To identify organizations around the world (specifically in LMICs) that successfully or innovatively support the use of research evidence in developing clinical practice guidelines (CPGs), health technology assessments (HTAs), and health policies; to describe the experiences of these organizations (**via interviews with directors of organizations; article 3**) | - **Study Overview:**   - [The director (or his/her nominee) was interviewed in 25 organizations] - **Descriptive (context)**   - 12 of these 25 organizations were GSUs (while 5 produce CPGs, 3 produce HTAs, and 5 produce both CPGs and HTAs)   - Close links with policymakers was consistently viewed as a strength by GSUs, whereas organizations producing CPGs, HTAs, or both had conflicting viewpoints about this   - Implementation weaknesses were focused on rather than strengths by all organizations, with few exceptions; GSUs reported examples of success which were more complex (given the complexity of the research-to-policy pathway), such as policymakers that based their decisions on work of the organization; GSU failures typically involved perceptions that policymakers were not basing their decisions in part on the organization’s work - **Descriptive (infrastructure)**   - “Organizations varied in size from a few people to 50 people”   - 6 GSUs commissioned little to no work (instead producing it internally), 4 commissioned some, and 2 commissioned half of their work - **Descriptive (activities/outputs)**   - GSUs were involved with a range of activities including producing systematic reviews (n=3), conducting policy analyses (n=3), training and capacity building (n=3), producing CPGs (n=2), conducting evaluations (n-2), conducting economic analyses (n=2), conducting health systems research (n=2), and taken on consultations and communication activities (n=2)   - [HTA- and CPG+HTA-producing organizations also conducted horizon scanning, preparing policy papers, and conducting evaluations]   - [The most commonly cited strength among all organizations was “being evidence-based”, or using rigorous, systematic, and transparent methods]   - 7 of 12 GSUs respond to requests for applications, 2 respond to perceived policy needs, and 1 make decisions by asking involving staff and the Minister of Health.   - **Outputs:** GSUs were less likely to conduct or use systematic reviews (3 of 12), to have a manual that described their methods (4 of 12), and more likely to report using non-systematic reviews of the literature (3 of 12) - **NOT INCLUDED IN SYNTHESIS AS NOT EMPIRICAL DATA:**   - [Seven pieces of advice were offered to those attempting to establish similar organizations: 1) collaborate with other organizations; 2) create strong links with policymakers and other stakeholders; 3) work independently and address conflicts of interest for those involved; 4) build capacity with those in the organization; 5) apply good methodologies and adhere to transparency; 6) start small and address meaningful questions; 7) be attentive to considerations around implementation]   - [One common piece of advice was offered to the World Health Organization and other international organizations/networks: to foster collaborations across organizations] | NR | NR; study published in 2008 | **Focus:** global  *25 organizations:*  6 in Western Europe, 5 in North America, 4 in Asia, 3 in Latin America, 2 in Africa, 2 in Eastern Europe, 2 in the Middle East, and 1 in Australia | 1a, 1b, 1c | - 12 GSUs (from a total of 25 organizations included) |
| Lavis, Moynihan, 2008 [18] | To identify organizations around the world (specifically in LMICs) that successfully or innovatively support the use of research evidence in developing clinical practice guidelines (CPGs), health technology assessments (HTAs), and health policies; to describe the experiences of these organizations (**via case descriptions of organizations that support the use of research evidence; article 4**) | - **Study Overview:**   - 8 site visits (cases) were conducted, during which 51 interviews took place with the director, 1-2 staff members, an advocate, and at least one critic at each organization   - Four of the eight organizations studied acted at least in part as knowledge translation platforms, including: Regional East African Community Health Policy Initiative (REACH-PI), East Africa; Thailand (research units that informed the implementation and evaluation of the universal health program); Free State, South Africa (long term relationships between provincial policymakers and researchers); and Mexico Seguro Popular initiative (an effort to draw on evidence to inform health insurance scheme policy) - **NOT INCLUDED IN SYNTHESIS AS NOT KT PLATFORMS:**   - [The other four organizations studied (not acting as KT platforms) were: Pharmaceutical Benefits Scheme, Australia and South Africa (evidence-based drug assessment and pricing scheme); Philippines (initiative to address conflicts of interest and inequity of clinical practice guidelines); Chile (initiative to use CPGs to make best use of scarce resources); National Institute for Health and Clinical Excellence, UK (unit producing CPGs and HTAs in an evidence-based manner)] - **Descriptive (context):**    - Two key suggestions for the World Health Organization and other organizations/networks were offered by participants: mobilize government support, financial resources, and/or both policymaker and researcher participation; and, create knowledge-related global public goods - **Descriptive (infrastructure)**   - A lack of financial and human resources (particularly in East Africa, South Africa, and Thailand) and presence of conflicts of interest (as in Thailand [where researchers have close relationships with policymakers, distorting research and raising concerns if research contracts policymakers] and South Africa [where tension arose between researchers and policymakers]) were two organizational weaknesses cited repeatedly by individuals participating in the site visits   - [Use of an evidence-based approach (although time consuming) and existence of a strong relationship between policymakers and researchers (often challenged by conflicts of interest) were two organizational strengths cited repeatedly by individuals participating in the site visits]   - [Two types of advice for those establishing or working in similar organizations was suggested by site visit participants: learn from other organizations, develop capacity among skilled staff and collaborators, retain these individuals, involve all possible stakeholders in discussions around establishing a new similar organization, find success in process/methods from the beginning, obtain strong political commitment, and consider equity] - **Formative (infrastructure)**    - REACH-PI (East Africa) offered unique views and perspectives on researcher-policymaker relationships: 1) a model developed in the local context is more likely to succeed, 2) high-level political support is necessary for any initiative that supports decision-makers become make evidence-informed health systems decisions; and 3) any initiative involving an intermediary that would broker researcher-policymaker relationships would be promising   - Site visit participants from only three organizations identified capacity, specifically long-term investments in human and/or institutional resources as a strength   - Participants from two organizations focused on North-South partnerships as a strength (as exists in Australia, and will soon exist in the United Kingdom although North-North partnerships exist there) - **Formative (activities/outputs)**   - Participants from site visits at only three organizations highlighted as a strength their organizations’ effort to produce highly relevant products (operational research, systematic reviews, CPGs, or HTAs), disseminate these products or facilitate access to them   - In South Africa, focus on operational research to guide program development was cited as a strength   - In Thailand, focus on operational research and disseminating this research was cited as a strength   - For REACH, focus on operational research and systematic reviews, proactively disseminating this evidence, and facilitating access were cited as strengths | NR | NR; study published in 2008 | **Focus:** global  *Africa (3)*:  East Africa (1; Kenya, Tanzania, Uganda), South Africa (1 directly, 1 in partnership with Australia)  *Asia (2)*:  Thailand (1), Philippines (1)  *Latin America (2):*  Chile (1), Mexico (1)  *United Kingdom (1)*  *N.B. 6/8 countries are LMICs* | 1a, 1b, 2b, 2c | - REACH-PI;   Thailand research units;   - Free State, South Africa researcher-policymaker partnership; - Mexico Seguro Popular initiative |
| Law, Lavis, 2012 [19] | To profile published systematic review production between 1996 and 2008 that had authors based in 41 countries (or targeted these countries) that host KT facilities in order to assess climate for evidence-informed health systems | - **Descriptive (context)**   - Three-fold (for Africa in Medline) and 110-fold (for Asia in Embase) increase in systematic review (SR) production between period 1 (1996-2002) and period 2 (2003-2008)   - China accounted for more SRs than other countries in Asia (n=837); Brazil in the Americas (n=628); Iran in the Mediterranean (n=99); Nigeria in Africa (n=98)   - China was more often the home of corresponding authors than any other country in the second period; Brazil produced most systematic reviews (n=395) in Americas; Nigeria produced the most (n=53) in Africa; Iran produced the most (n=67) in the Eastern Mediterranean   - China was more often the target of reviews than other countries (n=609) in the second period; Brazil in the Americas (n=207); Nigeria in Africa (n=65); Pakistan in the eastern Mediterranean (n=23)   - No systematic reviews were produced by a corresponding author in nine countries or targeted five countries   - 48 of the 2,063 reviews identified through Medline and Embase addressed health system topics; delivery arrangements (n=43), governance (n=6), and financial arrangements (n=4) were addressed by most   - 35 of the 830 health systems reviews identified through Health Systems Evidence addressed these countries | 1996-2008 | Search conducted for the period 1996-2008 | **Focus:** global  *41 countries (44 jurisdictions)* in Africa, the Americas, Asia, and the Eastern Mediterranean Region | 1a | - EVIPNet and other KT platforms |
| Makan, Fekadu, 2015 [20] | To conduct stakeholder analyses in 5 countries participating in the PRogramme for Improving Mental health carE (PRIME), evaluate a template for cross-country comparison of the analyses, and assess the utility of stakeholder analysis in identifying and characterizing support for various actions in mental health policy and systems research | - **Descriptive (context)**   - Using qualitative stakeholder analysis, the most powerful/supportive actors to target evidence uptake towards and for scaling up mental health care in PRIME countries include: policymakers (WHO, ministries of health, non-health sector ministries/parliament), donors (department for international development [DFID] UK, DFID country offices, other donor agencies), the media (national, district), and universities   - To evaluate cross-country stakeholder power and positions (particularly for prioritizing potential stakeholder engagement) for scaling up mental health services, force field analysis was conducted to classify stakeholders based on influence and supportiveness, ultimately identifying the most highly influential and highly supportive stakeholders; medium-highly influential stakeholders that are supportive can be targeted next; specific stakeholders varied in each of the 5 PRIME countries | NR | NR; study published in 2015 | **Focus:** multi-national  Ethiopia, India, Nepal, South Africa, Uganda | 1a | - PRIME |
| Mbonye and Magnussen, 2013 [21] | To present the results and lessons learned of four annual research-to-policy workshops involving researchers, policymakers, civil society, and media. Workshop reports were reviewed, and semi-structured interviews were conducted to participants. | - **Descriptive (context)**   - Media in an advocacy role and mid-level policymakers (soon moving to senior level) are key to facilitating evidence-to-policy processes - **Descriptive (activities/outputs)**   - Between 2006 and 2009, 4 workshops were held   - 322 participants attended in total over the four years, with consistently high turnout from mid-level policymakers, researchers, and media   - 83.4% of participants attended all 4 workshops; 46% increase in number of participants since original workshop   - Workshops generated interest leading to presentation and discussions of research results that were relevant in the national health context   - Several policies were reviewed and some were revised to incorporate evidence from and outside of the workshops including: Reproductive Health Policy Guidelines (revised to allow community based provision of Injectable Depo-provera), Integrated Community Case management of childhood illnesses, Village Health team strategy, Malaria Control Policy, Medical male circumcision, Infant and Young child feeding, Prevention of Mother to Child Transmission, and the Road Map for improving maternal and newborn care - **Formative (activities/outputs)**   - Policy brief writing skills were honed through the workshops, as participants participated in the policy review process and engaged in dialogue with policymakers - **Summative (activities/outputs 🡪 outcomes)**   - Reported benefits from 98 participants at the last workshop included increased scientific knowledge (20.4%), had improved their networks with researchers (8.2%), had written policy briefs (6.2%), appreciated the importance of research and evidence-based decision-making (12.2%), and got to know new areas for research and methods (11.2%)   - Other benefits included learning about research areas and methods, and networking with researchers | 2006-2009 | 2009 | **Focus:** national  Uganda | 1a, 1c, 2c, 3a | - Uganda National Health Research Organization (UNHRO) |
| Mijumbi, Oxman, 2014 [22] | To assess the feasibility of a rapid response mechanism in Uganda, with the goal of elucidating its performance in meeting policymakers’ needs for health systems research evidence in a timely and relevant manner | - **Descriptive (infrastructure)**   - Staff included researchers with a background in medical or social/population studies, coupled with research method skills; also needed writing and policy analysis skills and an understanding of the health system and policy formulation process - **Descriptive (activities/outputs)**   - During the pilot phase, 12 policymakers were purposively offered the service, 10 responded with intentions to use it, and 9 of the 10 ended up using it   - Service received 65 evidence requests from 30 stakeholders and policymakers in the first 28 months; the majority of these (23 of the 30) individuals were from the Ministry of Health   - Governance (17 of the 65) and organization of health systems (14 of the 65) were the most common evidence requests   - Average duration in which answers were needed was 13 (12.8) days; modal time for response was 21 days, two questions required responses in 24 and 48 hours each, and another six questions required response in 5 days; maximum time given for a request was 28 days   - 81.5% (53 of 65) responses were returned on time, 10.8% (7 out of 65) were returned late), and 7.7% (5 of 65) were not followed through to the end   - The commonest need for a question to be answered was to contribute to meetings and debates with peers/superiors/development partners; others included preparations for press conferences, input into strategic planning documents, advice to subordinates, proposals and concepts to management or development partners   - Active sensitization of policymakers about the rapid response service produced an increase in the number of questions, in particular at four time-points - **Formative (activities/outputs)**   - 42 of 65 individuals were satisfied with the evidence they had received, and 16 were very satisfied; findings indicate that this is a promising KT strategy   - An important factor in response to and uptake of the service was regular contact between researchers and policymakers at the response service - **Summative (activities/outputs 🡪 impact)**    - 30 of the 65 questions led to policymakers changing their course of action, with 15 feeling confident and 14 feeling very confident about their course of action – overall, policymakers involved in policymaking processes had increased confidence as a result of the rapid response briefs | March 2010-July 2012 | 2012 | **Focus:** national  Uganda | 1b, 1c, 2c, 3b | - REACH-PI (rapid response service) |
| Mijumbi-Deve, Rosenbaum, 2017 [23] | To explore the experiences of Ugandan policymakers with a rapid response service brief template, use this feedback to improve the template format, and evaluate how well revised brief templates met policymaker needs (via user testing and interviews) | - **Study overview**   - Twelve user tests were carried out with 8 purposively sampled participants, four (of seven invited) using the original template in 2010 and four (of eight invited) using the second iteration in 2012; four participants of these original eight were also invited in 2013 to evaluate the third template   - Of the eight participants, five of which were from the ministry of health, two from civil society, and one development partner - **Formative (activities/outputs)**   - All participants indicated positive experiences with the briefs and their formats, specifically finding the format “usable, credible, desirable, and of value”; participants did not identify any major problems with the brief   - Participants viewed certain brief format features favourably, including: clarity of presentation, use of tables to summarize findings, balance between detail and precision of presented information, clear background section establishing brief context   - Participants did, however, identify big problems/frustrations with various aspects of the brief, such as the absence of recommendations, lack of clarity regarding the type of brief and its potential uses (especially first time users), and a crowded front page”   - Feedback for brief improvement included: using a reference style that showed in-text citations, providing recommendations from authors, and increasing visibility of briefs amongst target groups   - Conflicting feedback was offered by participants on preferred length of the briefs (e.g., two respondent felt that seven pages was too long, while all others reported preferring this length, some indicating that more information could be added to the briefs), where partner logos were placed (some viewing these should be moved to the last page rather than the first, other feeling they improved credibility), and other minor distracting features (e.g., five of eight felt that the information boxes on first page were distracting/irrelevant/misplaced but three found these useful particularly for those who had not used the service before, and two felt there were too many brief provider information boxes although others found this improved credibility)   - Participants indicated divided preferences for the older vs. newer formats of the brief; two participants preferred the revised template (version 3, with a five page maximum, additional information provided separately, condensed information boxes, reducing logos, among other changes), while two preferred the older one (version 2); version 3 was preferred because the face page was less crowded, and no reasons were given for why version 2 was preferred (only that the new version looked deficient)   - Although preferences for version 2 vs. version 3 were divided, “none of the participants felt there were still any big problems with the final version (version 3)” | 2010-2013 | 2013 | **Focus:** national  Uganda | 2c | - REACH-PI (partnered with WHO’s EVIPNet and SURE project) |
| Mijumbi-Deve and Sewankambo, 2017 [24] | To explore contextual factors associated with how and why a rapid response service (RRS) may be taken up by users in Uganda and understand how these factors impact implementation and scale-up of RRSs in similar settings (following piloting an RRS beginning in 2010), through a case study using process evaluation methods (i.e., interviews with researchers, knowledge translation specialists, and policymakers in Uganda’s health sector) | - **Study overview:**   - 21 respondents were interviewed (11 [52%] health systems and KT researchers, and 10 [48%] policymakers)   - 7/21 respondents were university-affiliated, and 6/21 were affiliated with the Ugandan government ministry of health   - Three contextual thematic categories emerged as influencing the utilization of the RRS: internal factors (those over which the RRS and management had full or almost full control), external factors (those over which the RRS management had only partial influence), and environmental factors (those over which the RRS had no control or remote control); simple (i.e., an overlapping concentric model) and complex (i.e., indirect interactive relationships between descriptive categories) relationships emerged between the factors, which were inter-related and not independent of each other - **Formative (activities/outputs)**   - Design (internal factor #1) – to increase chances of uptake, respondents indicated that the design of the service must take into consideration the needs of its users, fill these needs, remain aware of the needs of users throughout its operation (i.e., via consultation with policymakers), must work within current norms and behavior of users, must be simple and user-friendly; respondents indicated increased uptake if there is a balance between demand and supply sides (moderating user expectations as the operation is scaled up and meeting them appropriately), as well as providing opportunities for feedback from users (i.e., being a ‘personal’ service that meets user needs rather than a one-size-fits-all service), and ensuring the monitoring and evaluation component is designed around user satisfaction rather than simply use - **Linkages (context 🡪 activities/outputs)**   - Political will (environmental factor #1) – respondents indicated that RRS work could be taken up by users more easily with explicit support from high level policymakers by increasing awareness and legitimacy   - Health system policy and decision-making infrastructure (environmental factor #2) – one researcher and two policymakers noted setup of systems/bodies for policy and decision-making was important for uptake, improving the culture of evidence-informed policymaking amongst policymakers and in turn use of these mechanisms; they noted that it would be useful to have clear steps in decision-making process where evidence must be considered, and open recognition of RRSs and other similar services - **Linkages (infrastructure 🡪 activities/outputs)**   - Resources (internal factor #2) – to increase chances of uptake, respondents agreed that there must be adequate: human resources (skilled/qualified on the supply side with current knowledge of research and policymaking, social skills [as was present in the Ugandan RRS], and communication skills, and training for new human resources), time (allowing for maturity of the service and for it to become known, and for researchers to have protected time to conduct activities required), and finances (to ensure all other inputs are strong and the service is efficient)   - Visibility (external factor #1) – to increase chances of uptake, respondents agreed that there must be adequate visibility with respect to: knowledge of its existence (as only some policymakers knew about the RRS), branding (e.g., of documents), consistent name (e.g., the RRS in Uganda had been called REACH-PI service, SURE project service, Makerere University’s service, and just RRS, which all create confusion), clarity of goals (i.e., whether it is “a service to policymakers or a research project”, which was unclear in this case), physical location (many felt that being at a prominent university may give automatic visibility, but one policymaker felt this might cause lack of visibility and that the ministry of health might be better as target users are there), and continuity of activities   - Integrity (external factor #2) – all respondents indicated integrity (i.e., who is running the service and their partners), credibility (i.e., location of service, as being at a university gave a sign of neutrality), and trustworthiness (with some feeling that the location at a university inspires trust and others feeling that it inspires mistrust) as being important for RRS uptake   - Networks and relationships (external factor #3) – respondents indicated that networks and relationships can improve uptake through continued interaction, collaborations on the supply side (carefully balancing institutionalized systems and personal relationships, with both policymakers and stakeholders, and supporting researchers), use of ‘champions’ (i.e., policymakers who can advocate and sensitize their peers to the RRS), and advocacy (e.g., in the form of repeated reminders to users) | March 2010-May 2014 | 2014 | **Focus:** national  Uganda | 2c, 4b, 4c | - REACH-PI (partnered with WHO’s EVIPNet and SURE project) - RRS at the College of Health Sciences in Makerere University (formerly called REACH-PI service, SURE project service, Makerere University’s service, and the RRS) |
| Moat, Lavis, 2014 [25] | To evaluate the usefulness of and assess views on evidence briefs and deliberative dialogues through implementation of questionnaire-based surveys in support of evidence-informed policymaking in six African nations | - **Descriptive (activities/outputs)**   - 304 (57%) and 303 (57%) of 530 individuals who read evidence briefs (on 17 priority issues) and attended dialogues completed questionnaires on the briefs and dialogues, respectively   - Cameroon had the largest number of respondents for evidence brief surveys (n=99) and dialogue surveys (n=77)   - The most frequently reported category of professional role on the evidence brief survey was policymaker (49%) followed by stakeholder (24%), researcher (49%), and other (5%); for the dialogue survey, policymaker (49%), stakeholder (23%), researcher (10%) and other (4%)   - Only 52% of evidence briefs took quality considerations into account when discussing evidence or underwent a merit review; only 62% took local applicability of research evidence into account - **Formative (activities/outputs)**   - Respondents viewed evidence briefs and deliberative dialogues (and key features) very favourably, regardless of country, issue, or group involved   - “Not concluding with recommendations” and “not aiming for consensus” were viewed as the least favourable features of the briefs and dialogues, respectively   - In the regression models for evidence briefs, self-reported professional role in the “other” category was a significant predictor of giving “not concluding with recommendations” a lower helpfulness score (P=0.028)   - In t-tests for deliberative dialogues, respondents without past research experience gave “not aiming for consensus” significantly lower scores for helpfulness than those with experience (P=0.015) - **Summative (activities/outputs 🡪 impact)**   - Respondents generally reported strong intentions to act on what they had learnt through evidence briefs and deliberative dialogues; those who did not provide a role category considered themselves to have less behavioural control and less likely to act on what they had learnt | 2009 | 2009 | **Focus:** regional  *6 sub-Saharan African countries:* Burkina Faso, Cameroon, Ethiopia, Nigeria, Uganda, Zambia | 1c, 2c, 3b | - EVIPNet and other KT platforms |
| Mutatina, Basaza, 2017 [26] | To conduct a scoping review of relevant Uganda-specific health policy and systems documents produced over a 15 year period, and identify a refined categorization of policy documents used to build the content of a one-stop shop for health policy and systems documents in Uganda to facilitate easy search by users (via document review) | - **Descriptive (context)**   - 265 documents were identified as health policy and systems-relevant documents, including rapid response summaries (18%), guidelines (13%), policies, plans, policy evidence briefs, strategies (13%), and policy dialogue reports (3%)   - The most frequently addressed clusters of national priority areas in the documents included: governance, coordination, monitoring and evaluation (74, 28%); disease prevention, mitigation, and control (63, 24%); and, health education, promotion, and environmental health and nutrition (41, 16%)   - The least frequently addressed clusters of national priority areas in the documents included: curative, palliative, rehabilitative services and health infrastructure, each addressed in three documents (1%); and early childhood development in one document.   - Regarding national health priority areas, policies (46%), guidelines (31%), and policy evidence briefs (18%) most frequently addressed the cluster of disease prevention, mitigation, and control; strategies (90%) and rapid response summaries (17%) most frequently covered the cluster on health education, promotion, environmental health and nutrition; plans (36%) most frequently addressed the cluster on governance, coordination, monitoring and evaluation   - Regarding health systems topics, the greatest number of documents (n=101, 38%) addressed delivery arrangements, followed by implementation strategies (56, 21%), and finally financial arrangements (16, 6%)   - Delivery arrangements domain was addressed by most “other reports” (93%), policy evidence briefs (33%), and rapid response summaries (25%); governance arrangements domain was addressed by 63% of guidelines, 49% of policies, and 30% of rapid response summaries; financial arrangements domain was addressed by policy evidence briefs (12%), rapid response summaries (17%), and other reports (5%); most plans (96%) covered implementation strategy, and no other policy or other report covered this domain   - Between 2000 to 2011, there was a consistent increase in the volume of documents followed by a decline; the 6 documents per year in 2000 increased to 49 per year in 2011, and then 27 per year in 2014; the distribution of the various document types over the 15 years was not uniform | January 2000-December 2014 | 2014 | **Focus:** national  Uganda | 1a | - REACH-PI (specifically it’s clearinghouse service entitled the Uganda Clearinghouse for Health Policy and Systems) |
| Naude, Zani, 2015 [27] | To perform a situational analysis (via in-depth interviews and focus groups) on the Policy BUDDIES program aimed at enhancing capacity in evidence-informed decision-making (EIDM) of researchers and policymakers; to describe contexts in which health policies are formulated, identify facilitators and barriers to use of research evidence; to determine roles, skills, and resources of regional health policymakers for supporting EIDM; to assess priority areas for research and policymakers in provincial health departments | - **Study Overview:**   - 9 interviews with 10 participants (managers of clusters of services/units) were conducted in provincial DoH in South Africa; 4 focus group discussions (1 per region) were conducted in Cameroon with a range of policymakers - **Descriptive (context)**   - Policymaking processes were described as lengthy, complex, and iterative, involving back-and-forth consultations with diverse stakeholder groups   - Policymaking processes are influenced by political structures, national and subnational level relationships, funding, and agendas of international stakeholders. They also mention that research is not necessarily the main driver used for policymaking   - Participants from South Africa were more aware of systematic reviews (as sources of synthesized best evidence), while participants from Cameroon highlighted main sources of evidence to be best practices in high performing districts and learning from failing districts (based on surveys and public health data)   - Contextual realities, costs, logistics, and human resources (clinicians, NGOs, funders) influence policy; while research plays a role, it is not the main driver of policy   - Research evidence is perceived by many as unavailable, inaccessible, poorly timed, or not applicable to local contexts; research evidence on the internet was viewed as possibly unreliable   - EIDM is not as well understood in Cameroon as in South Africa, where it is viewed as necessary   - Barriers to EIDM in both countries included insufficient time, capacity, lack of incentives for operational research/EIDM at the regional level, and bureaucratic processes   - Facilitators of EIDM include good relationships/interactions between policymakers and researchers, willingness of policymakers to enhance EIDM skills (particularly finding relevant/reliable online resources, and critical appraisal), capacity building regarding health information analysis   - Positive policymaker-researcher interactions are perceived as beneficial in research/policy formulation processes, as they allow for raising researcher awareness of implementation challenges and allows policymaker needs to be shared for the creation of tailored and focused strategies; this was more evident in South Africa than in Cameroon, where there are limited interactions between researchers and policymakers   - Researchers must have a good understanding of policymaking environment to influence policymaking processes   - Participants expressed desire for opportunities for links between researchers and policymakers, and for research-related capacity development activities | NR | NR; study published in 2015 | **Focus**: multi-national  South Africa, Cameroon | 1a | - Policy BUDDIES |
| Neves, Lavis, 2014 [28] | To evaluate the International Forum on Evidence Informed Health Policymaking (EIHP) by examining the strengths and weaknesses of the meeting (process measures), potential benefits reported, and intent to utilize the benefits (outcome measures) based on formal feedback from the attendees collected via a survey answered during the forum. | - **Study overview:**   - 67 of approximately 121 attendees filled out a questionnaire (55% response rate), of which 40/75 were researchers (53% response rate), 11/23 policymakers (48% response rate), 4/8 journalists (50% response rate), and 12/15 attendees (80% response rate) who did not report a role - **Formative (activities/outputs)**   - 100% of participants reported that they would attend the International Forum again in the future; on a scale of 1-5, participants rated the overall programme and components (4.4 [0.7]) very highly   - Participants rated plenary sessions above 4.0 on a scale of 1-5; low- or middle-income countries (LMIC) participants rated the panel discussion a 3.2 [1.0] average, the lowest rating by LMIC participants for all ratings, corresponding to certain written comments regarding the lack of LMIC speakers   - The top three benefits reported by all demographics of participants were i) sharing experiences and lessons learned (75%); ii) new opportunities for future collaboration (69%); and iii) new knowledge (67%)   - 12 participants noted that pre-meeting organization logistics could have been strengthened; many participants reported that there was too much in the programme; 7 participants highlighted country experience presentations and 6 highlighted the impact evaluation/analysis sessions as their favourites; 4 participants highlighted a need to explore EIHP sustainability and 4 suggested need for capacity building sessions - **Summative (activities/outputs 🡪 outcomes)**   - Number of participants reporting a certain benefit did not directly correspond to participants’ intentions to utilize the benefit in their work in a meaningful way; for example, few participants reported new skills as a benefit overall (31 (46%)) but 13 (19%) reported an intent to utilize new skills - S**ummative (activities/outputs** 🡪 **impact)**   - 25% or less of meeting participants reported an intent to utilize any potential benefits highlighted in the questionnaire, except for the benefit of pursuing new opportunities for future collaboration (26 (39%)) | August 27-31, 2012 | 2012 | **Focus**: regional  Participants from LMICs were present; International Forum held at Addis Ababa, Ethiopia | 2c, 3a, 3b | - EVIPNet; - REACH-PI (including ENHRI as a host to EVIPNet Ethiopia and SURE as a funder of EVIPNet Africa) |
| Norton, Howell, 2016 [29] | To assess the effectiveness of two maternal and newborn health technical meetings as knowledge translation interventions, by evaluating whether knowledge gained was used by participants to address global health policy/practice and was shared with other global health practitioners; to identify facilitators and barriers to participant knowledge sharing and use. Participants were surveyed and interviewed in 2012. | - **Study overview:**   - Of 411 participants in the 2012 Bangladesh meeting, 148 participants from 22 countries completed the survey. Eleven of these respondents (from eight countries) were interviewed.   - Of the 436 participants in the 2013 South Africa meeting, 126 respondents from 33 countries completed an online survey; none of these respondents were interviewed.   - Most 2012 respondents shared knowledge because they thought that it would be useful to a co-worker or colleague (79.7%) - **Descriptive (activities/outputs)**   - The type of knowledge that respondents most commonly shared was clinical or scientific information (2012: 79.1%; 2013: 66.7%) and country-specific information (2012: 73.0%; 2013: 71.4%); information about journals or publications was not commonly shared (2012: 45.3%; 2013: 32.5%) - **Summative (activities/outputs 🡪 impact)**   - Respondents highlighted that following the workshop they used the information to package knowledge into new products, to design new projects or programs, and to develop training or educational materials   - Most respondents used new knowledge gained from meetings to advocate for policy change (2012: 65.5%; 2013: 67.5%), improve service quality (2012: 60.1%; 2013: 70.6%), and to design new projects or programs (2012: 60.8%; 2013: 65.1%). | 2012-2013 | 2012 (Bangladesh), 2013 (South Africa | **Focus**: global  Bangladesh, South Africa  Participants of maternal and newborn health meetings held in Bangladesh in May 2012 and in South Africa in 2013. | 1c, 3b | - USAID’s MCHIP (led by Jhpiego), Save the Children’s Saving Newborn Lives (SNL) Program, programs supported by UNICEF and other major donors |
| Ongolo-Zogo, Lavis, 2014 [30] | To describe the evidence-informed health system policymaking activities of two knowledge translation platforms in Uganda and Cameroon through documentary analysis of two historical case studies and an evaluative survey of stakeholders with knowledge of the KTP outputs | - **Descriptive (context)**   - Context for both KTPs in Cameroon and Uganda, respectively, is described with respect to political system features, tiered health system features, and MDG indicators   - Both KT platforms are traced historically to various global focusing events, illustrating how convergence of local and global factors and agents has enabled implementation of in-country efforts to support EIHSP related to health MDGs - **Descriptive (infrastructure)**   - EVIPNet scientific and technical support was of great value to both KTP initiatives   - EVIPNet Cameroon secretariat utilizes a multidisciplinary group of part-time researchers through a teaching hospital with links to the Ministry of Health; two scientists have remained the whole time, but turn-over noted amongst researchers and assistants   - REACH-PI Uganda operates with a small team of full-time staff of public health experts trained as brokers, through a public university; social scientist trained as broker left after 12 months, number of brokers went from one to six between 2009 and 2012; initial stakeholder analyses during grant preparation allowed for participatory priority setting exercises and validation of programs of work and enabling environment for mutually beneficial exchange amongst knowledge brokers, policymakers, researchers, and stakeholders   - Vibrant collaboration established with McMaster Health Forum, Norwegian Knowledge Centre for health services and the South African Cochrane Centre in Cape Town, South Africa (SACC)   - External donors provided financial resources to the KTPs to scale up capacity building, knowledge management, linkage and exchange activities (EVIPNet Cameroon received $US 720,000 during 2006-2012). - **Descriptive (activities/outputs)**   - **Activities:** In total, both KTPs have trained almost 500 policymakers, researchers, and stakeholders to facilitate research push and user pull   - All activities and outputs summarized and categorized based on whether they are capacity building, knowledge management, or linkage and exchange; human capital for EIHSP increased by more than 30 training workshops in Cameroon, Uganda, and other countries to build capacity for policymakers, researchers, civil society groups, and media on EIHSP; 500 stakeholders sensitized/trained by both KTPs   - **Outputs:** *Evidence briefs –* following priority setting exercises, 12 produced in Cameroon and 3 produced in Uganda between 2008-2012; they have been a very time consuming labour, since a few evidence brief have required two full time equivalent positions in one year.   - **Outputs:** *Rapid evidence syntheses –* 6 produced in Cameroon (in 2012) and 73 produced in Uganda (between 2010-2012) in response to stakeholders’ urgent needs   - **Activities:** *Policy dialogues –* 7 in Cameroon and 3 in Uganda with pre-circulated briefs on various topics were well received; REACH-PI and EVIPNet Cameroon helped organize the first international forum on EIHP in LMICs in August 2012 in Ethiopia   - **Outputs:** *Bilingual summaries* – under the effective health care research consortium collaboration with SACC, EVIPNet Cameroon has prepared 12 bilingual summaries and translated into French 24 abstracts of Cochrane reviews in 2011-2012   - REACH-PI Uganda established in 2012 a Uganda clearinghouse for HPSR operating as a one-stop shop of health policy relevant evidence; EVIPNet Cameroon has maintained a website since 2009 giving access to evidence briefs and syntheses.   - Both KTPs have assisted in the development and testing of new tools and resources for evidence-informed health systems policymaking - **Formative (activities/outputs)**   - Respondents agreed that briefs achieved their purpose of presenting the available research evidence on a high-priority policy issues to inform a policy dialogue; they appreciated the different design features but expressed dissatisfaction with briefs not concluding with any recommendations   - For all ten dialogues, respondents felt they achieved their purpose to discuss considerations about a high-priority policy issue to inform an action, appreciated how they were designed, and considered the pre-circulated brief helpful - **Summative (activities/outputs 🡪 outcomes)**   - New spaces for deliberations on priority health policy issues have been created through a network of local and global factors and agents | 2001-2012 | 2012 | **Focus:** multi-national  Cameroon;  Uganda | 1a, 1b, 1c, 2c, 3a | - EVIPNet Cameroon; - REACH-PI Uganda (UNHRO established; collaboration with SURE) |
| Ongolo-Zogo, Lavis, 2015 [31] | To assess changes in the climate for evidence-informed health systems policymaking (EIHSP) before and after the implementation of two knowledge translation platforms in Cameroon and Uganda through governmental policy documents | - **Descriptive (context)**   - 54 documents including 17 disease-specific strategic plans and 33 grants funded by global health initiatives were reviewed   - Low profile of evidence syntheses remained stable in both countries in terms of budget allocations and use to frame poverty and health problems   - Burden and determinants of poverty and health conditions were described instrumentally by data, information, or research in the development of policy documents   - Evidence synthesis (e.g., systematic reviews) has remained sparse, under supported, and undervalued over time across countries in terms of framing poverty and health problems, selecting strategies, and forecasting expected outcomes - **Summative (activities/outputs 🡪 outcomes)**   - Increase over time across countries in research-related words and constructs as shown by usage statistics – for example, in Uganda, a higher count was observed in second period (P=0.045); this difference was not significant in Cameroon.   - Utilization of research related cluster value of research in the national development plans increased in the second period for Uganda, whereas in Cameroon the cluster value of research in the health sector strategic plans substantially increased   - Increased influence of external donors (e.g., World Bank, UNFPA) through policy transfer through consistent naming of elements pertaining to climate for EIHSP as demonstrated in many governmental policy documents   - Increased research budgets in both Cameroon (28.496 to 95.467 million Euros, 335% increase) and Uganda (38.064 to 58.884 million USD, 155% increase) with majority of resources going towards monitoring and evaluation (M&E) of health sector performance. In only a few cases financial resources were allocated to support systematic reviews and evidence syntheses. - **Summative (activities/outputs 🡪 impact)**   - However, evidence was used in the strategic paper to reduce maternal mortality in Uganda | 2001-2006 and 2007-2012 | 2012 | **Focus:** multi-national  Cameroon;  Uganda | 1a, 3a, 3b | - EVIPNet Cameroon; - REACH-PI Uganda |
| Rispel and Doherty, 2011 [32] | To summarize the experience of South Africa’s Centre for Health Policy (CHP) in producing knowledge and supporting health systems development, by using a combination of document reviews and key informant interviews. | - **Descriptive (context)**   - Intellectual evolution was assessed using five phases, with four factors influencing this evolution: …political environment and receptiveness to research, funding environment, and evolution of research expertise in country   - Another challenge to sustaining policy impact is declining public profile in some areas - **Descriptive (infrastructure)**   - Challenges to sustaining policy impact include unpredictable funding sources and staff attrition   - Intellectual evolution assessed using five phases interacting with four factors: human resource capacity within CHP… - **Descriptive (activities/outputs)**   - Capacity building has been central to CHP’s success, training close to 100 junior researchers since its inception, many of whom move to government positions and influence policymaking processes (e.g., supply of skilled staff to the National DoH)   - Activities/outputs contributing to the impact included meetings, workshops, disseminating research reports, publishing research in journals, conference participation, producing policy briefs, and engaging stakeholders in policy discussions; some media strategies - **Formative (activities/outputs)**   - Lessons learned from these experiences include conducting high quality relevant research using ethical values, building open and honest relationships with government, adapting to policy environment changes, building capacity as a continuous program, and seeking further funding to ensure independent research and accountability - **Summative (activities/outputs 🡪 outcomes)**   - Policy influence successes include trustworthiness with CHP and its research, strategic networking, and capacity building (i.e., training future policymakers) - **Summative (activities/outputs 🡪 impact)**   - CHP’s research has contributed to domestic health policy development in South Africa and changed the way policy issues are approached; some activities undertaken include meetings, workshops, disseminating research reports, publishing research in journals, conference participation, producing policy briefs, and engaging stakeholders in policy discussions; some media strategies   - Many examples of CHP research that impacted domestic policy categorized by research theme and type of impact including: analyses of level and distribution of health care expenditure, evaluation of social/national health insurance proposals, development of models/norms/costing of comprehensive PHC, etc. | 2010 | 2010 | **Focus:** national  South Africa | 1a, 1b, 1c, 2c, 3a, 3b | - South Africa’s CHP |
| Shroff, Aulakh, 2015 [33] | To reflect upon the experience of projects undertaken by the multi-country SNP (sponsoring national processes for evidence-informed policymaking in the health sector of developing countries) program; to understand why projects in certain settings were perceived by key stakeholders to have made progress towards goals, whereas others were perceived not done so well; to illustrate learning points from experiences across 5 countries to inform future evidence-to-policy efforts in LMICs | - **Study Overview**   - There was variation across the 5 projects with respect to activities carried out, their intensity, as well as perceptions of policy briefs, policy dialogues, and outcomes   - The project in Cameroon and Nigeria (high-performing) and Bangladesh and Zambia (average-performing) were characterized by the coming together of a number of domains identified by Jacobson’s theoretical framework; Argentina is an example of a poorly-performing project   - The domains of Jacobson’s theoretical framework are relevant to the LMIC context but may need to be modified to be fully applicable to these settings, specifically given the relative fragility of institutions and significant role of individual leaders in the evidence-to-policy process - **Descriptive (infrastructure)**   - **Nigeria:** capacity strengthening activities included…creation of a health policy advisory committee that brought together researchers/policymakers/stakeholders to produce policy briefs/dialogues and services for the ministry of health…   - **Nigeria:** project evaluation highlighted the role of team leadership that facilitated building links with stakeholders who were involved in identification of priority areas   - **Bangladesh:** no evidence of a formal mechanism to bring together researchers/policymakers - **Descriptive (activities/outputs)**   - **Nigeria:** capacity strengthening activities included workshops for policymakers on evidence-informed policymaking skillsets (6 workshops for 92 policymakers), creation of a health policy advisory committee that brought together researchers/policymakers/stakeholders to produce policy briefs/dialogues and services for the ministry of health, and establishing an Executive Training Program on evidence-informed policymaking for state policymakers; 2 policy briefs and 2 dialogues organized   - **Cameroon:** the project created a directory of institutions/researchers/stakeholders, training workshops for priority setting and policy brief development (6 workshops for 42 policymakers and 2 researchers), and establishment of a clearinghouse to access policy briefs and relevant evidence   - **Cameroon:** 5 policy briefs were produced and 4 organized policy dialogues.   - **Zambia:** the objective of organizing Research to Action groups were not institutionalized and ended at the project’s completion   - **Bangladesh:** little evidence of capacity strengthening for policymakers using evidence   - **Bangladesh:** 4 policy briefs produced and no organized policy dialogues   - **Argentina:** eight policy briefs were produced, four policy dialogues were organized   - **Zambia:** 3 briefs, 3 dialogues, formation of research to action group to make policy briefs - **Formative (activities/outputs)**   - **Bangladesh:** brief on strengthening public sector commitment to address non-communicable diseases was highly regarded and considered as a ‘timely’ document.   - **Zambia** (Zambian Forum for Health Research (ZAMFOHR)): majority of respondents believe that policy briefs and dialogues achieved desired objectives (3 policy briefs and 3 policy dialogues).   - **Cameroon:** respondent perceptions on project activities and outputs were largely positive   - **Nigeria** (Innovative Health Research Group, Ebonyi State University)**:** 85% of respondents believed the policy briefs and dialogues achieved their goals (2 policy briefs and 2 policy dialogues) - **Summative (activities/outputs 🡪 outcomes)**   - **Nigeria:** the Nigerian Strategic Health Plan underscored evidence-informed policies, which is an example of their impact on the policymaking process in the country   - **Bangladesh:** 73% of individuals involved in project outcomes evaluation believed that the project increased access to research evidence and cemented relationships between researchers and policymakers - **Summative (activities/outputs 🡪 impact)**   - **Cameroon** (Centre for the Development of Best Practices in Health (CDBPH)): two policy briefs were described by the project leader to have directly influenced national policies   - **Bangladesh** (International Centre for Diarrhoeal Disease Research (ICCDR,B)**:** policy briefs were assessed to have major impact on specific national policymaker decisions | 2008-2011 | 2008-2011 | **Focus:** multi-national (funded by the Alliance)  Argentina, Bangladesh, Cameroon; Nigeria, Zambia | 1b, 1c, 2c, 3a, 3b | *SNP programme projects (supported by the AHPSR):*   - CIPPEC; - ICDDR,B; - CDBPH; - Innovative Health Research Group; - ZAMFOHR. |
| Uneke, Ezeoha, 2015 [34] | To study the value of policy briefs and dialogues as policymaking mechanisms that allow policymakers to adapt effective evidence-informed policies for infectious diseases of poverty (IDP) control, by analyzing the results of a questionnaire at the end of the policy dialogue | - **Study overview:**   - 47 policymakers participated in the dialogue; 17.1% were from the Ministry of Health, and up to 48.8% of participants were policymakers that directly impact policymaking processes - **Formative (activities/outputs)**   - **Outputs:** Mean ratings (MNRs) of responses to the policy brief mostly ranged from 6.40-6.85 (moderately helpful to very helpful) on a 7-point scale regarding context/features of the problem, policy options, and key implementation considerations   - **Outputs:** Overall, the policy brief had MNR at 6.54 (very helpful)   - **Activities:** MNRs of responses to the policy dialogue mostly ranged from 6.50-6.82 (very helpful), regarding level of policy issue priority, opportunity to discuss features of the problem, and options to address the problem   - **Activities:** Overall, the policy dialogue was assessed to have MNR at 6.72 (achieved) | 2014-2015 | 2015 | **Focus:** sub-national  Ebonyi State (Nigeria) | 2c | - Ebonyi State Health Policy Advisory Committee (ESHPAC) |
| Uneke, Ezeoha, 2012 [35] | To study efforts to promote EIHP and improve policymaker and stakeholder capacity to use evidence effectively; to study efforts to encourage linkage and exchange between policymaking process players and bridge the policymaker-research gap, through a one-day workshop to improve capacity for using evidence for policymaking | - **Descriptive (context)**   - Researchers acknowledge that evidence was important in policymaking, although only one researcher had participated in processes before - **Descriptive (infrastructure)**   - Focus group discussion identified infrastructure that required improvement, and strategies to bridge gap between policymakers and researchers, such as involvement of both parties in planning/implementation of research and programmes, facilitating dialogue, acquiring research grants and increasing research in health ministries, and ensuring research done on policymaker needs   - Participants identified that further debate on partnership and collaboration between researchers and policymakers in LMICs needed - **Descriptive (activities/outputs)**   - 87 of the 104 individuals invited attended (83.6%) the forum.   - Workshop training sessions were centred on capacity building for EIHP and facilitating linkages/partnerships - **Summative (activities/outputs 🡪 outcomes)**   - Post-workshop assessment showed improvements in knowledge of participants, understanding of policymaking, and use of evidence | 2009 | 2009 | **Focus:** sub-national  Ebonyi State (Nigeria) | 1a, 1b, 1c, 3a | - ESHPAC |
| Uneke, Ndukwe, 2015 [36] | To study the capacity of the Ebonyi State Health Policy Advisory Committee (ESHPAC) and equip its members with the competencies required for effective promotion of evidence-informed policymaking and function as a KTP via analysis of interviews following a series of HPAC capacity building initiatives | - **Descriptive (activities/outputs)**   - Multiple interventions were implemented including: a capacity enhancement training workshop on KT, a three-month certificate course for HPAC, and development of a policy brief and hosting of a multi-stakeholder policy dialogue - **Formative (infrastructure)**   - Participants identified the need for the institution of a performance measurement mechanism and sustenance mechanism for the HPAC to allow it to be more independent in carrying out its evidence-to-policy advisory role - **Formative (activities/outputs)**   - Participants also indicated that the training programme allowed the researchers who were trained to be more proactive in promoting use of evidence in policymaking - **Summative (activities/outputs 🡪 outcomes)**   - There was improvement in knowledge about evidence-to-policy links, KT, and operationalization of KT amongst HPAC members   - Findings indicate elimination of mutual mistrust between researchers and policymakers (and improved relationships), and increased awareness of the importance of HPAC in the ministry of health   - Participants indicated that capacity had been built for policymakers to access and use evidence, understand their need to work with researchers | 2011-2013 | 2013 | **Focus:** sub-national  Ebonyi State (Nigeria) | 1c, 2b, 2c, 3a | - ESHPAC |
| Yehia and El Jardali, 2015 [37] | To examine the process of influencing the mental health policy agenda in Lebanon through use of KT tools and a KTP (Knowledge to Policy Center [K2P]) as an intermediary between policymakers and researchers. Semi-structured interviews and dialogues were used. | - **Study overview:**   - Findings from key informant interviews and synthesis of the evidence allowed for development of the policy brief which presented a problem definition and 3 potential solutions   - The policy brief was circulated to 24 participants before the dialogue to inform the discussion (of which 9 completed the policy brief evaluation survey), and the dialogue validated evidence synthesized in the brief - **Descriptive (activities/outputs)**   - Many KT tools were used to facilitate the evidence-to-policy process: priority-setting, development of a policy brief to address the mental health policy issue at hand, semi-structured interviews with key informants and policymakers, convening a national policy dialogue, evaluating the policy brief and dialogue, and a post-dialogue survey - **Formative (activities/outputs)**   - The element that was most supported by evidence and dialogue participants was integrating mental health into primary healthcare (PHC) services   - Graded-entry format of the policy brief used in the policy dialogue was the most helpful aspect mentioned by participants.   - Participants found helpful the pre-circulation of the policy brief before the dialogue, and the engagement of a facilitator to assist with the deliberations. - **Summative (activities/outputs 🡪 outcomes)**    - Relationships between policymakers, researchers, and stakeholders were strengthened; future meetings and workshops were held independently to discuss implementation   - Awareness of and demand for KT tools amongst policymakers, researchers, and stakeholders increased - **Summative (activities/outputs 🡪 impact)**   - 4 of 6 purposively selected stakeholders completed a post-dialogue survey which showed that 6 months after the dialogue, stakeholders took various implementation steps including establishing a national taskforce, PHC staff training, and incorporating psychiatric medications into the national essential drug list | 2013-2014 | 2014 | **Focus:** national  Lebanon | 1c, 2c, 3a, 3b | - Knowledge to Policy Center (K2P) in the American University of Beirut |
| Zida, Lavis, 2017 [38] | To describe the process of institutionalization of the rapid response service (RRS) in Burkina Faso, and assess the extent of its institutionalization (through qualitative case study consisting of interviews and documentary analysis) | - **Descriptive (infrastructure)**   - The RRS in Burkina Faso has reached the consolidation phase (phase four of five) of the service institutionalization process, but has not reached maturity   - Designated project leaders provided impetus for the project, convincing policymakers of the importance and usefulness of the RRS (introducing the service during an EVIPNet annual meeting in 2010) and acquiring resources to pilot the RRS; the pilot phase began before the policy process was complete   - National and subnational policymakers began to use the RRS at the expansion stage, showing initially that “use of ‘simple rules’ is acceptable without a formal mandate such as a decree” until they developed written guidelines for the service and an official mandate was written   - In 2012, the service was included in the official organization chart of the Ministry of Health general directorate for health information and health statistics (DGISS); this allowed for acquisition of more resources, and potentially enabled the service to obtain state budget in future   - Stakeholder interest was increased during the awareness and pilot phases through sharing practices from Uganda (i.e., sharing an example brief from Uganda); the unit worked closely with Uganda’s RRS between March to December 2011 (experimental phase)   - Early successes in delivery led to increased expectations and demand amongst stakeholders, demand which became difficult to manage due to insufficient resources (e.g., of 64 questions, the unit was able to respond in a timely manner to just 51 [79.7%])   - Unit staff attempted to improve delivery of the RRS based on what was learnt during the pilot stage   - The RRS institutionalization has slowed at the consolidation stage and has not moved into the final maturity phase, and resource requirements for the service varied across phases   - To reach maturity, various investments are recognized as essential (e.g., building capacity, experimentation, English-language learning, and support for institutionalization)   - The service was seen as too expensive at the awareness phase by high-level decision-makers, but the unit was able to demonstrate activities that would save money; costs for the awareness (US$16,500) and experimentation (US$13,095) phases were covered entirely by the EU, specifically for salaries, capacity building, to learn from experience elsewhere, meetings, advocacy, communications, guideline development, and internet connections, among others; costs for the expansion phase (US$23,616) were also fully funded by the EU, although ministry resources were also used   - The unit has now moved into the consolidation stage of the framework, with a budget of US$6345 for staff salaries, office, and equipment funded by the government; there was no internet connection, no additional funds, and no budget line from the ministry of health   - Since June 2013 there has been instability within and outside of the unit due to changes in the home directorate of the unit - **Descriptive (activities/outputs)**   - The unit began producing two briefs per month during the pilot phase   - The unit worked closely with Uganda’s RRS between March to December 2011 (experimental phase) to produce and deliver 5 briefs to four central-level policymakers   - Brief production increased through the expansion and consolidation phases, and the briefs are used for decision-making although monitoring and evaluation of this is not easy to do; in the expansion phase, “23 policymakers sent 78 questions in 44 months”, on average 1.7 questions per month - **Formative (infrastructure)**   - The leadership and advocacy of unit staff and personnel (e.g., the unit’s managers) were key to its successes in the expansion and consolidation phases - **Formative (activities/outputs)**   - Other briefs that were produced following early successes in pilot and experimental phases did not meet user expectations for “speed, quality, and contextualization”   - Policymakers indicated that one facilitator of the unit’s survival was sustained production of reports that were policy-relevant; initially, one policymaker had used a rapid response brief with success at a policy meeting; another policymaker highlighted that maintaining quality of the service by providing answers to the right questions in the right time-frame | March 2011-August 2015 | 2015 | **Focus:** national  Burkina Faso | 1b, 1c, 2b, 2c | - SURE health policy rapid response unit, Burkina Faso |

1. Citations for references presented in column 1 correspond to numbered reference list presented in Additional file 3 [↑](#footnote-ref-1)
2. Domains of analytical synthesis framework addressed:

   - 1=descriptive; 1a=context, 1b=infrastructure, 1c=activities/outputs
   - 2=formative; 2a=context, 2b=infrastructure, 2c=activities/outputs
   - 3=summative; 3a=activities/outputs🡪 outcomes, 3b = activities/outputs 🡪 impact
   - 4=linkages; 4a = context🡪infrastructure, 4b = context🡪activities/outputs, 4c= infrastructure🡪activities/outputs; 4d(o/i)=context affects activities/outputs🡪 outcomes/impact, 4e(o/i)=infrastructure affects activities/outputs🡪 outcomes/impact

   [↑](#footnote-ref-2)
